# Atmospheric Water Harvesting Through Polymer Hydrogel Sorbents: Materials Chemistry, Design Strategies, and Applications

**DOI:** 10.1002/advs.77343

**Published:** 2026-08-27

**Authors:** Swagata Datta, Sushmit Sen, Nilanjan Dey, Amrita Chatterjee, Ananya Sadhu, Sudipta Sarkar, Pradip K. Maji

**Affiliations:** ^1^ Department of Polymer and Process Engineering, Indian Institute of Technology Roorkee Saharanpur Campus Saharanpur India; ^2^ Department of Civil Engineering Indian Institute of Technology Roorkee Roorkee Uttarakhand India

**Keywords:** atmospheric water harvesting, hygroscopic salts, smart hydrogel, solar‐driven water harvesting, sorption‐desorption, stimuli‐responsiveness

## Abstract

The depletion of freshwater resources is a global problem threatening human health and daily chores. The limited availability of freshwater from ground or any other water body throughout the world positions atmospheric water harvesting as one of the vital alternatives. Sorption‐based atmospheric water harvesting (SAWH) draws considerable attention for on‐site synthesis. After moisture absorption, a hydrogel can yield water with minimal input of energy. That's how SAWH unlocks a perpetual source of clear water, which can potentially achieve the global demand for diverse uses. This review paper explores the chemistry of hygroscopic polymeric hydrogel, stimuli‐responsive sorbents, structural stability, performance metrics (swelling capacity, sorption kinetics, thermodynamics), regeneration energy analysis, network design strategy, crosslinking density, the importance of co‐polymerization and bio/synthetic variants. Furthermore, how a stimuli‐responsive material facilitates a low‐enthalpy and field‐assisted uninterrupted desorption while addressing the key challenges related to salt leaching, cyclic fatigue is also a part of the discussion. The current article further highlights the present challenges related to the applicability of this on an industrial scale. This study will be concluded by highlighting futuristic opportunities for the rational engineering of energy‐efficient, high‐performative hydrogels for water harvesting.

## Graphical abstract

Polymer‐based hydrogels have emerged as promising materials for atmospheric water harvesting (AWH) due to their high water affinity, tunable network structure, and excellent moisture adsorption‐desorption performance. Functional hydrogel systems enable efficient water capture and controlled release under low‐energy conditions, offering a sustainable and scalable approach to mitigating freshwater scarcity.

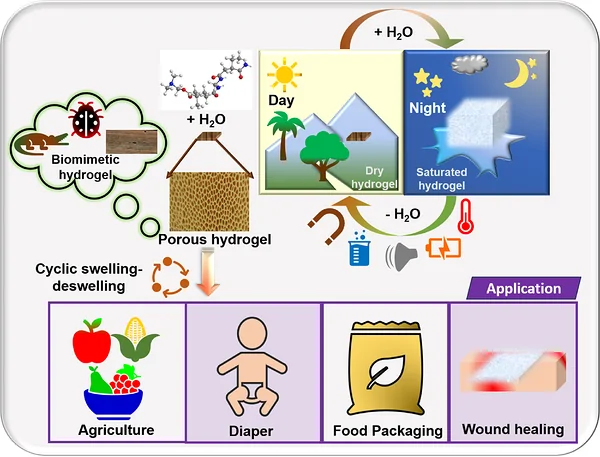

**Abbreviations**
AAc: Acrylic Acid
AAM: Acrylamide
AMPS: 2‐acrylamide‐2‐methylpropanesulfonic Acid
AN: Acrylonitrile
COF: Covalent Organic Framework
DMAAm: N’, N’‐dimethylacrylamide
DMAPAA‐Q: 3‐acrylamidopropyl [Trimethylammonium] Chloride
FCNTs: Functionalized Multiwalled Carbon Nanotubes
HIPG: Hygroscopic Interconnected Porous Hydrogel
HPC: Hydroxypropyl Cellulose
HPMC: Hydroxyl‐rich Hydroxypropyl Methylcellulose
HWT: Hydrogel Water Tracks
IPN: Interpenetrating Network
KGM: Konjac glucomannan (KGM)
LCST: Lower Critical Solution Temperature
MBA: N, N‐methylene‐bisacrylamide
MMA: Poly(methyl methacrylate)
MOF: Metal‐organic Framework
NaSS: Sodium 4‐vinylbenzenesulfonate
NSG: Nanostructured Hydrogel
PAA: Polyacrylic Acid
PAAS: Porous Sodium Polyacrylate
PANSAP: Poly(acrylamide‐co‐potassium acrylate)
PDMAPS: Poly‐[2‐(Methacryloyloxy) Ethyl]Dimethyl‐(3‐sulfopropyl) Ammonium Hydroxide
PDOMA: Poly(dopamine methacrylamide)
PEG: Polyethylene Glycol
PMEDMAH: Poly([2 (Methacryloyloxy) Ethyl] Dimethyl‐(3‐sulfopropyl) Ammonium Hydroxide
PNIPAM: Poly‐N‐Isopropylacrylamide
POEGMA: Poly((oligoethylene glycol) methacrylate)
POG: Photothermal Organogel
PPY: Polypyrrole
PSBMA: Poly (sulfobetaine methacrylate)
PVA: Polyvinyl Alcohol
PZS: polyzwitterionic Salt
SAPO‐34: Silico‐aluminophosphate‐34
SAWH: Sorption‐based Atmospheric Water Harvesting
THMPH: Tunable Hygroscopic Mix‐charged Polyzwitterionic Hydrogel
UPy: 2‐ureido‐4[1H]‐pyrimidinone
VCL: N‐vinyl Caprolactam
WRC: Water Retention Capacity

## 1 Introduction

**FIGURE 1:**
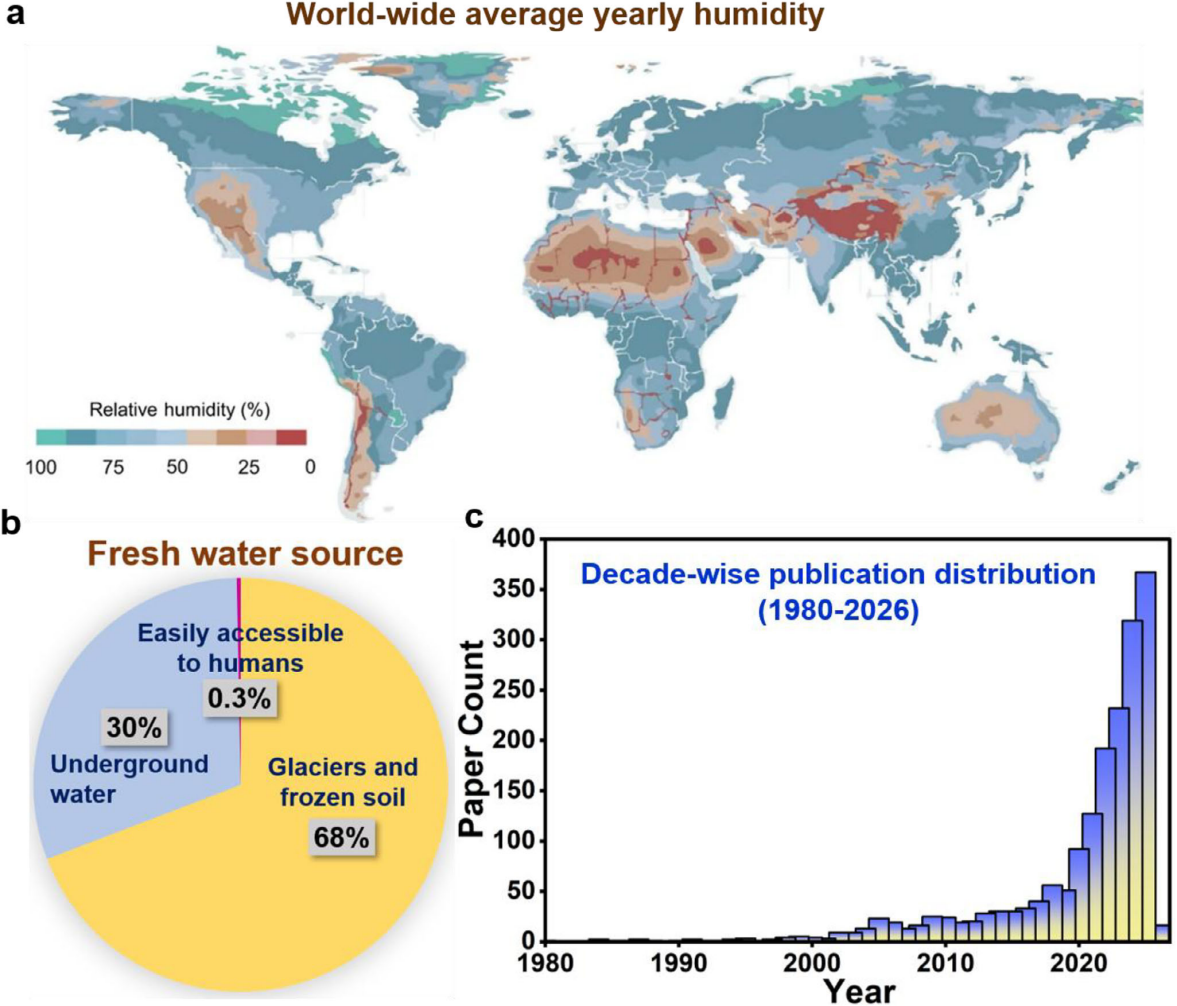
a) Worldwide distribution of yearly average relative humidity. Regions with humidity less than 40% are shown in warm tones (toward red from brown). Reproduced with permission from ref. [3]. Nature, copyright 2022. b) Global distribution of fresh water sources, data taken from ref. [1]. c) Rapid growth trend of research on atmospheric water harvesting by using hydrogel through the years, data obtained from Scopus (accessed on fifth December 2025).

Worldwide freshwater scarcity is undoubtedly one of the most critical challenges of the 21^st^ century, driven by the gap between limited water storage and a rapidly growing population. According to the World Health Organization, among the 8 billion people worldwide, nearly half of the population lacks safe sanitation facilities, more than one‐third are deprived of hand‐cleaning facilities, and one‐fourth of people still do not have access to safe drinking water [1, 2]. Figure 1a represents the worldwide distribution of yearly average relative humidity. Regions with humidity less than 40% are shown in warm tones (toward red from brown) [3]. Research indicates that by the end of 2030, the global demand for freshwater will exceed available supply by 40% [4]. A report published by the US Environmental Protection Agency showed that among the 3% of freshwater resources in the world, almost 68% of freshwater is stored in frozen form either in glaciers or frozen soil, another 32% is reserved as underground water, and less than 0.5% of total freshwater is accessible for human use (Figure 1b). To address this crisis, atmospheric water harvesting technologies have been developed to utilize the vast water source present in the Earth's atmosphere [5]. Figure 1c, represents the increasing trend of research on atmospheric water harvesting using hydrogels over the years, which has increased by ∼ 3800% from 2000. Earlier approaches primarily relied on passive and active water‐harvesting technologies, including 2D or 3D mesh‐based systems, as well as large‐scale technologies requiring external input of energy, respectively [6, 7]. However, the passive system works discontinuously, performs efficiently only at elevated relative humidity (> 40%) and exhibits limited performance in arid environments (relative humidity < 30%) [8]. Moreover, the electromagnetic and absorption cooling technologies are constrained by high energy consumption and operational cost [1].

**FIGURE 2:**
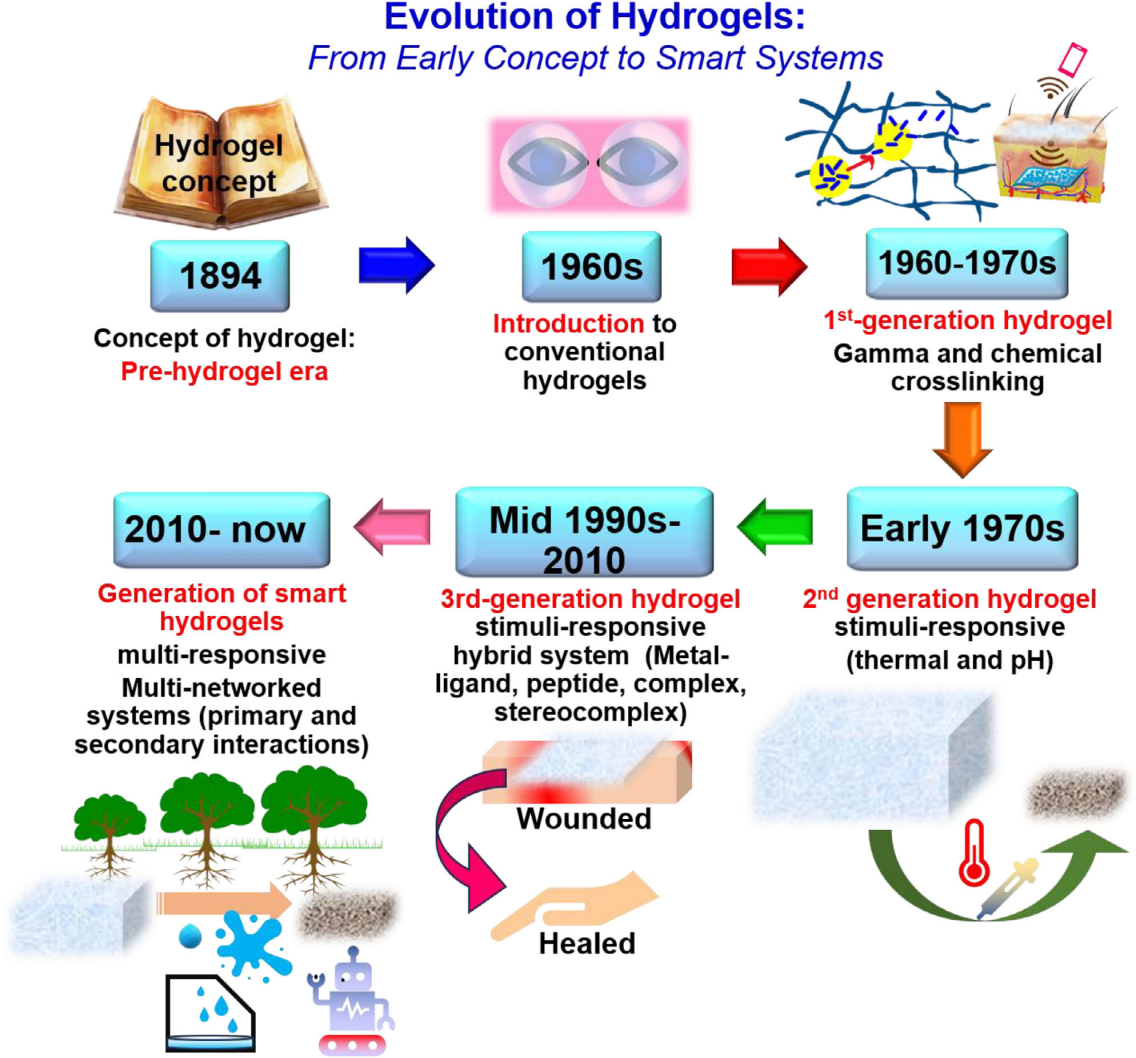
Evolution of Hydrogel Systems from Concept to Smart Hydrogel.

Hence, attention has shifted toward sorption‐based water harvesting systems (semi‐active water harvesting system), particularly on gel‐based systems which combine high hygroscopicity, minimal desorption enthalpy and structural integrity, mainly in1960s. Hydrogels have evolved from simple chemically crosslinked systems to single stimuli‐responsive to advanced supramolecular networks, offering tunable network structure, mechanical robustness, and increased hygroscopicity‐ properties that are relevant for advanced water harvesting from the atmosphere in the year of 1960s and late 1970s [9, 10, 11]. Following Katchalsky's pioneering research work, scientists converted chemical energy into mechanical energy during the 1950s and 1960s. Thus, in the early 1970s the second generation of hydrogels emerged with the introduction of stimuli‐responsive (temperature, pH) polymeric networks for application in controlled drug delivery, tissue engineering, pharmaceutical formulations and the biomedical field [12, 13, 14]. In this generation more than two polymers were employed to construct a hydrogel in the form of a crosslinked, interpenetrated network, or copolymerized system. These hydrogels are predominantly formed through physical or radiative crosslinking, hydrophobic‐hydrophilic interactions, micellar aggregation, etc [15]. By the mid‐1990s, in situ interaction, metal‐ligand coordination, peptide crosslinking, complex and stereo‐complex formation, supramolecular networks were considered for hydrogel preparation. Polymers such as PEG/PLLA, PEG/PDLA, chitosan, dextran, peptide‐based polymers, alginate are often used in combinations to synthesize backbones [16, 17, 18, 19, 20]. Owing to their mechanical robustness, thermal and chemical stability, degradation properties have been widely used in tissue engineering, biomedical applications, drug‐releasing applications, etc. This is the time period for the third generation hydrogel [11]. These newly‐aged polymer‐based hydrogels facilitate the swelling ability of 0.64 g g^−1^ even at 15% RH, and per day can harvest ∼ 5.5 L Kg^−1^ day^−1^ at relative humidity ranging from 10.6‐41.6%, which is sufficient to fulfil the basic needs of a human [3]. Further development in technology and the introduction of more than two organic or inorganic materials in the polymer matrix shifted the focus from simple hygroscopic polymeric hydrogels to “smart” or “intelligent” hydrogels. “Smart” hydrogels represent an advanced class of chemically or physically crosslinked, nontoxic hydrogels capable of responding under multiple stimuli. The development of an “intelligent” hydrogel requires cautious material selection and should be tailored according to the desired properties, including mechanical robustness and controlled desorption rate [21]. In this process, thermo‐, photo‐, magneto‐, and electro‐responsive materials get stimulated by external temperature or applied field, increasing the system's temperature and disrupting the water‐hydrogel physical interaction and inducing desorption [22, 23, 24]. Chronological evolution of hydrogel systems, from the conceptual level in year of 1960s to the era of smart or intelligent hydrogel in 2026, has been comprehensively represented in Figure 2.

**FIGURE 3:**
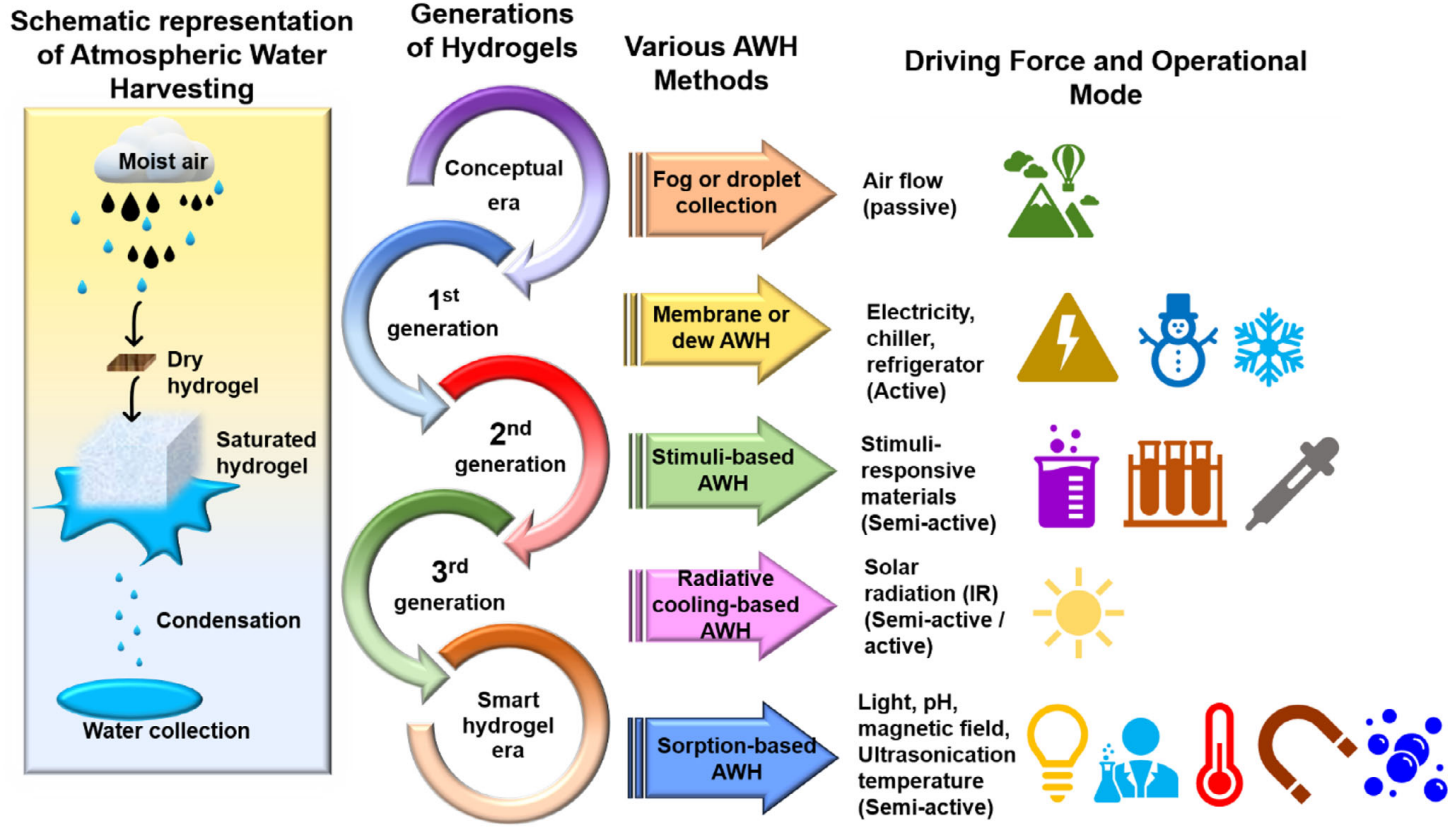
Schematic representation of the correlation of the atmospheric water harvesting (AWH) system with generations of hydrogels, water harvesting mechanism, driving force and various operation modes.

The SAWH systems can operate in arid regions with very low humidity only with the input of hygroscopic and stimuli‐responsive materials, and temperature from any source of energy for desorption. Scientists also tried to use the hydrogels as a potential source of fresh water feed in the agricultural and biomedical applications [25]. In the field of “smart” or “precision” farming, hygroscopic hydrogels established their promising potential as self‐sustaining water reservoirs by harvesting water from the atmosphere, storing it within the 3D scaffold and releasing it in a controlled manner to the plant root [26]. Water harvesting technologies can also be coupled with an ultrasonic vibrator, thermal management systems [27]. Sorbent composition critically determines the properties, efficiency and cyclic stability of the system [28, 29, 30, 31]. Being super‐hygroscopic in nature, synthetic polymeric materials based on acrylic acid or acrylamide are mainly used in semi‐active systems by crosslinking with other materials MOF, graphene oxide, carbon nanotubes, magnetic nanoparticles, modified polymers) or by forming an interpenetrating network structure [32]. Incorporation or crosslinking of foreign materials in the hydrophilic polymers synergistically helps in water uptake capability, mechanical properties, water adsorption and desorption kinetics. Despite all of these advantages, several challenges remain associated with these systems, such as complexity in manufacturing, which increases the cost and limits commercial scalability. Although these systems are reusable for multiple sorption cycles, but approximately after 20–25 cycles the sorption rate slows down or other malfunctions may arise [21]. This review paper systematically analyzes the chemistry and principles of the water sorption process in polymeric hydrogel‐based systems. We also emphasized the structure‐property relationship by examining the mesh or pore morphology, crosslinking density, various stimuli responsiveness, processing, applications, key challenges and future perspectives. A comparative summary of atmospheric water harvesting (AWH) systems, the operating relative humidity range and specific energy consumption required for that particular process has been discussed in Table 1. A visual representation of the AWH system, its evolution through various generations, and the corresponding driving force governing the underlying operation has been represented in Figure 3.

**TABLE 1:** Comparison of various technologies of atmospheric water harvesting (AWH) based on operating mechanism, energy consumption and effective relative humidity ranges.

AWH mechanism			Specific energy consumption	Operating relative humidity
Active water harvesting system	Refrigeration [25, 33, 34, 35]		System consumes 830 – 25000 kJ L^−1^ 580 – 830 kJ L^−1^	15%–90%
	Membrane separation [36]		System needs 1080–30492 kJ L^−1^	>50%
	Vapour compressor cycle generator [35]		System needs 1440–27000 kJ L^−1^	15%–95%
	Thermoelectric cooling technology [37]		Complete water harvesting system consumes energy 7.67 MJ L^−1^	≥ 40%–80%
Passive water harvesting system	Rainwater collection [1, 38]		—	20%–100%
	Fog capture by using mesh [39]			50%–100%
	Dew collection [1]			>100%
Semi‐active water harvesting system	Polymer‐based hydrogels	Thermally sensitive [40]	Thermal energy (LCST, 35− 45°C)	15%–95%
		Magneto responsive [23]	Solar irradiation (1440‐7200 kJ m^−2^)	30%–90%
		Photothermal material [41, 42]	Thermal energy (50‐60°C)	20%–80%
		Ultrasonic‐based AWH [27]	470 kJ L^−1^ desorption temperature ≥ 20°C	30%–70%
	MOFs [43]		Solar irradiation of 1–400 kJ/m^2^	30%–90%
	Hygroscopic salt‐based system [44]		Energy consumption per unit volume of salt ∼ 5.6–14.1 kJ cm^−3^	10%–90%
	Zeolite [45, 46]		Regeneration temperature (250‐300°C) 3000‐ 4000 kJ L^−1^	>30%
	Silica‐based sorbents [45, 47, 48]		Regeneration temperature >50°C, * *2300–2500 kJ L^−1^	<10%–80%

## 2 Polymer Chemistry Principles in Water Vapor Sorption

The efficiency of a semi‐active water harvesting system is mostly determined by its capacity of water adsorption and desorption, as well as its kinetics and durability. However, depending on the hydrogel's application field, the sorbent chemistry, architecture, pore density and distribution, morphology, and synthesis process should differ. Hence, this section of the review paper presents a complete picture of molecular chemistry, including recent developments at the molecular level, particularly how moisture is adsorbed, channelized, and stored in a hydrogel. The proper balance and distribution of functional groups determine the efficiency of a system. Polymer‐based hydrogels consist of hydrophilic functional groups such as amine (‐NH_2_), hydroxyl (‐OH), carboxyl (‐COOH), amide (‐CONH‐), acrylamide (‐CONH_2_); all of these groups enhance water sorption capability through physical bonding. These bonds are reversible and non‐covalent, helping to maintain the 3D integrity of the hydrogel's structure and its self‐healing property [49]. These bonds can be triggered by external stimuli or mechanical vibrations, facilitating a reversible transition from a hydrophilic to a hydrophobic state by changing their molecular conformation [27, 50, 51, 52]. Scientists also claimed that the internal osmotic pressure of nonionic hydrogels is subsequently lower than that generated by hygroscopic salt solutions during moisture uptake, resulting in a decreased swelling capacity [53]. The uncharged nonionic hydrogels cannot form electrostatic interactions or coordination sites to bind the embedded hygroscopic salts; consequently, the salts remain highly mobile within the polymer matrix, resulting in salt migration, leakage of salt solution or agglomeration. Such agglomeration blocks the internal diffusion pathway. The moisture adsorption in hydrogel is a two‐step mechanism: 1) First, the moisture is captured and liquified by the hygroscopic groups and salts on the surface of the hydrogel. 2) Then the liquid water subsequently diffuses through the internal portions of the polymeric matrix, where it is stored through swelling of the hydrogel network. Next, according to the environmental conditions, the water gets released [54].

### 2.1 Hydrophilicity and Functional Groups (Amide, Carboxyl, Sulfonate, Zwitterions)

**FIGURE 4:**
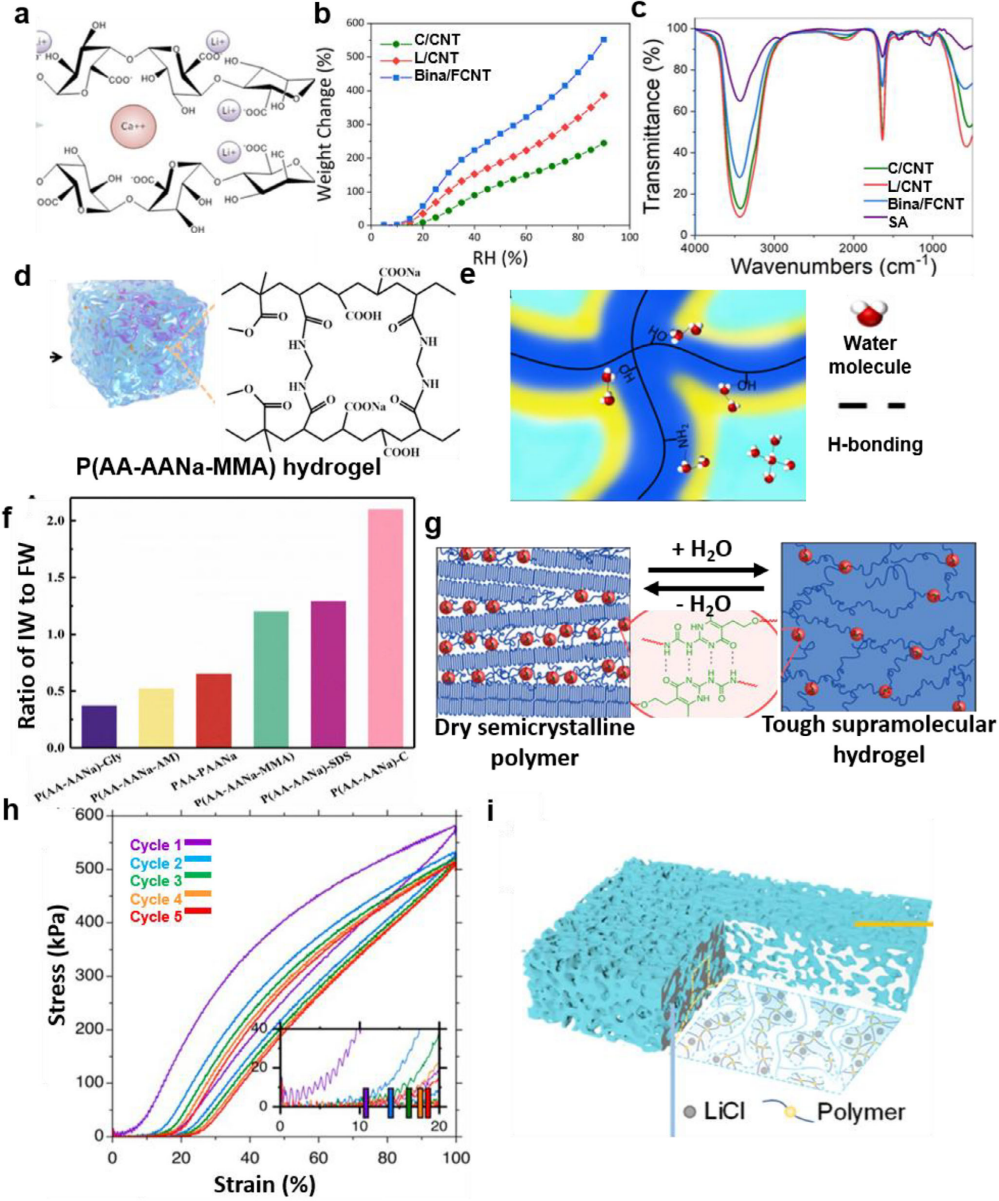
a) Schematic structure of Bina/FCNT (Ca^2+^ and Li^+^ ions from ionic coordination with G‐block and M‐block, respectively) b) Isotherm curve of Bina/FCNT, C/FCNT, and L/FCNT c) FTIR analysis to probe the physicochemical interactions among the salts, alginate matrix and FCNTs. Reproduced with permission from ref [55]. ACS, copyright 2020. d) Schematic representation of crosslinked hydrogel P(AA‐AANa‐MMA), e) Water content in hydrogel, bound water (dark blue), intermediate water (yellow), free water (light blue) f) Decreasing ratio of intermediate water to free water in PAA‐PAANa‐based hydrogel. Reproduced with permission from ref [59]. ACS, copyright 2025. g) schematic representation of supramolecular UPy‐UPy hydrogel network containing reversible hydrogen bonding. h) 5 Cyclic testing at maximum strain 100%, inset illustration showing the residual strain after the very first elongation. Reproduced with permission from ref [64]. ACS, copyright 2014. i) Schematic structure of HIPG with adsorbed water. Reproduced with permission from ref [56]. Nature, copyright 2024.

The primary hydrophilic functional groups present in a polymeric hydrogel are responsible for the completion of a cyclic process of water absorption and storage. A polymeric network contains hydroxyl, carboxylate, amine or amide groups, which help in hydration by forming hydrogen bonding with the adsorbed water molecules and ionic groups, which prevent the leakage and migration of salt [55]. Once the adsorption saturation is achieved, the characteristic crystalline peaks of the double networked hydrogel‐salt disappear, indicating the transition toward an amorphous structure. In an alginate‐based hydrogel, hydrophilicity originates from the abundant hydroxyl and carboxylate groups along the polysaccharide backbone. Figure 4a represents the alginate group and salts embedded with functionalized multiwalled carbon nanotubes (FCNTs), a photothermal agent, which is rich in hydrophilic functional groups such as carboxylate(‐COO^−^) and hydroxyl (‐OH) moieties. The carboxylate group forms ionic and hydrogen bonding with multivalent cations (Ca^2+^ and Li^+^), generating a mechanically stable egg‐box coordination model (Figure 4a) [42]. Entezari et al. established the importance of inclusion of binary salts in a hydrogel, the single‐salt hydrogels suffer from deliquescence and structural instability, whereas the binary salt hydrogel system achieves a significantly higher and stable water uptake owing to the presence of ionic coordination and extensive hydrogen bonding between polymer chains, salts and water molecules (Figure 4b,c) [42]. Compared to a non‐electrolyte hydrogel (< 30 atm), a poly‐electrolyte hydrogel possesses a much higher internal osmotic pressure of 659 atm, which arises due to the presence of dissociated ionic groups and loosely held counter‐ions, generating Donnan osmotic pressure within the hydrogel network. Hence, it is proved that not only hydrophilicity, but also a leakage‐proof hydrogel system is equally important for increasing the adsorption kinetics and overall efficiency [53]. Moisture can only be captured due to the presence of hydrophilic (COO^−^Na^+^) functional groups in the Porous sodium polyacrylate (PAAS) through hydrogen bonding [56].

Researchers also synthesized hydrogels using a double network, interpenetrating network (IPN), semi‐IPN, chemical or physical crosslinking by combining 2 or more polymers bearing various hydrophilic functional groups, which facilitates the adsorption and storage of water molecules [50]. However, high salt loading can induce a salting‐out effect, followed by agglomeration of the polymeric chains and volumetric collapse of the 3D hydrogel network. So, scientists used zwitterionic polymers‐ containing both positively and negatively charged groups along their main chain or side branches. By the addition of salts, these charged functional groups form ionic coordination, hinders the inter‐ or intra‐chain self‐association, resulting in a salting‐in effect rather than the collapse of network. Owing to the presence of oppositely charged ions and the absence of permanent covalent bonding, these ionic interactions are highly reversible. Based on this concept, scientists fabricated zwitterionic hydrogels impregnated with salts, demonstrating excellent water sorption capability over a wide range of relative humidity (15%–90%) with mechanical robustness and structural integrity without any salting‐out effect or leakage of solution [54, 57, 58]. Aleid et al. stated that the hydrogel containing quaternary ammonium (‐N^+^(CH_3_)_2_) and sulfonate (‐SO_3_^−^) of poly‐[2‐(methacryloyloxy)ethyl] dimethyl‐(3‐sulfopropyl)ammonium hydroxide (PDMAPS) generate electrostatic interaction with ions of LiCl salt, respectively and forms an ionic hydration shell. In contrast, conventional amide‐based non‐ionic polymeric hydrogels face salting‐out in the presence of hygroscopic salts, which suppresses the swelling ratio with a limiting water uptake capability [54]. However, synthesization of a hydrogel without the incorporation of any additional hygroscopic salts is also possible; researchers copolymerized a polyelectrolyte component‐ partially neutralized acrylic acid with NaOH (‐COO^−^Na^+^) with polyacrylic acid (PAA). The carboxylate‐rich (Figure 4d,e) backbone of polyelectrolyte hydrogel provides stable electrostatic hydration with the adsorbed water, resulting in the coexistence of bound, intermediate and free water. Also, the incorporation of hydrophobic materials, such as methyl methacrylate (MMA), sodium dodecyl sulfate (SDS) or graphite into the polyelectrolyte matrix, increases the percentage of intermediate water (Figure 4f) and consequently lowers the evaporation enthalpy. The absence of external salt is not a limitation here, as water uptake and release are governed by the electrolyte nature of PAANa‐PAA hydrogel and the molecular state of water present in the hydrogel, not by the salt deliquescence [59]. The representative studies between chemical functional groups present in the polymeric backbone and the performance of the system for water harvesting is provided in Table 2.

**TABLE 2:** Representation of the correlation between chemical functionality and AWH performance.

Material / Hydrogel System	Chemical functionalities	Performance of the system
LiCl/graphene oxide (GO) @PVA‐PAM [55] double network spongy hydrogel	Hydroxyl, amide, carbonyl groups coordinate with ionic salts and adsorbed water	High water adsorption (0.785‐3.507 g g^−1^), desorption efficiency of 3.82 kg m^2^ h ^−1^ under one sun irradiation
Li^+^/Ca^2+^ salt‐modified alginate, incorporated FCNT [42]	Ionic attraction between Carboxylate groups (‐COO^−^) and binary salts	Hydrothermally stable, water uptake (∼ 5.6 g g^−1^)
Cationic polyelectrolyte PAMPS‐CNT‐LiCl hydrogel [53]	Coordination between charged sulphonic group and ionic salts, water adsorption through hydration shell	Water yield of 2410 mL_water_ kg_sorbent_^−1^ day^−1,^ almost 3.5 times of a non‐hydride hydrogel
Foam‐dried interconnected HPMC‐PAAS matrix containing LiCl and photothermal agent (TiN) [56]	Electrostatic attraction between hydroxyl and carboxyl groups of the matrix and ionic salts through ionic hydration and hydrogen bonding.	Excellent indoor water yield 14.9 L_water_m^−2^day^−1^ and 3.5‐8.9 L_water_m^2^day^−1^ in outdoor experiments
PDMAPS‐ zwitterionic polymer containing LiCl‐ salt and photothermal agent CNT [54]	Ammonium groups and sulfonate groups are coordinated with LiCl; water and salt are adsorbed through ion‐dipole and electrostatic interactions	Sating‐in effect, 22 cycles in 225 min, ∼ 0.30 g g^−1^ water uptake at 20% RH and 25°C, * *1.75 g g ^−1^ swelling ratio at 25°C 80% RH, ∼90% desorption of water at 80°C Within 2 h while maintaining swelling ratio 1.26 g g^−1^
Polyzwitterionic PDMAPS‐LiCl hydrogel [57]	Electrostatic coordination between quaternary ammonium and sulfonate groups stabilize the anchored water through hydration shell	Clean water production of 5.87 L kg^−1^ per day at 30% RH, water absorbency of 0.62 g g^−1^ at 30% RH at 120 min
Zwitterionic THMPH (NaSS‐MPTC copolymer)‐LiCl hydrogel [58]	Sulfonate and quaternary ammonium groups form multilevel ionic crosslinking with LiCl and store the bound water	2.9 g g ^−1^ water uptake at 25°C at 70% RH, at 10% saturated LiCl solution swelling ratio is 19 g g ^−1^ (PDMAPS ∼3 g g^−1^)
PAA−PAANa matrix with incorporation of graphite [59]	‐COOH and ‐COO^−^Na^+^ forms hydrogen and ion‐dipole bonding with adsorbed water	Low evaporation enthalpy (2177 J g^−1^) with a rate of 1.39 kg m^−2^ h^−1^

The efficiency of a polymeric hydrogel for atmospheric water adsorption is primarily governed by the distribution and type of hydrophilic functional groups, determining the nature and strength of the secondary interactions. However, their influence on water adsorption, retention and regeneration differs significantly depending on the type of their chemical nature. Hydroxide and amide groups primarily promote reversible hydrogen bonding with water molecules and require relatively low regeneration energy because of their weaker binding energy (∼20.5 kJ mol^−1^, and 7.8 kJ mol^−1,^ respectively). In contrast, ionic groups such as carboxylate, sulfonate and quaternary ammonium ions form stronger (40–100 kJ mol^−1^) ion‐dipole interactions with the water molecules, thereby improving water uptake, even under low relative humidity [60]. On the other hand, zwitterionic polymers, containing both cationic and anionic groups, establish electrostatic interactions (∼ 250–650 kJ mol^−1^) with the ionic salts (LiCl, CaCl_2_, MgCl_2_) and maintain structural integrity of the hydrogel, while preventing salt‐leakage during repeated sorption cycles [42, 60]. During regeneration, while increasing the temperature (∼ 45°C), the supplied thermal energy progressively weakens all kinds of secondary interactions, allowing water desorption while retaining the hydrogel's 3D scaffold [58]. Therefore, an optimal balance between hydrogen bonding, electrostatic and ionic interactions is essential to maximize the water uptake, sorption kinetics, water retention behaviour and maintain long‐term cyclic stability. Excessively strong interactions either increase the regeneration energy or slow the desorption rate.

### 2.2 Hydrogen Bonding & Ion–Dipole & van der Waals Interactions

The moisture sorption mechanism in polymer‐based hydrogels is primarily governed by inter‐ and intramolecular interactions between water and the hydrogel network. Beyond the intrinsic hygroscopicity of the functional groups, their physical interactions such as hydrogen bonding, ion‐dipole, dipole‐dipole, electrostatic forces, and van der Waals attraction play a critical role in absorption, transportation, retention of retained water and, most importantly, they help to maintain the 3D skeleton of the hydrogel after continuous sorption cycles. These interactions critically influence the mechanical strength, self‐healing property, water retention, degradability, cyclic durability and overall structural integrity [58]. Therefore, developing a mechanically robust hydrogel with high strength, ultra‐toughness and self‐healing property requires the integration of multilayered secondary bonding.

Designing of a single network system consisting of multiple monomers requires a precise balance between the hydrophilic and hydrophobic components to maintain structural stability while preventing hydration‐induced disruption, even at relatively low chemical crosslinking densities. A hydrogel composed of a ternary system containing acrylamide (AAm), acrylonitrile (AN), and 2‐acrylamide‐2‐methylpropanesulfonic acid (AMPS), each component plays a distinct role in the hydrogel. The first one is acrylamide (AAm), which induces hydrogen bonding, helps to enhance toughness and structural stability, second one is acrylonitrile (AN), which helps to maintain a strong dipole‐dipole interaction, and the third one is 2‐acrylamide‐2‐methylpropanesulfonic acid (AMPS), an anionic monomer, provides electrostatic repulsion which promotes network expansion by facilitating diffusion of water throughout the network [61].

A similar strategy has been used to design several hydrogel systems in which different physical interactions are integrated to enhance overall performance. A photothermal organogel (POG) based on a hydrophilic copolymeric network consisting of hydrophilic functional groups (‐OH, ‐CONH_2_, ‐COO^−^) capture moisture from air via hydrogen bonding and transports it inside the hydrogel through osmotic pressure [62]. Yang et al. further reported a super‐hygroscopic interconnected porous hydrogel (HIPG) (Figure 4i) by gelation technique, where hydroxyl‐rich hydroxypropyl methylcellulose (HPMC) established a physical crosslinking through hydrogen bonding with carboxylate‐rich sodium polyacrylate (PAAS), which enhances the mechanical stability of the hydrogel, while LiCl also contributes to forming additional ion‐dipole interactions through the hydration of Li^+^ and Cl^−^ ions and improves moisture retention [56]. These findings align with Figure 4e, which indicates the capture and storage of adsorbed water through hydrogen bonding with the hydroxyl and amide groups of the polymer matrix [59]. For the case of zwitterionic and polyelectrolyte‐based hydrogels, electrostatic attraction plays a critical role. A polysaccharide‐based hydrogel network is predominantly governed by hydrogen bonding between PEG and starch molecules, which provides the network's structural integrity. When the polysaccharide domains of the hydrogel become fully saturated by adsorbed water, the hydroxyl groups of the PEG provide additional adsorption sites, facilitating additional water uptake via H‐bonding. Up to an optimum percentage of PEG in the hydrogel, the swelling ratio increases progressively; beyond that point, reduced chain mobility and decreased degree of freedom lead to a reduction in the hydrophilicity of the hydrogel [63]. Further study was done by Guo et al., where they produced a PEG‐based hydrogel, reinforced by (2‐ureido‐4[1H]‐pyrimidinone) UPy‐UPy hydrogen‐bonded (Figure 4g) segregations. This noncovalent secondary crosslinking reinforces the hydrogel network and endorses processibility, whereas long PEG chains promote higher extensibility with low crosslinking density. The mechanical properties of this particular hydrogel are superior to those of many chemically crosslinked hydrogels. The H‐bonded crosslinks enable reversible network expansion, improved processability, and impart shape‐memory behaviour to the hydrogel (Figure 4h) [64]. Zwitterionic hydrogel containing both positively and negatively charged groups generates a strong electrostatic attraction, which stabilizes both the polymeric network, ionic salt and adsorbed water [65]. Similarly, polyelectrolytic hydrogel forms a complexation between charged polymeric ions and hygroscopic salt, which enhances the sorption kinetics, prevents salt leakage and sustains a continuous sorption cycle [53]. At the nanoscale level, ion‐dipole interactions between the zwitterionic polymer and the ionic salts enable efficient moisture adsorption and low‐enthalpy water release [66]. In comparison with hydrogen bonding, electrostatic and ion‐dipole interactions offer a stronger hydration stabilization even in low relative humidity regions.

Van der Waals forces are relatively weaker than electrostatic, ion‐dipole, dipole‐dipole interactions and hydrogen bonding; however, because of their high reversibility, they contribute to network flexibility, elasticity, and self‐healing behaviour rather than network strengthening. Beyond the use of only polymer‐based hydrogels, scientists relied on the combination of all kinds of physical crosslinking. The water adsorption and structural integrity of hydrogels are primarily governed by hydrogen bonding between the polymeric network and adsorbed moisture. This physical interaction of imidazole and the carboxylic acid group with adsorbed water effectively immobilizes the bulk water within the polymer network and enables effective storage of water [67]. A MOF‐based zwitterionic hydrogel further possesses π‐π stacking with ionic interactions, which play a fundamental role in stabilization of the hydrogel by forming reversible physical crosslinking, regulating chain entanglements and immobilizing the water molecules [68, 69]. Similarly, hydrogen bonding between chitosan and the P(AAM‐AA) copolymer inhibits chain mobility and thereby helps in maintaining structural stability [70]. Parallelly, the incorporation of N‐rich additives, such as polypyrrole, promotes the formation of a hydration shell and long‐term water retention capacity. These functional groups, including N, ‐OH, ‐COOH/‐COO^−^, ‐CONH_2,_ interact with water molecules and form hydration shells, leading to the stabilization of the captured water inside the hydrogel matrix. Overall, for effective water sorption and storage, a perfectly engineered hydrogel with a proper balance of physical and chemical crosslinking is required, rather than only hydrophilicity, to maintain perfect mechanical strength, toughness, and structural stability for a prolonged application. The representative studies between dominant molecular interactions and the performance of the system for water harvesting is represented in Table 3.

**TABLE 3:** Driving force and performance relationships in hydrogel systems for Atmospheric water harvesting.

Material / Hydrogel System	Dominant molecular interactions	Performance of the system
AN‐AAm‐AMPS hydrogel [61]	Dipolar interactions and hydrogen bonding	Equilibrium water content (30%–98%) while maintaining tensile strength of 8.3 MPa
PNIPAAm‐PNMA‐@LiCl‐CNT [62]	H‐bonding between polymer and water, thermoresponsive change in conformation	Water uptake starts at RH 15%. Desorption starts within 5 min under sun exposure; per day water yield of 2.43 kg m^−2^ day^−1^, equilibrium water content 16.01 kg m^−2^
PNIPAM‐PDMAPS‐LiCl zwitterionic thermoresponsive hydrogel [66]	Ion‐dipole interaction, hydrogen bonding between water and hydrogel matrix	∼ 12.25 g g^−1^ (optimum inclusion of salt) Moisture uptake at 15% RH, over 80% release of water within 20 min at 40°C
Starch‐acrylamide‐cellulose‐PEG [63]	Hydrogen and covalent bonding	At 16 days, water retention rate is 50.61%, Swelling ratio 80.34 g g^−1^
UPy‐PEG block hydrogel [64]	Increase in strength through hydrogen bonding	Equilibrium water uptake of – 81–86%, Mechanical stability >30 days, cyclic stability after more than 300% deformation
P(VI‐co‐MAAc) copolymer hydrogel [67]	Inter‐ and intra‐molecular hydrogen bonding	∼ 2.18 g g^−1^ at 60% RH; toughness of the hydrogel is maintained 2≤ pH≤ 10
Chitosan/PVA‐vertically aligned MOF [68]	Capillary force, hydrogen bonding	∼ 4.44 g g^−1^ at 90% RH with a rate of 4.38 g g^−1^ h^−1.^ Structurally stable network,
AA‐AAm hydrogel reinforced by chitosan with in situ polymerization of PPy [70]	Hydrogen bonding between chitosan and polymer, polypyrrole and water molecule, electrostatic interaction between salt and hydrogel scaffold	Efficient water harvesting performance, 70% RH and 25°C Water uptake was 1.53 g_water_g_solvent_^−1^ Stable over a wide range of pH, high Young's modulus 170 MPa at moderate water content 50–60 wt.% desorption rate 87.6%,

### 2.3 Role of Ionic Clusters and Ionomeric Networks in Hygroscopicity

**FIGURE 5:**
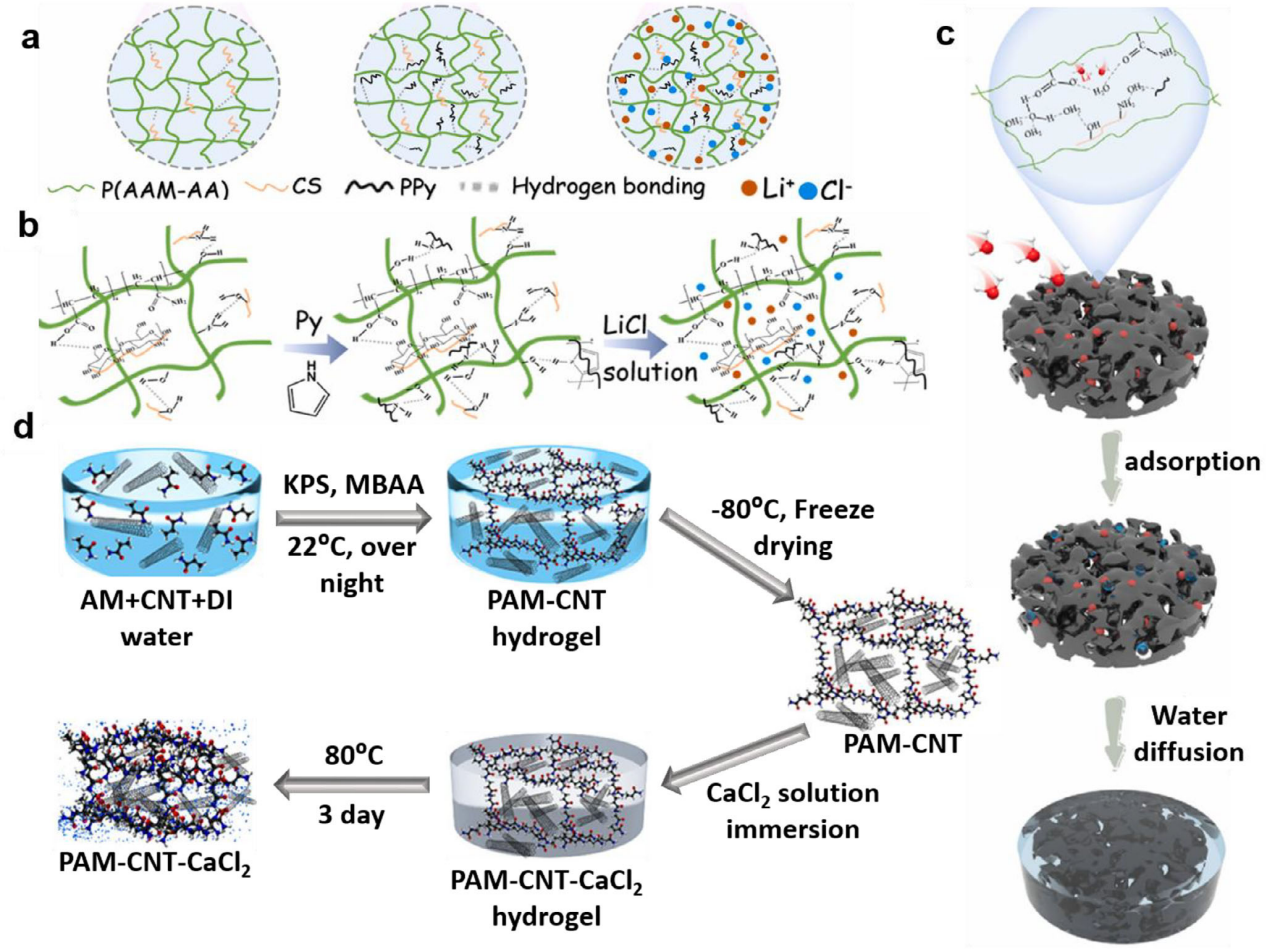
a) Schematic microscopic representation of AAC, PPy‐AAC and PPy‐AAC‐LiCl, sequentially [70]. b) The synthesis principle and intrinsic physical interactions of PPy‐AAC‐LiCl. c) Schematic representation of the hygroscopic process of PPy‐AAC‐LiCl. Reproduced with permission from ref [70]. ELSEVIER, copyright 2025. d) Schematic synthesis process of PAM‐CNT‐CaCl_2_ hydrogel, highlighting the physical interactions. Reproduced with permission from ref [74]. ACS, copyright 2018.

AWH systems can be tailored to have the desired properties in many ways, one of which is the improved design of ionic clusters and ionomeric networks to adjust the hygroscopicity. Ionomers are polymers with ionic groups, which generally form ionic clusters through microphase separation due to the non‐uniform dispersion of those groups in the polymer matrix. A large number of ‐OH groups typically improve the efficacy of the AWH, as found in silica hydrogels [71, 72]. Ionic networks in their hydrated form show osmotic swelling, which leads to an enhanced accessibility to a larger number of ionic sites within the polymeric network, resulting in a rapid water uptake of these AWH systems [72]. Hydrogels containing acidic group(‐COOH), hydroxyl, amide or secondary amine groups attract and retain the ionic hygroscopic salt and water through electrostatic and ionic‐dipole interactions, which is shown in Figure 5a–c, respectively [70]. Lu et al. reported an interesting work on designing a unique oppositely charged hydrogel heterojunction for electricity and water‐harvesting purposes [73]. Upon exposure to moist air, this multi‐functional hydrogel readily absorbs the moisture; in this process, the ions dissociate from its counter polymer chains. These free oppositely charged ions diffuse toward the interfacial region and form an ionic double layer, which induces an electric field directed from the cationic layer to the anionic layer. Opposite polarity and an interfacial electric field enable a long‐lasting power supply of 0.36 nW·cm^−2^ up to 40 h, even under extremely wet conditions (> 90% RH). Incorporation of hygroscopic salts, such as LiCl, enhances their moisture uptake capacity, making this a suitable solution for addressing energy and water crises. Guan et al. describe a similar energy‐cum‐water harvesting system by preparing a LiCl‐loaded zwitterionic microgel with poly‐N‐Isopropylacrylamide (PNIPAM) as a thermoresponsive part. These bi‐functional polymeric networks successfully provide improved water absorption‐desorption kinetics even at 40°C temperature. The presence of PNIPAM also enables it to perform its AWH actions under natural sunlight [66]. Another study shows the superiority of their hygroscopic polyzwitterionic hydrogel—Tunable hygroscopic mix‐charged polyzwitterionic hydrogel (THMPH) over conventional ionic crosslinking hydrogels like PDMAPS. Novelty and tunability of their mix design make this hydrogel stable for a higher loading of LiCl, resulting in greater hygroscopic ability with rapid water absorption‐desorption rates. THMPH have shown remarkable hygroscopic rates; at 30%, 50%, 70%, and 90% RH, the rates were found to be 1.2, 2, 2.9, and 5.2 g/g, respectively, with 90% release of equilibrium moisture within just 2.5 h [58]. Apart from hygroscopic behaviour, strong ionic interaction within the backbone of the hydrogel makes it a robust one; as a result, it registered a 124.6 kPa tensile strength that is 230% higher than PDMAPS. The hydrogel was also able to regain its original shape and maintain this performance over 25 strain cycles. Using a slightly different substrate, Li et al. synthesized polyacrylamide‐based hydrogel containing CaCl_2_ and CNT, which further valorizes the fabrication of solar water collection devices. Figure 5d illustrates ionic clusters such as ‐CONH_2_, ‐COOH, ‐SO_3_^−^ aggregates around the polymer chains and form a localized ionic clusters within the matrix, which can accumulate Ca^2+^, Cl^−^ ions and water molecules through physical interactions. At an average temperature of 26°C and 60–70% RH, the device was able to collect 20 g of fresh water in just 2.5 h. Simple design, cheap materials and energy efficiency stood for efficacy and acceptability of this instrument [74].

### 2.4 Swelling–Deswelling Dynamics Under Cyclic Humidity

**FIGURE 6:**
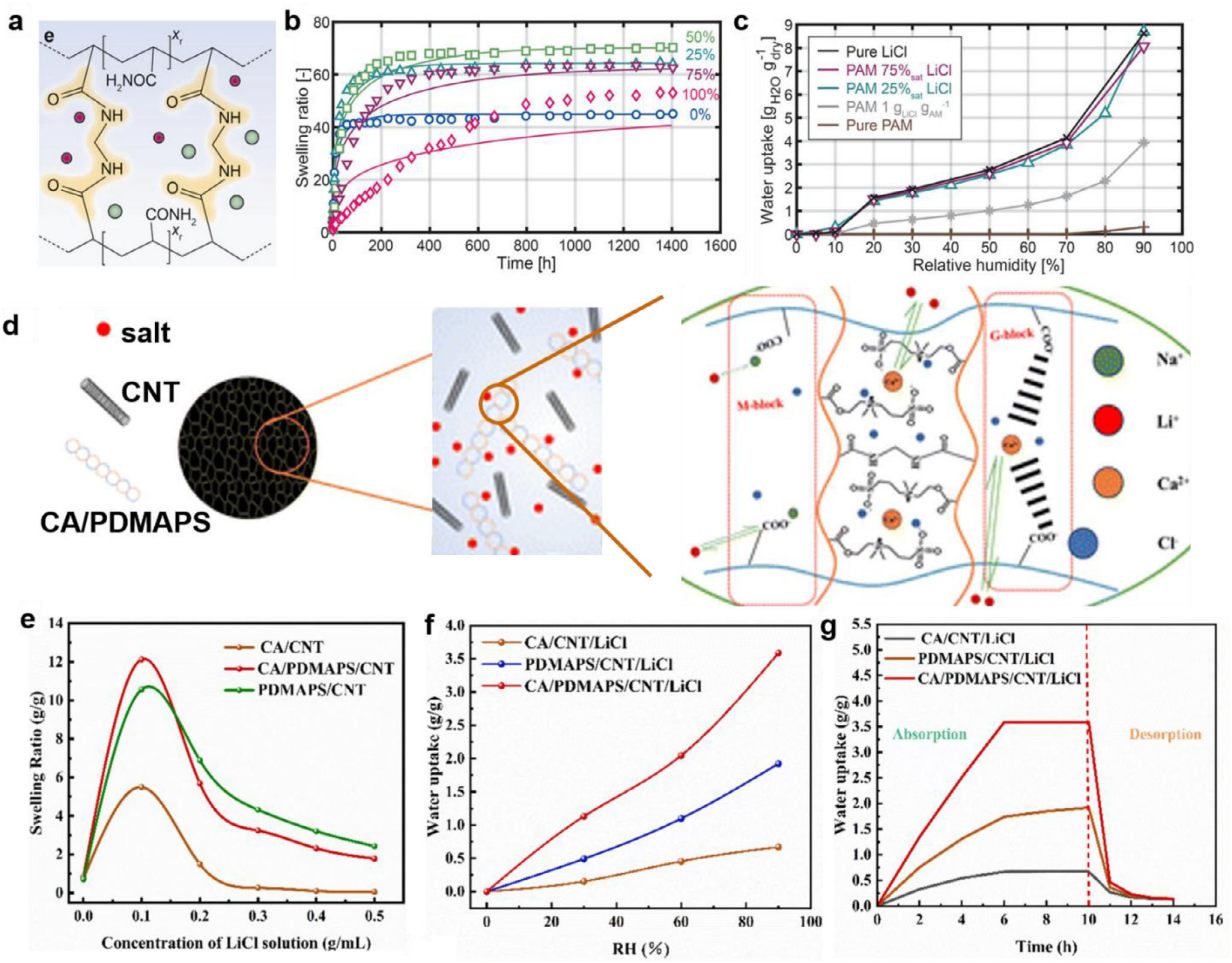
a) A simple magnified schematic representation of the hydrophilic polymeric chain connected via N, N‐methylene‐bisacrylamide (MBA) crosslinker; x represents the number of repeat units between the crosslinks; after swelling in a salt solution, a large number of ionic salts would be present inside the hydrogel matrix [41]. b) Swelling ratios of the PAM hydrogel at different concentrated solutions [41]. c) Water uptake comparison at various relative humidity of PAM hydrogel in an aqueous 75%_sat_ and 25%_sat_ LiCl solutions, sequentially alongside pure PAM, PAM 1gg^−1^ LiCl measured at 30°C; the other samples were measured at 25°C. Reproduced with permission from ref [41] Wiley, copyright 2024. d) Molecular structural diagram of CA/PDMAPS/CNT/LiCl hydrogel. e) Representing the swelling ratios of CA/CNT, CA/PDMAPS/CNT and PDMAPS/CNT hydrogels, respectively, in LiCl aqueous solution with various concentrations. f) Moisture sorption isotherms at 22°C. g) Static moisture adsorption‐desorption curves. Reproduced with permission from ref [75]. RSC, copyright 2025.

Tailoring the pore size, matrix properties, morphology, interfacial interactions, and processing methods plays a crucial role in determining the swelling‐deswelling dynamics. This is another key factor that can be well‐optimized using polymer chemistry to achieve desirable properties. Salt concentration does have a noteworthy role in varying swelling dynamics. Graeber et al. found in their study that with a 50% saturated aqueous solution of LiCl, the swelling ratio of polyacrylamide hydrogels (Figure 6a) went about 70 as compared to 45 in the case of pure deionized water. An opposite trend was encountered on further increasing the LiCl concentration; at 100% saturation, the swelling ratio was diminished to 53 (Figure 6b) [41]. Figure 6c represents that further alteration of RH at this 75% saturated solution of LiCl improved water uptake to 1.85, 2.64, and 3.94 g. g^−1^ at RH of 30%, 50%, and 70%, respectively. The fabricated material also stood out in terms of cyclic stability, with a mere 3% leakage of salt after 13 vigorous cycles of sorption at 25°C and desorption at 70°C. Aleid et al. described the “salt‐in effect” in tailoring the swelling behaviour in their zwitterionic hydrogel PDMAPS, loaded with CNT and LiCl. The salt‐in effect boosts the swelling ratio from 1.37 to 12.25 g.g^−1^, upon regulating the LiCl concentration to 0.1 g/mL from pure DI water [54]. Exactly the reverse of the salt‐in effect, it was observed that immersion of the hydrogel in pure water leads to deswelling because of the “Salt‐out effect”. However, this research also suggests that at a higher concentration of LiCl, such as 0.8 g/mL (supersaturation state), the swelling ratio approaches zero. The reasoning of the phenomenon can be delineated in such a way that when the hygroscopic salts are added into the zwitterionic polymeric matrix of the hydrogel, some fractions of the ions readily go into coordination with the ionic groups of the polymeric backbone through electrostatic interaction. Such molecular interactions promote network expansion during vapour uptake, while the remaining fraction stays within the matrix as free salt, which has much higher mobility and can be easily driven by applying osmotic pressure [57]. Chen et al. did an interesting study by preparing a double‐network structured hydrogel sphere via a novel dip‐free polymerization strategy using Ca‐alginate and PDMAPS, loaded with CNT and LiCl; the molecular structure of the hydrogel is demonstrated in Figure 6d [75]. The swelling ratio of these double‐network‐structured hydrogel microspheres was improved with increasing salt concentration (Figure 6e). Furthermore, the water collection efficiency was 434 times greater than that of usual single‐network‐structured AWH systems, as demonstrated in Figure 6f. On the other hand, these materials have also demonstrated a good water desorption capacity, desorbing more than 80% of the water within 4 h (Figure 6g) under natural light. Even after 30 continuous cycles of sorption‐desorption, the hygroscopic properties did not change slightly. They also fabricated a water harvesting device that can produce 8.5 g of fresh drinking water with just 5 g of sample within a day. Water harvesting challenges under low solar radiation were addressed by Wang et al. [76]. The design of their polyacrylamide‐poly‐NIPA hydrogel, mixed with polydopamine and sodium alginate, was optimized for high water collection at low energy input. It can switch between hydrophilic and hydrophobic modes for fast sorption and long retention of water. This hydrogel has marked an incredible AWH performance by a water sorption rate of 5.89 and 9.8 kg m^−2^ h^−1^ under 0.6 and 1 sun irradiation [76]. Among the constituents, polydopamine and sodium alginate have shown their efficacy in collecting clean water by continuously repelling the contaminants. Whereas the incorporation of acrylamide in the polymeric backbone enriches the mechanical properties and swelling ratio. After 9 cycles of compression and relaxation at 80% compression limit, the hydrogel was still able to show its shape‐recovery property. As novel as the previous two studies, Hu et al. developed another new strategy for making hygroscopic polymeric gels for AWH [77]. They have also used the usual precursors like PDMAPS and LiCl, but the uniqueness lies in their strategy. The method allows a cosolvent‐induced gelation by carefully adjusting the DMSO/water ratio; the procedure plays an effective role in generating microchannels for greater vapour diffusion while maintaining the structural integrity over multiple sorption‐desorption cycles. These entanglement‐enhanced sponge hydrogels could collect water at a 0.95‐1.48 g g^−1^ h^−1^ rate across a wide range of RH of 20–90%. Especially at 30% RH, the hydrogel performed exceptionally well, with a rapid desorption rate of 7.57 g g^−1^ h^−1^ and over 97.6% shape recovery after 30 cycles. Even outdoors, this hydrogel has produced fresh water with a rate of 4.15 L kg^−1^ day^−1^ [77].However, translating these molecular‐level interactions into practical water harvesting performance requires a structural configuration of the hydrogel network across the nano to macro level. Cyclic swelling‐deswelling determines the dynamic storage of water within the matrix, while precise control over this behaviour requires careful tuning of the polymer network. This can be done by using a copolymerized hydrogel. Therefore, designing a graft or block copolymerized architecture can be explored to identify the strategic distribution of functional groups and regulate moisture sorption.

### 2.5 Copolymerization and Architectures for Controlled Sorption

At times, a single‐component hydrogel can rarely achieve all the desired characteristics, such as mechanical strength, fatigue resistance, swelling capacity, self‐healing properties, biocompatibility, and stimulus responsiveness. Consequently, strategic copolymerization is employed for decoupling and redistribution of the roles across distinct polymer segments. For example, Hydrogels based solely on homopolymers, such as polyacrylamide or polyacrylic acid, often exhibit several drawbacks, including extremely high or low swelling ratios, decreased moisture sorption capacity, slow kinetics, and excessive brittleness [78, 79, 80]. But a hydrogel composed of both polymers with an optimized crosslinking ratio, with multilevel physical bonding, shows enhanced swelling capacity while providing mechanical integrity, making this hydrogel more suitable for moisture adsorption and water harvesting applications [81, 82]. Building on this concept, various types of copolymers were used to develop hydrogels, such as block copolymers, grafted copolymers, terpolymer‐based systems, etc. Similar to the choice of polymer for making hydrogels, engineering of the hydrogel's composition is equally important; the percentage of the used monomer can tune the hydrogel's properties according to application [83]. Scientists therefore shifted their focus to designing advanced copolymer architectures that could outperform conventional homopolymeric hydrogels by facilitating faster sorption kinetics, improved mechanical stability, and higher sorption capacity. Here, we have used some references in support of this topic.

**FIGURE 7:**
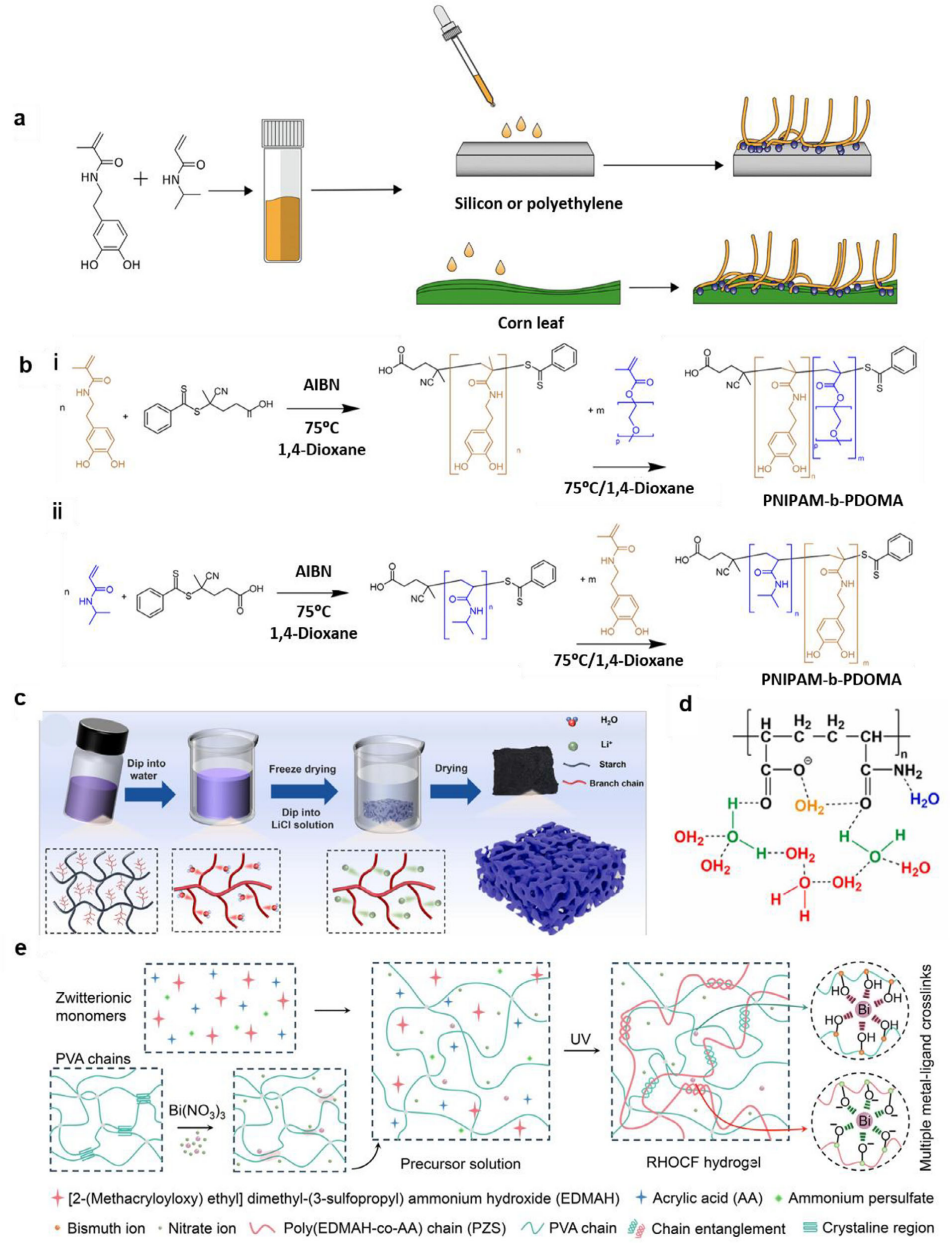
a) Schematic presentation of the synthesis of block copolymer PNIPAM‐b‐PDOMA, silicone or polyethene surface and leaf coating by using catechol as an anchoring site. b) Schematic illustration of block‐copolymerization (i) PNIPAM‐b‐PDOMA (ii) sequential RAFT polymerization of PNIPAM‐b‐PDOMA. Reproduced with permission from ref [85]. RSC, copyright 2025. c) Step‐wise graft copolymerization of AA/AM‐based hydrogel. d) Schematic illustration of water adsorption through hydrogen bonding. Reproduced with permission from ref [86]. Elsevier, copyright 2023. e) Schematic representation of hydrogel formation via copolymerization of Poly (EDMAH‐co‐AA) and PVA chain. Reproduced with permission from ref [65]. Nature, copyright 2025.

Konjac glucomannan (KGM) is a biocompatible material and provides the porous structural backbone of the hydrogel, but fails to provide mechanical integrity, thermoresponsiveness and water desorption at lower temperatures. The incorporation of a thermoresponsive material, hydroxypropyl cellulose (HPC), helps in this matter. The combination of these 2 specific polymers helps in continuing more than 20 cycles per day, while maintaining structural integrity [3]. Similar to this, poly([2‐(acryloyloxy)ethyl] trimethylammonium chloride) (PAEtMA) enables strong adsorption through ionic crosslinking, but fails to provide mechanical properties and thermal responsiveness. Incorporation of PNIPAM and polydopamine‐manganese (II) PDA‐Mn nanoparticles exhibits thermo‐photo‐responsive behaviour and mechanical robustness [84]. Milatz et al. designed a hydrogel through block‐co‐polymerization of two different polymers to fulfil distinct purposes; a demonstration of the synthesis process is shown in Figure 7b. Here, the copolymerization of a thermo‐responsive polymer, PNIPAM and poly(dopamine methacrylamide) (PDOMA), is needed to be used as a hydrogel coating of plants without interrupting photosynthesis (Figure 7a).

A single homopolymer alone is insufficient to fulfil the requirement of low‐temperature desorption and to form strong adhesion to the coated substance [85]. Additionally, the other important properties required for a hydrogel, including surface roughness, polyelectrolyte behavior, uniform or hierarchical pore structure, ordered patterning, and crosslinking density, can all be tailored through crosslinking of two or more polymers [66, 87, 88, 89]. Based on this concept, Guan et al., Zhang et al., Feng et al. focused on the copolymerization of hydrogel. Copolymerization a polyelectrolyte polymer with a non‐electrolytic polymer result in the prevention of salt crystallization, salting‐out and excessive bound water formation. For the copolymerization of a zwitterionic polymer, PDMAPS with PNIPAM the non‐electrolyte unit provides thermally responsive conformational changes and water desorption at a very low temperature, while the ionic segments electrostatically bound the ionic salts [66]. In the case of graft copolymerization of acrylic acid (AA) and acrylamide (AM) (Figure 7c) onto a starch backbone, the AA units introduce ionizable carboxylate units and electrostatic coordination, which induced osmotic swelling, while AM units promote hydrogen bonding with water molecules (Figure 7d), resulting in improved mechanical strength and network stability. This is an important strategy to overcome the inherent limitations of starch; despite being a biocompatible scaffold, it lacks enough hygroscopicity and resilience. In contrast, grafting provides an increased surface area and creates an expanded, more accessible sorption interface, thereby significantly improving water‐harvesting performance [86]. Homopolymeric systems based on Methacrylic acid (MAA) or poly(ethylene glycol) methyl methacrylate (PEGMA), exhibit complementary limitations: the former exhibits high hygroscopicity but poor desorption efficiency, whereas the latter enables good flexibility and thermoresponsiveness but limited uptake of it has been established that copolymerization of these two particular hydrogels integrates higher hygroscopicity with temperature responsiveness within a single network while maintaining high mechanical stability and durable cyclic performance through optimized and controlled crosslinking [90]. Though inclusion of zwitterionic polymers removes bottlenecks related to salt, along with it come some other drawbacks due to the presence of ionic functional groups, which induce the aggregation of polymer chains and lead to poor flexibility and mechanical stiffness. But the addition of functional groups through the copolymerization (Figure 7e) of poly([2 (methacryloyloxy) ethyl] dimethyl‐(3‐sulfopropyl) ammonium hydroxide (EDMAH) and Acrylic acid (AA) suppresses self‐aggregation and increases network homogeneity. Introduction of flexible polyvinyl alcohol (PVA) chains with a copolymeric system enables effective physical entanglements, resulting in improved stress transfer and toughness. This copolymer demonstrates superior mechanical properties over the pure poly zwitterionic salt (PZS) system [65]. Wang et al. copolymerized the hydrophilic polymer Poly(sodium acrylate) (PAAS) and thermoresponsive polymer, Poly(N‐isopropylacrylamide) (PNIPAm), photothermal agent Polyaniline (PANI), while mimicking the hierarchical hydrophilic/hydrophobic surface pattern of the skin of Pachydactylus rangei, found in the Namibian desert, resulting in an enhanced water uptake through the increased roughness on the pore wall surfaces, efficient desorption under heating, zero salt aggregation and structural integrity [87].

### 2.6 Benchmarking Hydrogels Against MOFs, Salts, and Porous Carbons

Regeneration energy and cycling durability are critical parameters for evaluating hydrogels along within established sorbent classes such as MOFs, hygroscopic salts and porous carbons, because their intrinsic chemistry governs water uptake capacity, sorption kinetics and regeneration behaviour.

Hydrogels, MOFs, metal‐organic complexes, salts and porous carbons all harvest water from the atmosphere, but all of these have different sorption mechanisms. In the case of Hydrogels, polymers containing hydrophilic functional groups, such as, carboxyl, hydroxyl, amide and sulfonate groups capable of forming strong secondary interaction with adsorbed water. This adsorbed moisture helps in swelling of the hydrogel and increase network size. This enhanced network structure leads to enlargement of the pore size, which allows the continuous diffusion of water into the internal network of the gel. Owing to this hierarchical(nano to macro) porous structure where initial surface interactions convert into volumetric swelling and give hydrogels that are characteristically high‐water content and low regeneration temperature, often below 60°C [50, 91]. Conversely, in the case of MOFs, it absorbs moisture through pore filling process, which is dominated by its crystalline micro‐porous structure. Their adsorption isotherms frequently show well‐defined, sharp relative humidity values due to the cooperative hydrogen‐bonding matrix within the porous structure as n in MOF‐801, MOF‐303, and CAU‐10‐H [1, 5]. These properties of MOFs permit surpassing at low‐RH which is important for arid‐region AWH, but the strong host‐guest interactions typically require high desorption temperatures in the range of 60°C–90°C.

In the case of hygroscopic salts like LiCl, MgCl_2_, CaCl_2_ etc., employed via a totally different mechanism, which is called deliquescence. When the crystalline salt is allowed to dissolve into an aqueous medium, ions bind to the Water molecules, resulting in exceptionally high gravimetric uptake values that can exceed 300 wt% [74]. While this capacity is unmatched, the absorption process produces liquid water, which changes encapsulation and substantially raises regeneration energy because liquid water must be evaporated to restore the sorbent. On the other hand, porous carbons rely mainly on weak physisorption through Vander Waals forces, and in the case of functionalized this occurs through limited hydrogen bonding. Their overall uptake is lower compared to hydrogels or MOFs, especially at low RH, but their structural robustness, chemical stability and ease of large‐scale production make them attractive for applications where durability outweighs capacity.

When these materials are compared, key performance dimensions, such as strengths and limitations, become more evident. Due to the open microporous matrix, MOFs show excellent low‐RH absorption and adsorption kinetics on the order of minutes; conversely, hydrogels offer medium water uptake combined with exceptionally low regeneration energy and also provide operational safety. For the case of Sali‐based systems, they can achieve the maximum theoretical capacities but at the cost of leakage risk, corrosivity and high desorption energy. Porous carbons exhibit comparatively lower water uptake (< 50 wt.%) but achieve 90% of equilibrium adsorption within 40 min at 25°C, in 50% RH, moderate performance but surpass in cycling stability and mechanical elasticity. These differentiations underline that while MOFs and salts may outperform hydrogels in terms of gravimetric capacity under certain humidity profiles, hydrogels surpass all three classes in energy‐efficient regeneration and versatility of chemical design. Indeed, recent analyses emphasize that hydrogels show some of the most favourable energy‐per‐litre‐of‐water figures among AWH materials because water is stored in weakly bound, easily releasable states [92].

Besides MOFs, porous carbon, hygroscopic salts and hydrogels, metal‐organic coordination complexes have emerged as a promising class of AWH sorbents. An amorphous metal‐organic complex, Cu(II)—ethanolamine, achieved 3.05 g g^−1^ at 95% RH and undergoes regeneration at 60°C, under solar irradiation. These characteristics highlight its potential applicability in agricultural fields. Hydration and dehydration can occur due to the reversible secondary interactions, strong electrostatic attraction between atmospheric moisture and ionic Cu^2+,^ as well as hydrogen bonding with ethanolamine ligands. This material stands out because of its budget‐friendly synthesis process, high water uptake and yield, speedy sorption kinetics (∼ 95% water desorption within 10 min with inclusion of photothermal material, CNT) and excellent stability over 60 hydration‐dehydration cycles, making it an attractive alternative to expensive MOFs [93]. The key performance characteristics of the major solid AWH sorbents are summarized in Table 4, which compares water uptake capacity, sorption kinetics, regeneration conditions and cyclic stability. These mechanistic and performance differences confirm the need for hydrogels when evaluating using a specialized performance matrix that is distinct from the metrics historically applied to MOFs or porous solids. Their performance cannot be fully captured by conventional metrics such as pore‐filling capacity or adsorption step position since hydrogel function depends not only on chemical affinity but also on polymer chain mobility, elastic modulus, swelling ratio and water transport through a hydrated network. Instead, classification must consider dynamic parameters such as diffusion‐controlled uptake, swelling‐deswelling hysteresis, mechanical fatigue under repeated cycles and the interplay between cross‐linking density and water release thresholds.

**TABLE 4:** Performance comparison of major AWH sorbents.

Sorbent class	Water uptake capacity	Sorption Kinetics	Regeneration condition
Polymeric hydrogels [23, 66]	100 wt.% at 60% RH	1 g g ^−1^, water adsorption within ∼100 min, 80% saturation within 30 min, > 80% water release within 20 min at 40°C	36°C–41.5°C
Hydrogel‐MOF [94]	64‐276 wt.%	Water uptake 1.827 g g^−1^ at 298 K, RH = 90%.	≤ 60°C
MOF [95]	∼30‐40 wt.%	Adsorption 1–2 min, desorption < 10 min at 85°C, has potential ≥ 1000 cycles, water yield – 1.3 L_water_kg^−1^ of MOF/day.	45°C‐85°C
Hygroscopic salt‐based composites (hydrogels, textile composites) [26, 96]	>300 wt.%	1.91 – 4.8 g g^−1^ water adsorption in 12 h at (70%–90% RH) at 25°‐30°C, water desorption > 80% in 20 min, (> 20 cycles), daily yield 64 g day^−1^	70°C‐90°C
Porous carbon [72, 97]	<50 wt.%	90% water adsorption in 40 min @25°C, RH = 50% Under one sun >99% of water desorption in 10 min	50°C‐62°C
Metal‐organic complex [93]	∼305 wt.% (3.05 g g^−1^) at 95% RH)	Water yield of 2.24 g g^−1^ h^−1^ under one solar irradiation, 95% desorption within 10 min	∼ 60°C under one sun irradiation

Despite their low regeneration energy and tunable polymer chemistry, hydrogel‐based sorbents still face several limitations that hinder their widespread deployment for atmospheric water harvesting. Compared with MOFs and highly hygroscopic salts, most hydrogels exhibit relatively lower water uptake under extremely arid conditions (<10%–20% RH) because moisture capture relies on polymer hydration and network swelling rather than micropore filling or deliquescence [3, 98]. In addition, repeated swelling–deswelling cycles can induce network fatigue, pore collapse, and gradual loss of mechanical integrity, while salt‐loaded hydrogels may suffer from salt migration or leakage, reducing cyclic stability. Although recent zwitterionic, double‐network, and interpenetrating polymer architectures have significantly improved durability and salt retention, most systems have only been evaluated over limited adsorption‐desorption cycles. Therefore, improving ultra‐low‐humidity water harvesting, long‐term mechanical durability, and effective salt immobilization remains a major challenge for the practical implementation of next‐generation hydrogel‐based atmospheric water harvesting materials.

### 2.7 Stimuli‐Responsive Hydrogels for Water Release

#### 2.7.1 Thermally and Photothermally Stimulated Hydrogels

Optimizing desorption enthalpy to enable regeneration using solar or other easily available low‐grade energy sources is crucial for water harvesting. Therefore, scientists introduced two primary approaches that can be helpful in achieving this goal: one is a thermoresponsive material, and the other is a photothermal reagent [99, 100]. Upon exceeding the Lower critical solution temperature (LCST), thermoresponsive polymers sharply change their solubility by switching their structural conformation from hydrophilic to hydrophobic. Below the critical temperature, the material adsorbs water, but when the temperature gets closer to the LCST, the material shrinks, thus the captured water is desorbed. Photothermal materials adsorb light energy and convert it into localized thermal energy, facilitating the disruption of physical bonding between water and thermoresponsive hydrophilic functional groups, desorption of water begins. Both of these materials are helpful in minimizing energy requirements and ensuring a smooth sorption cycle. Photothermal fillers such as polypyrrole (PPy), graphene oxide, Detonation nanodiamond (DND), etc. enhance desorption kinetics by heat localization under solar irradiation [101, 102].

**FIGURE 8:**
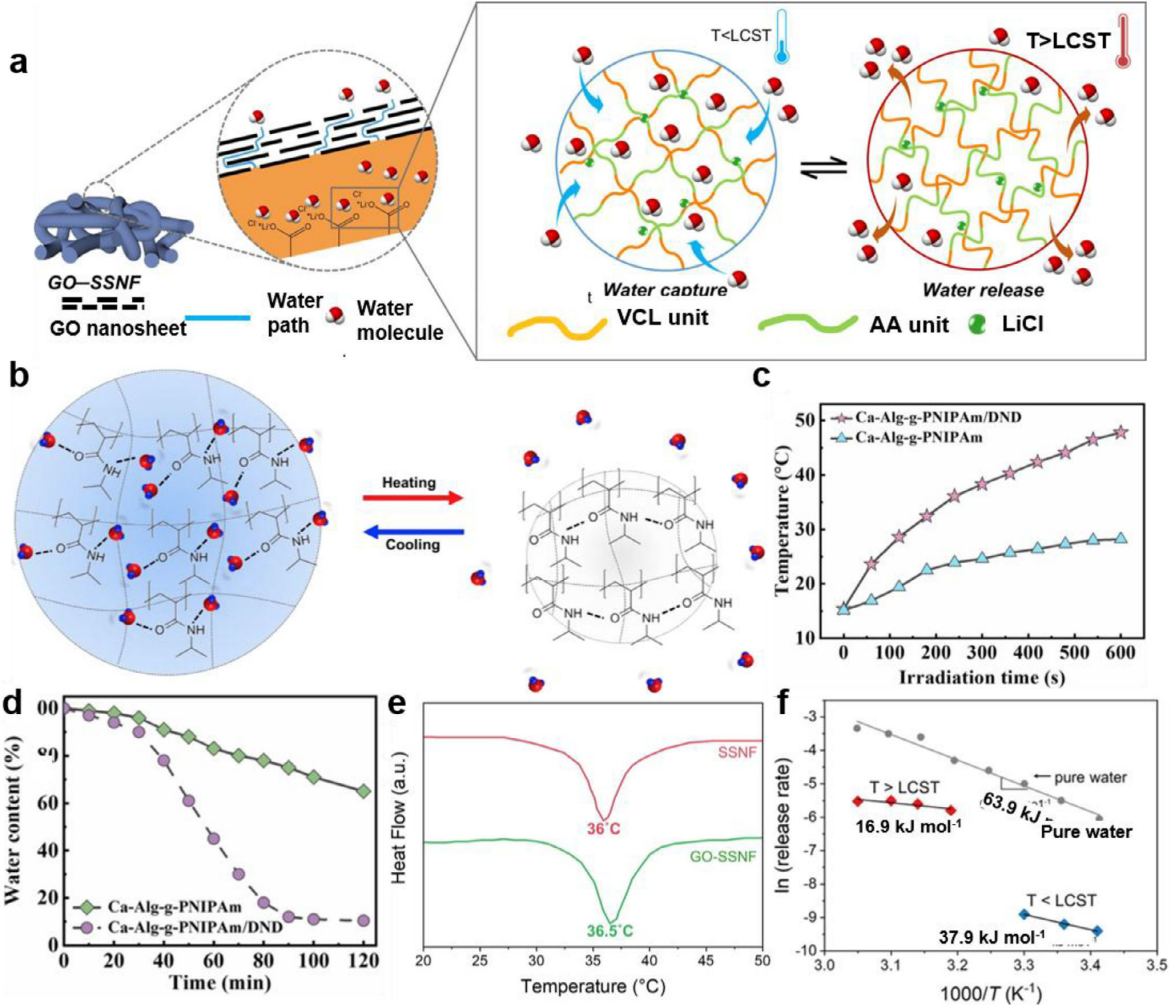
a) Schematic representation of the GO‐SSNF structure, photothermal and thermoresponsive effect of GO and PVCL. Reproduced with permission from ref [104]. Elsevier, copyright 2021. b) LCST‐driven molecular transition changes of PNIPAM with increasing temperature. Reproduced with permission from ref [106]. Elsevier, copyright 2016. c) Increasing of temperature due to the photothermal effect of DND. d) Abrupt change in the rate of water content (%) owing to the photothermal effect of DND. Reproduced with permission from ref [101]. Elsevier, copyright 2023. e) DSC thermograms depicting the LCST transitions of SSNF and GO‐SSNF. f) Demonstration of the release of water (●) and GO–SSNF (◆) with change of temperature through Arrhenius plot. Reproduced with permission from ref [104]. Elsevier, copyright 2021.

An important milestone in this topic of introduction of thermoresponsive hydrogel was established by Guo et al., who designed a biodegradable hydrogel by composing porous Konjac glucomannan and thermoresponsive hydroxypropyl cellulose (HPC) for water harvesting in arid regions. The microporous structure facilitates enlarged interracial area between hydrogel sorbent and environment, results in a rapid sorption kinetics. The thermoresponsive HPC transforms into hydrophobic phase by the bond movement of hydroxypropyl group by crossing the temperature 45°C. The secondary interaction between HPC and KGM forms a mechanically stable network without any chemical crosslinking. This super hygroscopic polymeric films facilitate 24 cycles per day with a high‐water yield of 13.3 Lkg^−1^ at 30% RH [3]. Another landmark advancement in water harvesting technology was introduced by Zhao et al. the interpenetration of a thermoresponsive and photoresponsive polymer, PNIPAM and Cl‐doped PPy, respectively. Dynamic mechanical analysis of this super moisture‐absorbent gel (SMAG) reveals that the values of both storage (***G′***) loss modulus (***G′′***) are lower than those of the pristine thermally stimulated polymer. It indicates a successful interpenetration of PPy‐Cl. The higher value of storage modulus confirms the formation of a stable crosslinked network. Due to the presence of positively charged nitrogen and chloride ion PPy‐Cl uptakes moisture from the environment over a broad range of humidity (30%–90%) through electrostatic interaction, while the PNIPAM network stores the adsorbed water and rapidly releases the water upon increasing the temperature above LCST by altering their phase from hydrophilic to hydrophobic. This combination of different types of stimuli‐responsive materials, the water harvesting capacity at 90%, 60% and 30% is 6.7, 3.4 and 0.7 g.g^−1^ respectively. Synthesis of an interpenetrating network containing both thermoresponsive and photoresponsive polymers demonstrates an effective strategy to generate a mechanically robust hydrogel which maintains the structural integrity and weight after performing over 100 swelling‐deswelling cycles, while maintaining a constant water harvesting over a wide range of relative humidity [103].An interconnected honeycomb‐like structure, composed of carbon black within a melamine foam crosslinked with sodium alginate and LiCl, facilitates a solar‐triggered desorption rate of 1.90 kg·m^−2^·h^−1^ and produces water at a yield of 2.84 kg·m^−2^·day^−1^. Localized photothermal heating of CNT at the evaporation interface drives desorption of water from the salt solution while keeping the polymer backbone structurally stable [83]. The photothermal nature of graphene oxide converts adsorbed solar energy into thermal energy, increasing the surface temperature of the hydrogel to facilitate water release (Figure 8a). Differential scanning calorimetry (DSC) shows an endothermic peak around 36°C, which is the LCST of the copolymer, VCL/AA. Upon exceeding the LCST of the copolymer, the hydrogen bonding between the hydrophilic functional groups of the copolymer and water molecules was broken. The inclusion of a hydrophilic photo‐responsive material, GO, marginally increases the overall hydrophilicity of the system and the LCST to 36.5°C (Figure 8e) [104]. Below the LCST, the desorption energy of water is 37.9 kJ mol^−1^ (Figure 8f), much higher than after the LCST, when it drops to 12.13 kJ mol^−1^, providing clear evidence of a significant reduction in desorption enthalpy [104]. From Lu et al.’s research, it can be established that the introduction of a photothermal and solar‐assisted material, CNTs in a thermoresponsive (PNIPAM) hydrogel, resists shrinkage and increases mechanical stability. This system can collect 1471 g of water per kilogram of sorbent over nine cycles per day [105]. A composite sorbent by copolymerizing poly(N‐isopropylacrylamide) (PNIPAM), which is a thermoresponsive material (Figure 8b), with the photothermal polymer polypyrrole (PPy) [106]. The composite exhibits its maximum particle size at 28°C; as the temperature starts increasing toward 32°C, due to the photothermal behaviour of PPy, the polymer matrix shrinks due to water desorption as the thermoresponsive polymer changes its phase [102]. A chemically crosslinked PNIPAM‐PVA network, integrated with a polyamide interfacial layer, was designed to function as a salt denial barrier. Incorporation of graphene as a photo‐thermal agent improves the thermal conductivity and solar absorption, which accelerates the desorption of water just above 40°C. The hydrophobic contraction triggers rapid water expulsion, achieving an ultrahigh water yield of 29.99 kg·m^−2^·h^−1^ [107]. A core‐shell structured hydrogel with hierarchical pores composed of thermoresponsive polymer, PNIPAAm, with sodium polyacrylate(PAAS) and LiCl, coated with polydopamine for photothermal conversion. The coating absorbs sunlight, the system gets heated and triggers the PNIPAAm to switch the phase at around the temperature of 35–40°C; water is transported from the core to the shell during this process, enabling photothermal evaporation with durability over multiple cycles [108]. Single‐component thermoresponsive hydrogels often exhibit limited sorption efficiency or mechanical robustness, therefore, systematic modification by incorporating other materials through chemical crosslinking or interpenetration is necessary to achieve the desired results. Chemical crosslinking of PNIPAM, with N, N‐methylene‐bisacrylamide (MBA) or incorporation of NIPAM into an interpenetrating copolymeric network structure, has improved its overall sorption performance and structural integrity [109, 110]. Thermoresponsive and photothermal agents that are generally used as components of hydrogels for water harvesting is enlisted in Table 5. Literature indicates that optical and thermodynamic methods, especially differential scanning calorimetry, are most commonly used for characterizing phase transitions for thermoresponsive hydrogels, specifically PNIPAm‐based hydrogels. Reports consistently show that the volume transitions occur near the LCST, although the exact transition depends on the composition, crosslinking density and network architecture of the hydrogel. Additionally, the swelling‐deswelling dynamics not only depend on exceeding or lowering the temperature beyond the LCST but also require a minimum energy to overcome the water desorption barrier [110]. Notably, a sharp temperature rise, higher water release percentage (Figure 8d) [101] (DND/Ca‐Alg‐g‐PNIPAm the water yield is ∼ 90%, while for Ca‐Alg‐g‐PNIPAm it is ∼35%), and lowering of desorption enthalpy can be noticed only after incorporation of a photothermal agent, such as the inclusion of DND and GO in the PNIPAM (Figure 8c) [101] and N‐vinyl caprolactam (VCL)/AA copolymer (Figure 8f) [104]. A molecular‐level modification of a polysaccharide backbone via grafting of thermoresponsive alkyl side groups, such as hydroxypropyl or hydroxybutyl, disrupts the dense inter‐ and intramolecular hydrogen‐bonded network structure, which limits the pore expansion and water sorption capacity. These thermoresponsive groups also enable low‐temperature water desorption [40]. Though the inclusion of thermoresponsive polymers with photothermal materials significantly reduces the energy barrier and enables efficient solar‐assisted regeneration. However, complete dependence on solar or photothermal stimuli can limit the operational efficiency under fluctuating environmental conditions and at nighttime or in a cloudy environment. Consequently, some other alternative external stimuli are needed, which should be capable of triggering the sorption and desorption process. Electric and magnetic‐based stimuli have attracted increasing attention.

**TABLE 5:** Thermoresponsive and photothermal components used for atmospheric water harvesting.

Stimuli‐responsive materials and characteristics	Material	Performance
Carbon black—Photothermal agent [83]	Melamine foam/sodium alginate/LiCl	Photothermal conversion efficiency ∼ 85% Evaporation rate 1.90 kg m^−2^ h^−1^ Water yield 2.84 kg m^−2^ d^−1^
Graphene oxide—photothermal agent,Vinyl caprolactam and acrylic acid ‐Thermoresponsive reagent [104]	poly (N‐vinylcaprolactam‐co‐acrylic acid) (VCL‐AA)/LiCl	light absorbance (>80%) 2.61 g g^−1^ in 300 min at RH 80%
hydroxypropyl cellulose (HPC)—Thermoresponsive agent [3]	Konjac glucomannan (KGM) and hydroxypropyl cellulose (HPC) polymer matrix/LiCl	water yield ∼ 13.3 L kg^−1^ day^−1^ at 30% RH
poly(N‐isopropylacrylamide) (PNIPAM)—thermoresponsive agent carbon nanotube (CNTs)—photothermal agent [105]	Poly(N‐isopropylacrylamide) (PNIPAM)/TEMPO‐oxidized cellulose nanofibers (TOCN)/LiCl	5.43±0.37 g_water_ g^−1^_sorbent_ Water collection rate 1417 g_water_ kg_sorbent_^−1^ day^−1^
poly(N‐isopropylacrylamide) (PNIPAM)—Thermoresponsive agent Polypyrrole (PPy)—Photothermal agent [102]	Pollen‐LiCl @poly(N‐isopropylacrylamid) (PNIPAM)/polypyrrole (PPy)‐ copolymer	Water yield ∼ 2.21g_water_ g_sorbent_^−1^ day^−1^ Retain ∼96% of initial sorption capacity
poly(n‐isopropyl acrylamide) PNIPAM—Thermoresponsive agent Graphene—Photothermal agent [107]	PNIPAm /PVA	Ultrahigh water yield 29.99 kg m^−2^ h^−1^
PNIPAAm—Thermoresponsive agent Polydopamine‐ Photothermal component [108]	sodium polyacrylate (PAAS)/ polyN‐isopropylacrylamide (PNIPAAm)/carboxymethyl cellulose (CMC)/ polydopamine	Desorption capacity 2.76 g g^−1^ (90% RH) Outdoor yield 1.31 g g^−1^ day^−1^
poly(n‐isopropyl acrylamide) (PNIPAM)—Thermoresponsive agent chloride‐doped polypyrrole (PPy‐Cl)‐ Photothermal component [110]	chloride‐doped polypyrrole (PPy‐Cl) and hygroscopic poly(N isopropylacrylamide) (poly‐NIPAM)	Water uptake capacity 6.7 g g^−1^ at 90% RH
Detonation nanodiamond (DND)—Photothermal material PNIPAm—Thermosensitive component [101]	Sodium alginate/‐poly(N‐isopropyl acrylamide) (Alg‐g‐PNIPAm)	Water collection capacity ∼1.64 gg^−1^ per cycle 89.6% water desorption under 1 sun in 2 h
Hydroxypropyl cellulose / hydroxybutyl starch ‐Thermoresponsive moiety [40]	Alkyl‐modified polysaccharide (cellulose, starch, chitosan)	95% of adsorbed water desorption at 60°C, Water uptake of 2.18 g g^−1^ at 60% RH

Though thermoresponsive and photothermal hydrogels facilitate a promising strategy for sorption‐based atmospheric water harvesting to address the freshwater scarcity across the world, even in highly arid (∼ 15% RH) environments, their practical application is constrained due to several inherent trade‐offs [111]. Maximization of swelling capacity often requires the incorporation of highly hydrophilic or ionic functional groups together with a hydrophilic ionic salt. However, the presence of strong ion‐dipolar interactions often requires a higher desorption enthalpy, and merely exceeding the LCST of a thermoresponsive polymer is insufficient to achieve efficient water desorption [112, 113, 114]. Sufficient desorption (≥ 70%) still relies on active heating or the presence of an external thermal source. Crossing the LCST initiates desorption, but the yield is insufficient, and the desorption rate is relatively sluggish. So, to overcome barriers like energy requirements, sluggish kinetics and high uptake of water, the complex construction formation, such as interpenetrating networks, inclusion of MOF, photothermal materials or some other sophisticated strategic fabrication strategy is needed, which inevitably increases the material complexity and overall expenses [115]. Similarly, for the case of photothermal‐based hydrogels under lower solar irradiation, the system requires substantial thermal energy to overcome the latent heat of vaporization; this creates a trade‐off between desorption efficiency and energy consumption [116]. Furthermore, enhancement of water adsorption can only happen with proper multifunctional chemical composition, such as incorporation of hygroscopic salts at optimized concentration, or multifunctional additives. Although these multicomponent system‐based architectures significantly improve performance but also increase the mechanical complexity, difficulty in recyclability, material cost and industry‐scale applications [111, 112].

#### 2.7.2 Electric and Magnetic Field‐responsive Gels

**FIGURE 9:**
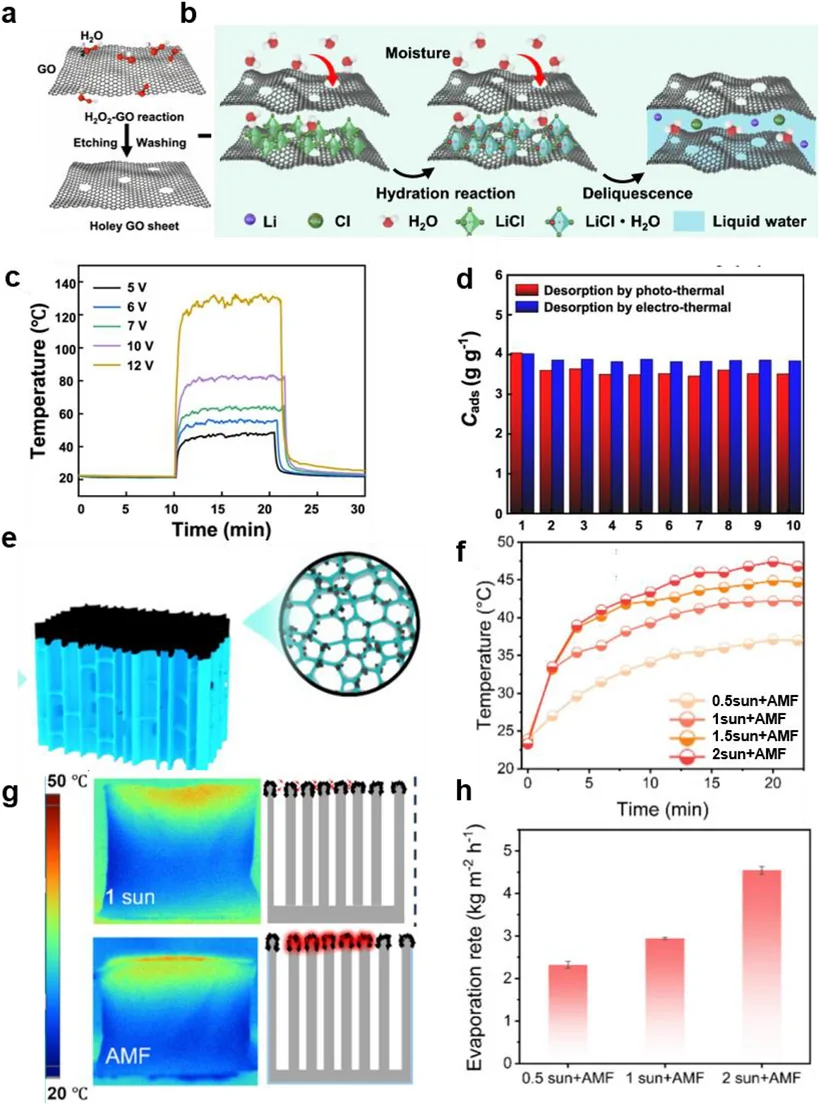
a) Schematic representation of the stepwise fabrication of hygroscopic holey graphene aerogel b) Schematic representation of the vapour adsorption process. c) Change in temperature by the application of different voltages. d) Cyclic stability of hydrogel under continuous photo‐thermal or electro‐thermal stimulated sorption process. Reproduced with permission from ref [24]. Nature, copyright 2022. e) Structure of the hydrogel composed of acrylamide and Fe_3_O_4_, top view of the hydrogel. f) Temperature rise curve of the hydrogel under the photothermal and magnetothermal stimuli g) Side view infrared image of the hydrogel under 1 sun illumination and alternating magnetic field h) Coupled evaporation rate of alternating magnetic field and photothermal heating. Reproduced with permission from ref [23]. Nature, copyright 2025.

Moisture‐enabled electricity generation (MEG) has gained significant interest as a sustainable energy approach. Though conventional MEG materials suffer from several drawbacks, such as insufficient material‐moisture interaction at lower humidity, limited water and moisture storage capacity, rapid lowering of ionic gradient at higher humidity, etc., hydrogels offer a promising solution due to their superior hydrophilicity, higher water or moisture uptake capacity, biocompatibility, easy diffusivity of mass and temperature as well as ionic conductivity. Polyelectrolyte‐based hydrogels exhibit swelling and deswelling under the application of an external electric field. Lu et al. made a unique hydrogel by integrating two oppositely polarized ions, an anionic‐cationic heterostructure, which is effective for electricity generation and fresh water harvesting. The polyanionic component was made of poly(sodium 4‐styrene sulfonate) (PAAS), and the polycationic component was 3‐acrylamidopropyl [trimethylammonium] chloride (DMAPAA‐Q). These two inversely charged hydrogels were coupled by a PA/PC heterojunction. The adsorbed water dissociates the counterions from the polymeric chains; these released oppositely charged ions enable moisture‐induced electricity generation. The adsorbed water is mostly in a loosely bounded state around the diffused ions. In this system, a moisture‐governed ionic gradient results in the generation of electricity while the CNT is absorbing the sunlight and converting it into heat; this heat allows the hydrogel to release the adsorbed water under solar irradiation. After twenty minutes of irradiation under the sun, a mass loss of 50% can be achieved. And after the sun exposure of half an hour 95% of the water can be effectively desorbed from the device [73]. Another graphene‐based aerogel composed of etched graphene oxide with H_2_O_2_, to generate a highly porous, interconnected network, followed by loading of LiCl, which enhances hygroscopicity, vapour diffusion (Figure 9a,b). The electrical conductivity of the aerogel is 149 S m^−1^. Because of the hygroscopic nature of LiCl and as it is combined with a large specific surface area and interconnected porous structure of the graphene network, the aerogel obtained an ultra‐high water sorption capacity from a wide range of relative humidity (30%‐90%). The water sorption capacity of LiCl@HGAF is more than 4.15 g g^−1^. Apart from photothermal conversion, LiCl@HGAF shows electro‐thermal capability. So, both solar energy and electrical energy can be used as the driving force of desorption. As the aerogel has a conductive network structure, it shows an excellent electrothermal desorption of captured water upon application of 12 V, and the temperature rises to 131°C (Figure 9c). However, the network manages to undergo 10 continuous sorption cycles without any obvious change in the network structure (Figure 9d) [24]. Solar energy is a well‐known, renewable and clean source of energy. Photothermal materials in hydrogels can convert solar energy to thermal energy, and moisture desorption happens. But it is not ideal under cloudy or rainy weather or at night. So, for another alternate magnetic induced heating can be used, here, an eddy current is generated by the application of an electromagnetic field to heat the device. This induced eddy currents dissipate energy inside the conductor and transform it into heat. Magnetic induction heating can uniformly heat the interior of the material, and its efficiency is not limited by the depth of the material. But in contrast, photothermal heating is limited only to the surface region, because light cannot penetrate to the depth of the material, which is not varied by the thickness of the material; hence, only the upper region of the material is effectively heated, while the lower region remains unaffected. Although some work had previously been done on magnetothermal heating, there were challenges in creating a stable, fixable magnetic material on the evaporator surface without hindering the continuous water supply. Additionally, the interfacial adhesion between the magnetic particles and the matrix material was insufficient, characterized by weak physical bonding that could easily break down under operating conditions. To overcome these challenges, Du et al., Lin et al. introduced magneto‐responsive hydrogel composed of iron oxide, such as magnetite, and super‐hydrophilic acrylamide polymer as the matrix [23, 117]. As shown in Figure 9e, the channels generate strong capillary forces, which surely transport water molecules to the upper Fe_3_O_4_ evaporation surface. Besides exhibiting a ferromagnetic behaviour, Fe_3_O_4_ also demonstrates excellent photothermal conversion properties. Under 2 sun or 2 sun with AMF, the temperature rises to 43°C and 47°C, as shown in Figure 8f, and the evaporation rate reaches 2.94 kg m^−^^2^ h^−^^1^ and 4.54 kg m^−^^2^ h^−^^1^ (Figure 9h), respectively. In this system, the alternating magnetic field (AMF) and sunlight act synergistically to generate heat within the hydrogel. In daytime, both solar and magnetic heating can be used, whereas at night or in cloudy weather, the magnetothermal process alone remains effective [23]. Correspondingly, here is another example of magnetic induction heating technology under an alternating magnetic field to achieve uniform and rapid temperature elevation throughout the system by using a ferromagnetic substance, by physically embedding Fe_3_O_4,_ into MOF‐sodium alginate, with the incorporation of hygroscopic salt CaCl_2_. As Fe_3_O_4_ acts as a magnetic susceptor, the application of an alternating magnetic field (AMF) induces a uniform and rapid heat generation throughout the system via eddy current and magnetic hysteresis loss, which enables rapid water desorption and produces approximately 9.11 L_H2O_ /kg _MOF‐AG@IO_ /day at 75% RH at 25°C in three successive sorption cycles. Each cycle lasted 60 min, consisting of 30 min of water adsorption and 30 min of water desorption [118]. Studies done on the topic of electro and magneto‐stimulated hydrogels for water harvesting applications are listed in Table 6.

**TABLE 6:** Electrothermal, magnetothermal materials for water adsorption and water harvesting.

Stimuli‐responsive components	Matrix material	Performances
Photothermal component—CNT Electrical stimuli—Ionic hydrogels [73]	Poly (sodium 4‐styrene sulfonate)—(PAAS) 3‐acrylamidopropyl [trimethylammonium] chloride (DMAPAA‐Q)/polyacrylamide	Water and electricity generation ∼ 95 wt.% water desorption in 30 min ∼ 400 mV achieved by using 20 cells in series ∼ 45 h of power generation at 90% RH
Electro‐thermal reagent—GO [24]	Porous graphene network	Water adsorption capacity 4.15 gg^−1^ at 90% RH Heat storage capacity 6.93 kJ g^−1^ Cyclic stability > 10
Fe_3_O_4_‐ photothermal and magnetothermal component [23]	Thermally conductive Acrylamide matrix	Under 1 sun‐ 2.33 kg m^−2^ h^−1^ Under AMF – 1.64 kg m^−2^ h^−1^ Under 2 sun + AMF – 4.54 kg m^−2^ h^−1^
Fe_3_O_4_‐ photothermal and magnetothermal component [118]	Sodium alginate/MOF‐808	∼ 85% of water is getting desorbed within half an hour ∼ 10.3 L kg^−1^ day^−1^ in outdoor

Despite the recent research articles about the promising performance of magnetically stimulated hydrogels, the major limitations of the current literature are the inconsistent reporting of magnetic field operating parameters. Some other studies specified the applied magnetic field strength of AMF, 1.5–3.4 mT, for regenerating oxygen plasma‐treated magnetic flower‐like porous carbon containing embedded cobalt nanoparticles with a water yield of 4.5 L_H2O_ kg^−1^day^−1^ through uniform heating of a 2 cm thickness solid sorbent [119]. Sharma et al. reported the applied induction currents (100.7–171.0 A) and specific absorption rate (SAR) (22.7‐50.1 W g^−^^1^), but the AMF frequency and magnetic field strength were not disclosed here [118]. On the contrary, a static magnetic field with field strength of 1429.2‐2748.3 G was employed for an Fe_3_O_4_/PVA membrane with an evaporation rate of 1.3236 kg m^−2^ h^−1^ under one sun irradiation [120]. These observations highlight the need for comprehensive reporting of magnetic field characteristics to ensure a clearer understanding of the relationship between the applied magnetic field parameters and the sample used for water harvesting.

#### 2.7.3 Multi‐Stimuli Responsive “Smart” Hydrogels

**FIGURE 10:**
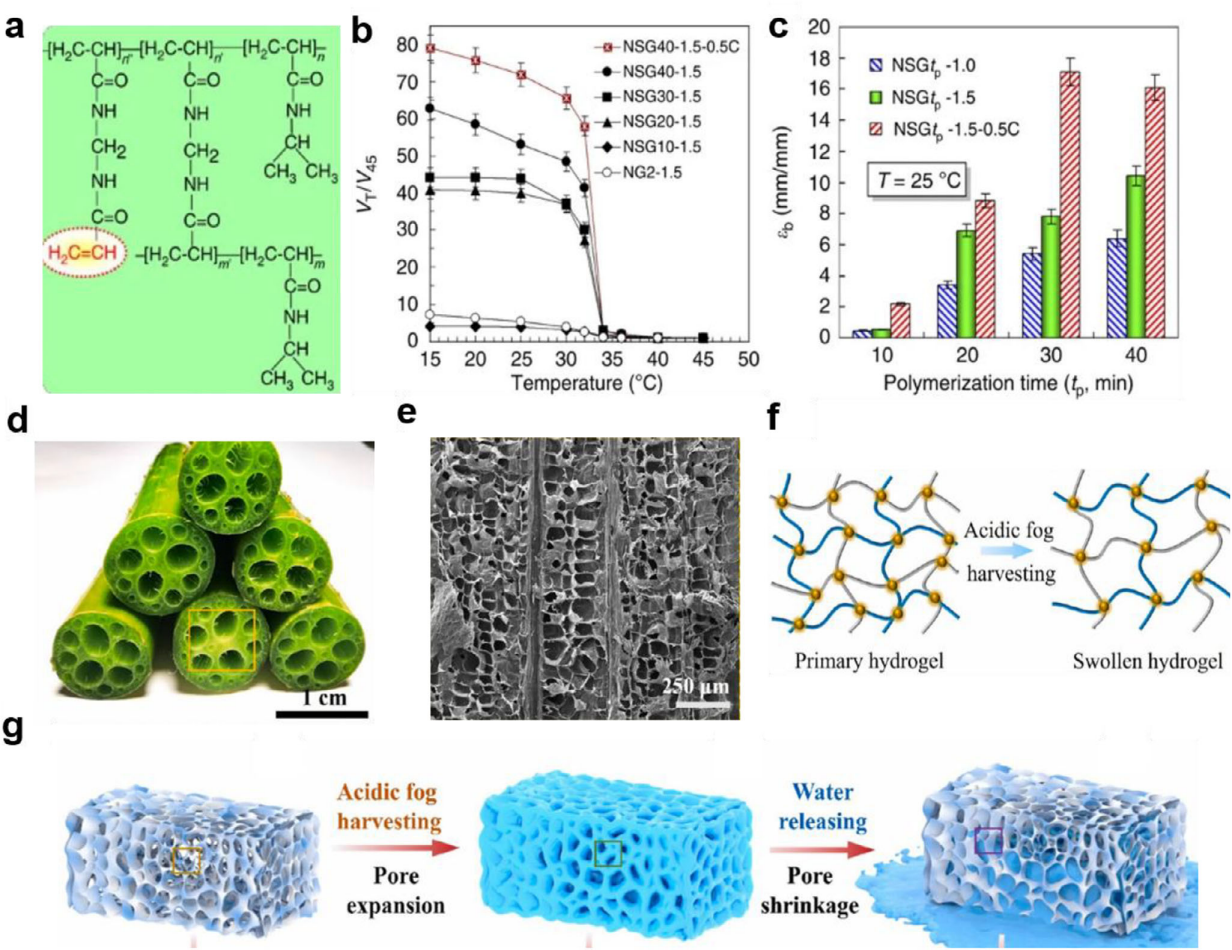
a) Chemical structure of the activated nanogels bearing unsaturated double bond fabricated by polymerization of NIPAM by using MBA, a crosslinking agent. b) Temperature dependence swelling behaviour of NSG and NG hydrogels, where V_T_ and V_45_ present the equilibrium volumes of the hydrogel at temperature T and 45°C, respectively. c) Effect of the polymerization time of ANG, concentration of NIPAM monomer, and ANG nanogel content on the elongation ratio at break (ε_b_) of NSG hydrogels with different compositions measured at room temperature. Reproduced with permission from ref [21]. Nature, copyright 2013. d) and e) Representative of Optical and scanning electron micrographs of the lotus stem. f) Schematic representation of the primary hydrogel before and after acidic water swallowing. g) Schematic presentation of acidic fog capture by the presence of a pH‐sensitive PDMAEMA network, subsequent desorption of water triggered by thermal stimulation. Reproduced with permission from ref [124]. Elsevier, copyright 2024.

Smart hydrogels are the most precisely engineered polymeric networks with minimal toxicity, integrated by multiple functional groups, enabling them to respond to the application of two or more stimuli. Additionally, these hydrogels can be synthesized through double network formation, in situ crosslinking, or by combining natural and synthetic polymers [121, 122]. Smart hydrogels are known for their ability to change their volume or other properties by the application of external stimuli such as pH, temperature, magnetic or electrical effects, which has significantly increased the field of application, such as optical systems, biomimicry applications, tissue engineering, drug delivery, etc. Immediate and significant response to external stimuli, high elasticity is the most important properties of smart hydrogels [21]. Thermo‐responsive hydrogels based on PNIPAM, including systems with grafted side chains and interconnected porous hierarchical structures were generated to improve the aforementioned challenges; though they improved the response rate of the hydrogel, but failed to help the elastic property. The elastic property of the interpenetrating polymeric network or hydrogel nanocomposite with clay crosslinkers is enhanced, but their swelling property is still limited due to intermolecular interaction between the polymers. Xia et al. and scientists synthesized a nanostructured hydrogel (NSG) via controlled polymerization of poly(N‐isopropylacrylamide) (PNIPAM) by using N,N‐methylene‐bisacrylamide (MBA) as a covalent crosslinking agent; the probable chemical structure is shown in Figure 10a. This NSG shows an instantaneous response to external stimuli such as temperature, a significantly higher swelling ratio and elongation at break compared to conventional hydrogels (Figure 10b). By increasing the concentration of NIPAM, the thermo‐responsive equilibrium swelling ratio increased up to 8000%, which is about 10 times higher than that of a conventional hydrogel (PNIPAM hydrogel crosslinked by small molecule MBA), where NIPAM has not been used as a bridging agent. Upon increasing the temperature beyond the LCST of NIPAM, the hydrogel shrinks rapidly, without any restriction of crosslinking. During adsorption of water at 25°C, the elongation at break of NSG hydrogel reaches up to 1710% (Figure 10c) and at 37°C, the PNIPAM chains undergo a quick phase change from hydrophilicity to hydrophobicity within 6 min, leading to chain entanglement and formation of reversible physical crosslinking. This is a rapid change in characteristic is a classic example of smart or intelligent hydrogel [21].

Liu et al. also developed an intelligent hydrogel with the same composition of PNIPAM crosslinked with MBA, enables a “smart” gesture by showcasing a rapid volume change through phase transition and dramatic shrinkage [123]. Under an acidic fog environment, the tertiary amine groups of (Figure 10f,g) pH‐sensitive PDMAEMA become protonated, and markedly increase the chain hydrophilicity and osmotic swelling. Upon mild heating above the LCST of PNIPAM, the polymer network undergoes a reversible hydrophilic to hydrophobic phase transition, expelling the stored water with high efficiency (90% desorption within ∼12 min at 60°C). Shown in Figure 10d,e the lotus stem‐inspired interpenetrating porous architecture, together with high cycling stability (∼ 25 cycles) and the copolymerization of these two polymers, generates an “intelligent” hydrogel, as the water‐polymer interactions are not dynamically regulated by external stimuli. In this case, one single hydrogel can be stimulated by two different stimuli, one is the pH of the system, and the other is temperature [22, 124]. Here comes another example of a multi‐stimulated hydrogel which is simultaneously responsive to a magnetic field and pH. A starch‐based hydrogel, consisting of vinyl‐modified starch with N’, N’‐dimethylacrylamide (DMAAm) and acrylic acid (AAc), in the presence of CoFe_2_O_4_ nanoparticles, exhibits dual responsiveness to pH and applied magnetic field. At pH 2, the application of a magnetic field produced no noticeable changes despite the presence of magnetic nanoparticles, due to the dense and rigid crosslinking of starch‐DMAAm matrix; here, the water transport is mainly governed by pseudo‐Fickian diffusion. However, when the acrylic acid was incorporated in the system, slight swelling was observed even at pH 2 due to very limited hydration of protonated carboxylic groups. But in the case of pH 7 and 10, the carboxylic group gets deprotonated and introduces negative charges in the polymeric backbone; the resulting electrostatic repulsion between ‐COO^−^ groups cause the expansion in the network and significantly enhances water uptake to values exceeding 30 g g^−1^ [22]. Smart hydrogels not only respond to changes in pH, magnetic field, or temperature, but also respond to the effect of zwitterionic groups present in the polymeric chains. Conventionally, for making a hydrogel, hygroscopic inorganic salts such as CaCl_2_, LiCl, MgCl_2_, etc., were used because of their exceptionally high‐water adsorption capability. Later, salt‐embedded interconnected porous hydrogel composites were developed based on polymers such as poly(N‐isopropylacrylamide), exhibiting excellent water sorption ability. However, these systems faced several challenges because of their non‐electrolytic nature. To overcome those limitations, the incorporation of a photothermal reagent in a zwitterionic polymeric matrix, poly‐[2‐(methacryloyloxy)ethyl] dimethyl‐(3‐sulfopropyl) ammonium hydroxide (PDMAPS) with hygroscopic salt LiCl is important. The swelling ratio of PDMAPS/CNT increases almost ninefold by the incorporation of LiCl in water due to the salting‐in effect. The Li^+^ and Cl^−^ ions interact with the polymers anionic and cationic groups, respectively. That's how the salting‐out phenomenon can be completely restricted here. Additionally, the photothermal effect of CNT provides a photothermal effect by converting the adsorbed light into heat and facilitates water evaporation from the system [54]. Noticeably, scientists have not limited their research on the application of photothermal materials solely in reducing the desorption energy barrier. Researchers have emerged with an efficient photothermal material, Black graphitic carbonitride (black g‐C_3_N_4_), not only because of its ability to absorb a broad solar spectrum, but also for its excellence in interfacial water evaporation and photocatalytic degradation of toxic biomedical organic pollutants, for water purification [125]. The integration of such multifunctional systems in a bio‐based hydrogel matrix improves water transport with environmental remediation, which provides important and valuable chemical design principles for next‐generation smart hydrogel systems [126, 127]. As a whole, these studies on smart hydrogels demonstrate that by tailoring the chemical composition, network structure, a multi‐stimuli‐responsive hydrogel can achieve enhanced mechanical properties, faster sorption kinetics, tunable swelling behaviour and can be applied for multifunctional applications as well under various stimuli.

Stimuli‐responsive organic or inorganic materials incorporated into the polymer matrix or using stimuli‐responsive polymers significantly lower the water regeneration energy compared to conventional semi‐active polymeric hygroscopic materials. Each thermoresponsive polymer has a specific LCST; crossing that temperature causes a reversible transformation from hydrophilic to hydrophobic, or vice versa, enabling cyclic water absorption and desorption [40]. Photothermal materials accelerate desorption kinetics by efficiently converting solar energy into localized thermal energy. Similarly, electrically, magnetically or pH‐stimulated hydrogels facilitate precise and rapid desorption via joule heating, magnetic induction or alteration of the system pH.

Reports are showing that with increasing the percentage of photothermal, magnetic or conductive nanofillers, the sorption efficiency increases; however, excessive filler inclusion reduces the equilibrium water uptake by lowering the specific surface area and effective hydrophilic volume, blocking the mass diffusion pathways, or increasing the sorption kinetics. Another important bottleneck is maintaining a homogeneous dispersion throughout the matrix without any aggregation or precipitation; otherwise, a uniform property will not be maintained throughout the system. Smart or intelligent hydrogels contain more than one stimuli‐responsive hydrogel, which requires a sophisticated synthesis route, inclusion of multiple functional components, with higher fabrication cost. This fabrication complexity often questions the scalability of a smart hydrogel and reusability over long‐term reproducibility.

The lack of standardized protocols such as humidity levels, solar intensities, regeneration methods, number of cycles, etc hinders the direct comparison between different studies. Other important parameters such as electric or magnetic field strength, power density, induction current are frequently reported incompletely.

Future research should move beyond maximizing the overall efficiency. Establishing standardized testing protocols, reporting complete operating parameters, evaluating long‐term outdoor performance under realistic climatic conditions, and quantifying specific energy consumption (kJ L^−1^ of harvested water) will be essential for enabling meaningful comparisons and accelerating the practical deployment of stimuli‐responsive hydrogel‐based atmospheric water harvesting technologies.

## 3 Hydrogel Network Architecture and Water Capture

### 3.1 Performance Metrics Tailored to Hydrogels

To evaluate the performance of hydrogel‐based sorbents such as AWH, some well‐defined metrics that reflect their unique polymer and water‐responsive characteristics. Hydrogels are soft materials with high flexibility and porosity; unlike rigid materials, they exhibit dynamic swelling behaviour. This makes parameters like water uptake capacity and swelling ratio critical indicators of sorption performance. Now, as water uptake and swelling behaviour have been stated, it is also noteworthy to mention that these behaviors highly depend on absorption kinetics and thermodynamic parameters. The relationship between network structure, crosslink density, and hydrophilic functional groups greatly influences the speed of vapour diffusion within the polymer matrices. However, the number of sorption‐desorption cycles greatly depends on the regeneration energy and cyclic durability. Structural fatigue, hysteresis, and polymer breakdown can harm long‐term stability. Therefore, assessing hydrogel systems requires a balance between capacity, performance speed, and energy efficiency. These specific performance metrics allow for a more realistic evaluation of hydrogel‐based materials for atmospheric water harvesting and similar applications. Eventually, the metrics should necessarily be compared with the AWH performance by MOFs, hygroscopic salts, and porous carbons. This truly highlights the potential of hydrogels as compared to other materials and also signifies the impact of the performance optimization strategies

#### 3.1.1 Water Uptake Capacity vs Swelling Ratio

Evaluation of AWH performance on their swelling properties and water uptake capacity does not require a costly or high‐tech experimental setup; it can be determined by simple volumetric methods and elastic expansion of the hydrogel polymeric networks. Although water uptake capacity and swelling ratio apparently look similar, they are not linear to one another; in fact, they are synergistic. An expansion of the polymeric network diminishes the sorption barrier within the hydrogel, enlarges the pores and facilitates the infiltration of water, whereas the absorbed water further pushes the polymeric network to its maximum elastic limits. Hence, an optimal balance between both factors is necessary to tailor the AWH performance.

The equilibrium water uptake of a salt‐loaded hydrogel is primarily governed by the percentage of hygroscopic salt present in the matrix as well as the physicochemical properties of the polymer matrix. The sorption capacity of a hygroscopic salt embedded in composite material (HSCM) can be expressed by Equation (1),

(1) $$\omega_{HSCM}=x\omega_{salt}+\left(1-x\right)\omega_{matrix}=x\omega_{salt}\;$$

**FIGURE 11:**
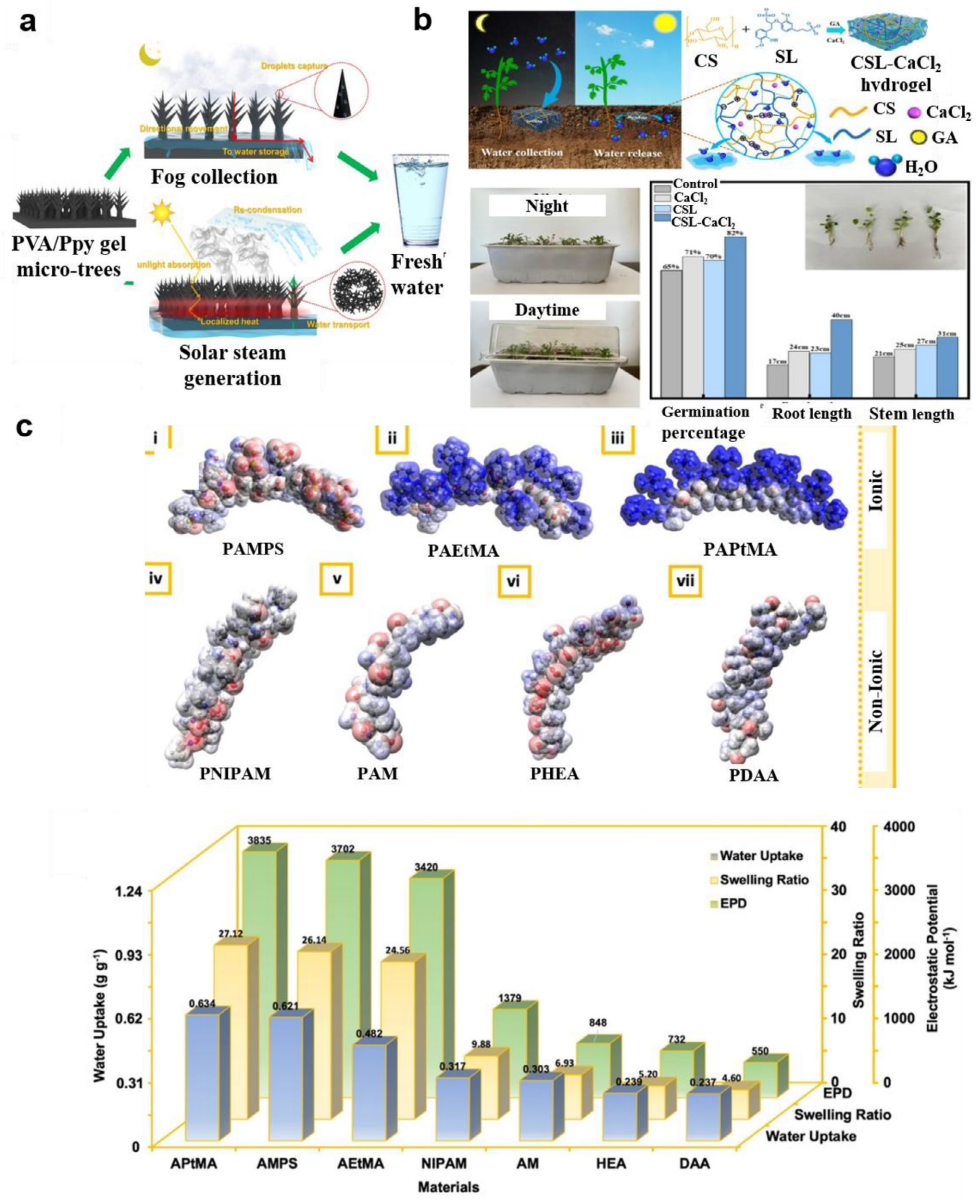
a) Illustrative design for a bifunctional gel membrane prototype for all‐day water‐harvesting. Reproduced with permission from ref [88]. Springer, copyright 2021. b) Illustrative design of the hydrogel and its effects on plant growth. Reproduced with permission from ref [96]. Elsevier, copyright 2025. c) Computational calculation of swelling ratio, water uptake, and electrostatic potential difference of different polymer chains. Reproduced with permission from ref [133]. Wiley, copyright 2024.

***x*** the mass fraction of hygroscopic salt, ***ω***_***salt***_ and ***ω***_***matrix***_ is the equilibrium water uptake of the salt and polymer matrix, respectively [128]. The intrinsic sorption capacity of the polymer is much lower than that of ionic hygroscopic salt (ion‐dipole energy ∼ 10–50 kJ/mol and dipole‐dipole interaction energy ∼ 5 to 20 kJ/mol, hydrogen bonding energy ∼ 10–40 kJ/mol) [129, 130, 131]. Increasing salt percentage in polymeric matrix generally enhances the overall sorption capability of the hydrogel. However, excessive salt incorporation leads to leaching, salt agglomeration, pore blockage and leakage of salt solution during cyclic sorption cycles. So, to get the maximum yield, a balance between the swelling capacity and optimum concentration of salt is required to preserve structural integrity, interconnected pores for smooth transportation of water and long‐term stability. Several design principles have been implemented for polymer and hygroscopic salt‐based AWH systems. One such great execution can be noticed in the recent work of Shi et al. [88] They have engineered a hydrogel membrane with micro‐tree topology, which can act as an all‐day water harvester. The clever design of the membrane expands the surface area, which can collect more fog droplets at night and transport them to a storage vessel. During the daytime, the stored water again evaporated and recondensed to produce fresh water by an interfacial solar steam generation system (Figure 11a). With an impressive evaporation rate of 3.64 kg m^−2^ h^−1^ under 1 sun, a homemade rooftop water harvesting system had collected around 34 L m^−2^ of fresh water in an outdoor experiment [88]. The fog collection rate was found to be 5.0 g cm^−2^ h^−^1, which is again 115% higher than commercial alternatives and 615 higher than a real cactus stem. Zhao et al. fabricated another super moisture‐absorbent gel with a unique combination of hygroscopic polypyrrole chloride and hydrophilicity switchable poly‐NIPA networks. Excellent design of the gel generates a synergistic effect at the molecular level between the hygroscopic and hydrophilic‐switchable properties [103]. Based on this effect, the gel can absorb a high amount of water through in situ liquification and rapid water discharge under various weather conditions. Unlike others, Bai et al. approached the issue differently, targeting soil water harvesting by collecting atmospheric water. Their amphiphilic hydrogel system was composed of sodium lignosulfonate/chitosan/calcium chloride, which can rapidly absorb at a 1.34 kg∙m^−2^ rate by swelling during the night and during the day, it gets squeezed to release the absorbed water to the soil at a 0.81 kg∙m^−2^ rate. The said way of water harvesting was found to be extremely beneficial in improving the physiochemical properties of soil by reducing soil density by 21.6%, enhancing the organic matter content in soil by 144%, and uplifting the plant germination rate by 17% (Figure 11b) [96].

A remarkable example of fine‐tuning the swelling ratio to water uptake capacity was demonstrated by Lei et al. by the use of anti‐polyelectrolyte effects in polyzwitterionic hydrogel. The hydrogel could capture 5.87 L kg^−1^ of freshwater per day. Unique salt‐responsive properties and anti‐polyelectrolyte effects enable the hydrogel to quickly absorb atmospheric water with simultaneous improvement in the solubility and swelling properties [57]. Guo et al. have designed their super‐hygroscopic polymeric films composed of renewable biomasses (Konjac glucomannan & hydroxypropyl cellulose) and hygroscopic salts to show their AWH properties, especially in arid atmospheres (RH < 30%). This highly porous network structure facilitates an impressive water uptake rate of 0.64–0.96 g g^−1^ at 15%–30% RH [3]. The polymeric films could operate 14–24 cycles a day, leading to a commendable yield of 5.8‐13.3 L kg^−1^ fresh water per day. Cost‐effectiveness and a simple method of fabrication through casting stand out for the scalability of these films to tackle the global water crisis. Yang et al. utilized both sunlight and wind energy to fabricate a water harvesting device. Sunlight accelerates the condensation of the polymeric network as well as desorption synergistically; however, wind flow drives the sorption kinetics. The device has shown a high efficacy in producing fresh drinkable water at a 14.9 L m^−2^ day^−1^ rate with a thermal efficiency of 25.7% during the indoor experiment, while during the outdoor experiment, the rate was recorded as 3.5‐8.9 L m^−2^ day^−1^, without consuming any power, totally driven by natural sunlight [132]. The polyelectrolyte hydrogel of Shan et al. was engineered to create a very high osmotic pressure, which leads to ultrafast cyclic sorption‐desorption kinetics. The hydrogel was tuned for 8 operating cycles per day, producing 2.41 L of water per kg of sorbent; this amount is 3.5 times higher than the usual single‐cycle operation model. After a wide range of explorations of experimental methodologies, the section can be concluded perfectly by highlighting the recent structure‐property study of AWH hydrogel systems via a computational approach done by Feng et al. To reduce the experimental cost and the trial‐and‐error time, they have analyzed various AWH systems based on their physicochemical properties, electrostatic potential and the water uptake capacity. Eventually, they concluded that among all their trial systems, the AWH hydrogel made up of PAPtMA stood out as the best in terms of electro potential difference, swelling ratio and water uptake (Figure 11c) [133].

#### 3.1.2 Kinetics: Vapor Diffusion and Transport in Polymer Matrices

The importance of fine‐tuned sorption‐desorption kinetics has been mentioned in the previous section. Vapour transport in polymeric AWH systems is generally governed by several factors, such as Fickian diffusion, capillary‐assisted transport, and polymeric network‐controlled swelling [53]. Low RH often facilitates the initial penetration of water into the polymeric network, which further interacts with the accessible hydrophilic groups in the polymeric chains. However, at high RH, swelling enlarges the pore volume, leading to an accelerated vapour sorption. Now, the effective diffusion coefficient again depends on multiple factors, such as crosslink density, chain flexibility, and pore connectivity. On the other hand, an optimized content of the hydrophilic‐hydrophobic moieties can prevent water from infiltrating deeper toward the polymeric core, which invariably reduces the regeneration time and energy consumption, leading to a greater number of cycles per day. To describe the kinetics of vapour as well as liquid diffusion in the micropores of hygroscopic hydrogels, a two‐concentration model (represented in Equations 2 and 3) was proposed by Marin et al. [134].

(2) $$\frac{\partial \left(\Phi_{g}C\right)}{\partial t}\;-\;\frac{\partial }{\partial x}\left(D_{eff}\frac{\partial C}{\partial x}\right)=NSD_{V}\left(\frac{a_{w,s}P_{sat}}{RT}-C\right)$$

(3) $$\left(1-\Phi_{g0}\right)\;\frac{\partial n}{\partial t}-\frac{\partial }{\partial x}\left(D_{l}\frac{\partial n}{\partial x}\right)=-\;NSD_{V}\left(\frac{a_{w,s}P_{sat}}{RT}\;-\;C\right)$$

***Φ***_***g***_** Local volume fraction of gas phase, ***Φ***_***g*** 0_** Initial volume fraction of gas phase, ***D***_***eff***_** and ***D***_***l***_ The effective diffusivities of vapour and liquid transport, respectively, *C* molar vapor concentration in air, *N* is micropore number density, *S* single micropore's mass transport shape factor, ***a***_***w***, ***s***_ function type of the salt, temperature and mass fraction of salt in water, ***P***_***sat***_ standard state saturated vapour pressure of water at temperature T, n is the molar water concentration in the hydrogel relative to it's dry volume.

In this study, they elucidated these two equations to describe both the vapour and liquid transport processes separately. The theoretical study was further supported by experimental data. Key findings described a thinner layer of hydrogel with a higher shear modulus, and well‐tuned initial porosity could help in attaining much faster kinetics. In later years, Marin et al. further developed another physics model to describe the moisture‐capture properties of hydrogels [135]. In this research, they conducted extensive thermodynamic and dynamic measurements on salt‐hydrogel composites. The developed model was found to be fruitful in accurately measuring the moisture uptakes and sorption enthalpies. Equation 4 shows that mass transfer between the hydrogel and the ambience is driven by the difference in water vapor concentration between them. They assumed that the hydrogel and the environment are at the same temperature. This assumption accurately describes experiments where absorption and desorption are driven by relative humidity rather than temperature. Further, they have established that diffusion processes have a negligible effect on the sorption‐desorption kinetics, and convection was solely responsible for the phenomenon. They have quantified the same with a numerical value called “Biot number” (Equation 5), which is nothing but the ratio of diffusion resistance to convection resistance. For a convection‐driven process, this value should be < 0.1. As a major outcome, the negligible role of polymeric material in the overall AWH performance emerged as one of the most interesting facts of their research. However, the choice of salt, its content and the thickness of the hydrogel stood out as the determining factor of AWH performance. The model can be further utilized in exploring the potential of moisture capture for freshwater production, energy storage, and sustainable cooling.

(4) $$\begin{array}{ccc} \frac{\partial C_{w}}{\partial t} & = & \frac{g}{H_{0}}\;\left(RH.{\left(\frac{P_{sat}}{RT}\right)}_{amb}-\;y_{w}x_{w}\cdot {\left(\frac{P_{sat}}{RT}\right)}_{hyd}\right)\\ & = & \frac{g}{H_{0}}\frac{P_{sat}}{RT}\;\left(RH-\;y_{w}x_{w}\right) \end{array}$$

***g *** Convection coefficient of mass transfer, ***H***_0_ **initial thickness of hydrogen at its most desorbed condition, ***C***_***w***_ **molar concentration of water relative to the hydrogel's volume at its most desorbed condition, ***y***_***w***_ water's activity coefficient in salt solution, ***x***_***w***_ mole fraction of water relative to the moles of water and ions, ***P***_***sat***_ **The water vapor's saturation pressure at temperature T, *R* is the universal gas constant, *RH* is the relative humidity.

(5) $$Bi=\frac{R_{diff}}{R_{conv}}=\frac{H_{0}g}{D_{w}}\frac{i.C_{s}.\frac{P_{sat}}{RT}}{{\left(C_{w,\infty }+i.C_{s}\right)}^{2}}$$

*Bi* Biot number, ***R***_***conv***_ convection resistance between air and hydrogel surface, ***R***_***diff***_ diffusion resistance inside of the hydrogels matrix, ***H***_0_ hydrogel's thickness in the initial condition, ***g*** mass transfer convection coefficient, ***D***_***w***_ diffusion coefficient of water in the sorbent, ***C***_***s***_ salt concentration in the hydrogel matrix, ***P***_***sat***_ saturation vapour pressure of water, *R* universal gas constant, *T* absolute temperature, ***C***_***w***,∞_ molar water concentration in the hydrogel, ***i*** number of ions per salt molecule.

Vapour adsorption on a porous and nanostructured hydrogel system is governed by surface adsorption process, and this process typically follows the Brunauer‐Emmett‐Teller or BET theory. BET model describes the multilayer adsorption of gas or vapor molecules on a surface, which is widely useful in determining the specific surface area of porous material from the adsorption isotherm data. So, in the field of water harvesting from atmosphere, this BET theory is highly applicable for moisture adsorption on a polymer‐based porous hydrogel system, the BET equation can be written as follows, Equation (6) [136].

(6) $$w=\frac{w_{m}cRH}{\left(1-RH\right)\left(1-RH+cRH\right)}$$

In this equation, ***w*** is the gravimetric water content (g g^−1^), ***w***_***m***_ is the monolayer water content and c is the enthalpy of adsorption‐related constant

RH allows the determination of the monolayer adsorption capacity and adsorption constant from experimental adsorption isotherm data. This ***w***_***m***_ can further be used for estimation of specific surface area (SSA) of adsorbent materials by following this Equation (7) [136].

(7) $$\mathrm{SSA}\;=\frac{w_{m}\;N_{A}N_{A}}{M_{w}}$$

In this equation, ***A***_***m***_is the area occupied by each water molecule, ***M***_***w***_is the molar mass of water [136].

The available specific surface area governs the numbers of adsorption sites for water molecules, in helps in determination of the adsorption capacity and kinetics [137].Materials with smaller particle diameter exhibits higher specific surface area, facilitates rapid adsorption kinetics. For example, a hydroxyapatite nanoparticle with 10 nm diameter exhibits a specific surface area of 206 m^2^ g^−1^ with significantly higher water sorption capacity in comparison with a 40 nm particle diameter, where the latter ones SSA is 49* * ± * *1 m^2^ g^−1^, which clearly exhibits the strong influence of SSA for adsorption performance. The adsorption isotherm fitting also reveals whether the multilayer physical adsorption is possible or not and also it helps to determine the number of adsorbed water layers by depending on the particle size and environmental humidity conditions. In porous polymeric hydrogel system after adsorption moisture diffuses throughout the network and induces swelling [103].

**FIGURE 12:**
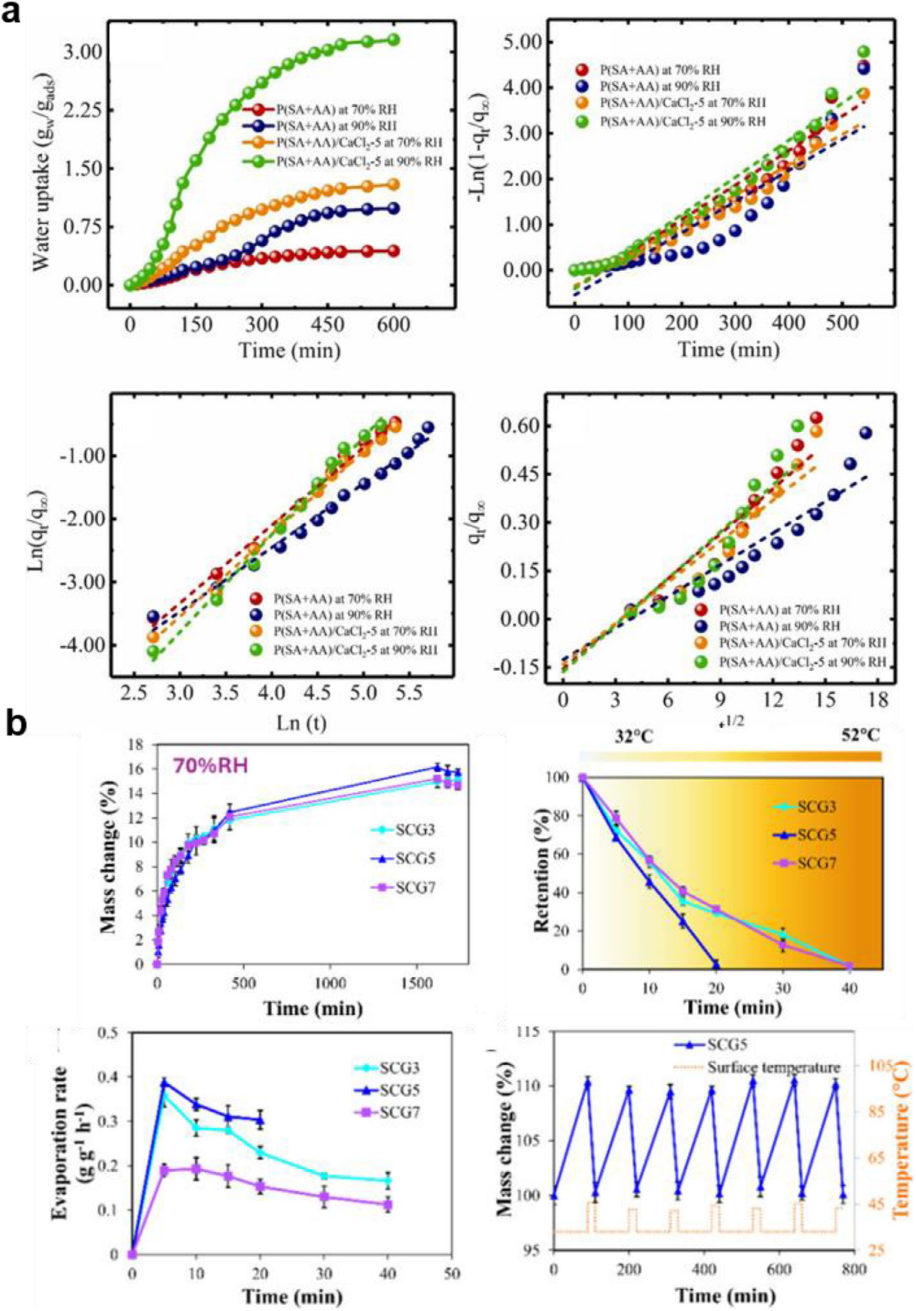
a) Variation of different kinetic parameters (LDF model) against change in RH. Reproduced with permission from ref [138]. Elsevier, copyright 2021. b) Time‐dependent sorption‐desorption performance of the crosslinked hydrogel. Reproduced with permission from ref [140]. ACS, copyright 2025.

Mittal et al. also performed a study on the kinetics and adsorption isotherms of deliquescent salts [138]. This type of salt has a very high affinity toward water absorption, but once they absorb water, they get dissolved in it, forming crystalline hydrates. This phenomenon restricts its use in multi‐cyclic sorption‐desorption applications. Hence, the feasible solution is making a hydrogel out of it, where the salt remains dispersed in the polymeric matrix. Hence, they used acrylic acid and sodium acrylate system with CaCl_2_ (a deliquescent salt). The fabricated super‐porous hydrogel has shown an increment from 1.02 g to 3.35 g of water adsorbed per gram of adsorbent with the addition of CaCl_2_ salt. In both cases, the experimental data obeyed the Guggenheim, Anderson and Boer (GAB) isotherm model (Equation 8), and exhibited the type‐III adsorption isotherm, whose kinetics followed the linear driving force (LDF) model (Equation 9). The GAB isotherm model highlights the occupation of water molecules on the surface of the adsorbent as a monolayer. This model also explains how the elevated temperature reduces the adsorption process. Whereas the LDF model was introduced to correlate experimental kinetics data with the mathematical model (Figure 12a). Further investigation suggests that the material basically has two regions. The first region works actively at low RH, where the deep penetration of water is not possible due to low pressure inside. Thus, here the water generally gets attached to the surface moieties. On the other hand, at higher RH values, penetration to the core is possible, allowing the water to become entrapped through attachment to the CaCl_2_ salt. This system can be used easily to absorb water droplets from moist air.

(8) $$q\;=\frac{q_{m}c_{G}k_{G}a_{w}}{\left(1-k_{G}a_{w}\right)\left(1-\;k_{G}a_{w}+c_{G}k_{G}a_{w}\right)}$$

***c***_***G***_ and ***k***_***G***_ is representing the Various constants that belong to the multi‐ and monolayer adsorption, respectively, ***a***_***w***_ **the value of the water activity, which is equal to relative humidity and *q* is the equilibrium water adsorption capacity

(9) $$q_{t}=q_{\infty }\left(1-e^{-k_{\mathrm{eff}}t}\right)$$

***q***_***t***_ **Amount of vapour adsorbed by the hydrogel at time t, ***q***_∞_ **maximum adsorption capacity of hydrogel at equilibrium, ***k***_***eff***_ **effective mass transfer coefficient

There are also plenty of examples available on experimental‐based approaches with well‐optimized sorption kinetics. Recently, Li et al. introduced a bidirectionally aligned and hierarchically structured nanocomposite system as an efficient water harvesting solution [139]. The design of this nanocomposite itself is an engineering marvel. The nanocomposite was fabricated using a bidirectionally aligned porous graphene hydrogel matrix with LiCl coating. The manufacturing process includes bidirectional freezing (using a specially designed Cu‐plate with arrays of Cu‐pillars), crosslinking, and LiCl loading. This structure works synergistically by accelerating moisture convection through vertically oriented channels, along with radially oriented pores for inter‐channel diffusion. Smart engineering is responsible for an outstanding water uptake rate of 6.61 kg per kg of absorbent with superfast sorption kinetics. The prototype is not only scalable, energy‐efficient, and rapid‐cycling but also capable of producing 2.82 L of fresh water per day per kilogram of sorbent. Maity et al. worked extensively on polymer chemistry to develop an AWH device with a core‐shell structure. The core was designed using alginate and polyaniline, with an outer layer of poly(*N*‐isopropylacrylamide) (PNIPAM) having sulfonic acid groups attached to it. After loading LiCl, the material was found to have a similar LCST as pristine PNIPAM (32°C), while the water‐capturing efficiency increased by over 15‐fold. The desorption can be achieved easily with a minimal sunlight intensity of 1 kW m^−2^. In a single day, the device could produce 6.5 L of water (RH ≥ 17%) with no external power supply [138]. Nguyen et al. performed a study on the variation of sorption kinetics with the change in cross‐linker concentration (Figure 12b). They evaluated the moisture uptake mechanism, sorption rate, as well as rate determining step through a list of non‐linear kinetic models, applied to the moisture sorption data of non‐crosslinked and crosslinked samples at 90% RH value. The entire kinetics can be divided into three major steps. In the first step, external diffusion occurred when the water molecules accumulated on the outer boundary. In the second step, internal diffusion takes place when the water droplets move inside the hydrogel through its pores. Eventually, at the last step, sorption happens on the active sites present inside the hydrogel. This step is the slowest and generally considered the rate‐determining state [140].

#### 3.1.3 Thermodynamics, Regeneration Energy and Cycling Durability

Regeneration energy is undoubtedly a very crucial parameter while tailoring the AWH hydrogel metrics. It unfolds the feasibility of that hydrogel system to repeat its performance cyclically. Regeneration energy can be optimized based upon several factors, like the extent or nature of interaction between water and functional groups in the polymeric network, free vs. bound water content, extent of network relaxation during desorption, ratio of hydrophilic and hydrophobic groups, etc. [141]. A fine‐tuned crosslinking also prevents the irreversible network collapse in the hydrogel during desorption and thus reduces the regeneration energy for cyclic performance. Durability is another important factor, as during cyclic experiments, a hydrogel often goes through frequent thermal and humidity fluctuations, repeated swelling‐deswelling also induces a volumetric strain to the system, which might turn into network fatigue, pore collapse, and loss of active sites [142].

The water sorption behaviour of hydrogel is primarily governed by thermodynamic equilibrium between the external and internal environments of the system. During equilibrium, the chemical potential of the adsorbed water should be equal to the surroundings of the system. This determines the water absorption and desorption process. Flory's theory can be used to describe the thermodynamic behaviour of the hydrogels. This considers the contribution of the three most important factors: entropy of mixing, polymer elasticity and polymer‐solvent interactions. The change in ∆G, Gibbs' free energy of the hydrogel, can be expressed by Equation (10) [143].

(10) $$\begin{array}{ccc} \Delta G & = & k_{B}T\left(n_{1}\ln f_{1}+n_{2}\ln f_{2}\right)+k_{B}T\chi n_{1}f_{2}\\ & & +\;\frac{k_{B}\;\nu_{e}T}{2}\left(3{a_{s}}^{2}-3-\ln{a_{s}}^{3}\right) \end{array}$$

here,

$$f_{1}=\frac{n_{1}}{n_{1}+Zn_{2}},\;f_{2}=\frac{Zn_{2}}{n_{1}+Zn_{2}},\;{a_{s}}^{3}=\frac{V}{V_{0}}=\frac{V_{0}+n_{1}V_{1}}{V_{0}}=\frac{1}{f_{2}}$$

This expression describes the driving forces responsible for swelling and water absorption. ***k***_***B***_ Boltzmann constant, *T* absolute temperature, ***n***_1_ water molecules inside the hydrogel network, ***n***_2_ number of polymer molecules in the hydrogel network, ***f***_1_ volume fraction of solvent, ***f***_2_ volume fraction of the polymer, ***a***_***s***_ chain extension ratio during swelling, ***χ*** polymer solvent interaction, ***ν***_***e***_ excludes the two terminal ends of the polymer chains, after (*V*) and before(*V_0_*) swelling, *Z* volume ration of the polymer molecule to the solvent molecule.

**FIGURE 13:**
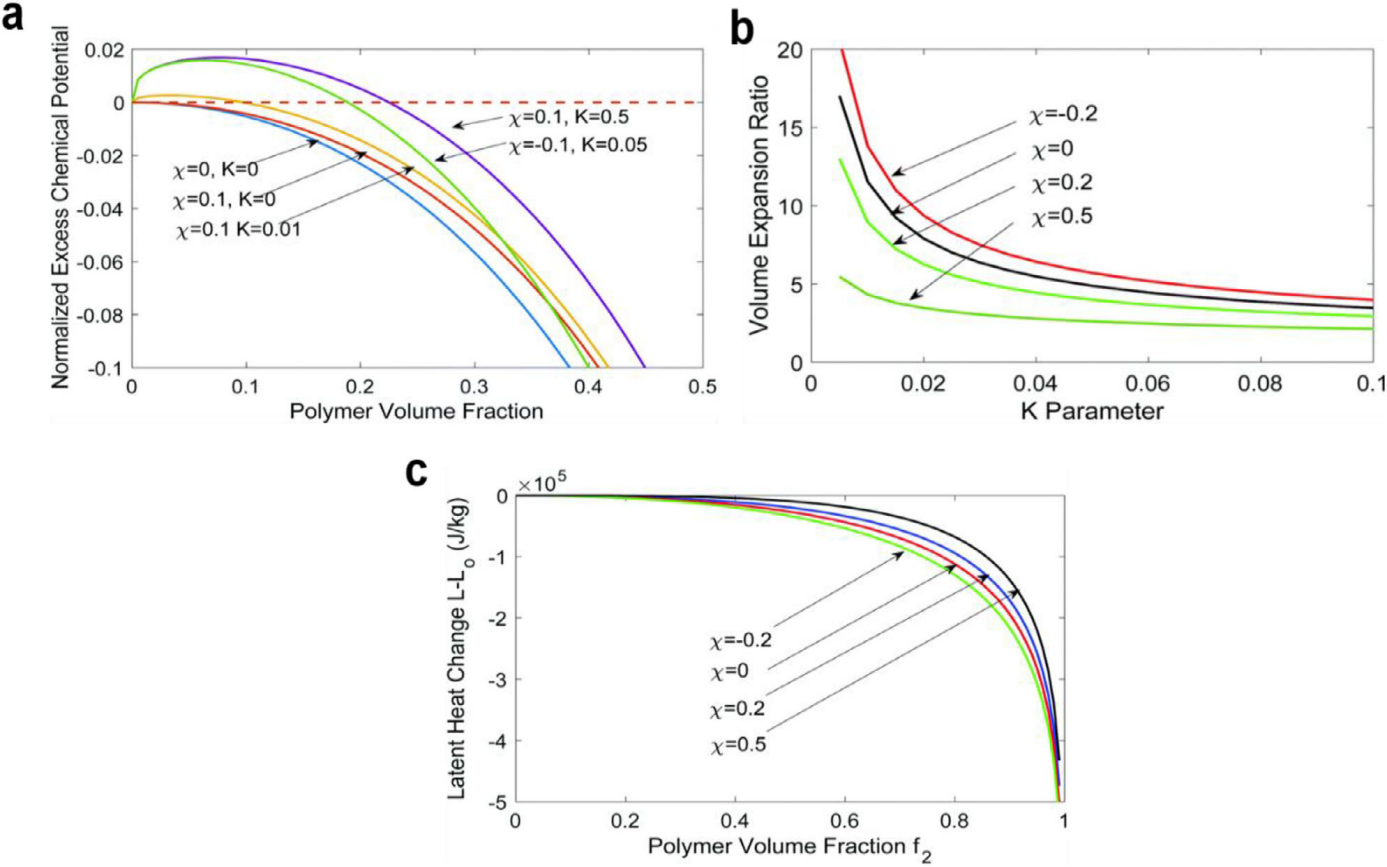
a) Plot of normalized excess chemical potential of water vs polymer volume fraction. b) Volume expansion ratio vs crosslinking parameter K under several ***χ*** values. c) Reduction of latent heat at a constant temperature of 300 K, at different ***χ*** values as a function of polymeric volume fraction. Reproduced with permission from ref [143]. RSC, copyright 2022.

Inside a hydrogel matrix, the internal osmotic pressure is much higher than the ambient pressure. The chemical potential influences the hydration interactions with the polymer network as well as the elastic deformation of the crosslinked hydrogel. So, the change in chemical potential can be derived from the change in Gibbs free energy by 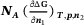 on a molar basis, Equation (11) and (Figure 13a) [143].

(11) $$\begin{array}{ccc} \Delta \mu & = & {\left(\Delta \mu_{1}\right)}_{pl}+{\left(\Delta \mu_{1}\right)}_{el}=RT\;\left[\ln\;\left(1-f_{2}\right)+\left(1-\;\frac{1}{Z}\;f_{2}+\chi f_{2}^{2}\right)!\right]\\ & & +\;\frac{RT\nu_{W}n_{e}}{V_{0}}\left(f_{2}^{\frac{1}{3}}-\;\frac{f_{2}}{2}\right) \end{array}$$

*N_A_* = Avogadro number, *F_pl_* = Helmholtz free energy change, *pl* = polymer network, ***μ***_1_chemical potential of the water molecules in the hydrogel, ***ν***_***W***_ molar volume of the solvent, ***W*** Flory‐Huggins interaction parameter, ***V***_0_ intial polymer volume

At equilibrium, the internal and external osmotic pressures are equal. Hydrogel swelling is governed by solvent components and the polymer's elastic restoring force. So, during thermodynamic equilibrium, dF_t_ = 0. According to the Flory‐Rehner model, the internal osmotic pressure can be expressed as in Equation (12) [143].

(12) $$P-P_{e}={\left(\frac{\partial \left(\Delta F_{pl}\right)}{\partial V}\right)}_{T,n_{2}}=\frac{RTn_{e}}{2V_{0}}\left[2{\left(\frac{V}{V_{o}}\right)}^{-1/3}-\frac{1}{\left(\frac{V}{V_{o}}\right)}\right]$$

*P, **P***_***e***_ **internal and external pressure of the hydrogel, respectively, *P—**P***_***e***_ osmotic pressure difference, which drives the hydrogel swelling.

Furthermore, the thermodynamic state of internalized water influences its desorption or evaporation behaviour. The latent heat needed for the evaporation is given by Equation (13) [143].

(13) $$\begin{array}{c} L=L_{0}+RT\;\left[\ln\;\left(1-\;f_{2,eq}\right)+f_{2,eq}+\chi {f_{2,eq}}^{2}\right]+RT^{2}{f_{2,eq}}^{2}\frac{\partial \chi }{\partial T} \end{array}$$

***L***_0,**_ ***L*** **L**atent heat of evaporation of pure water and evaporation inside the hydrogel, respectively, ***f***_2, ***eq***_ **during equilibrium polymer volume fraction inside hydrogel, ***χ *** polymer solvent interaction

This equation signifies that evaporation enthalpy can be altered by the alteration of the polymer, which is a relevant factor for the application of the AWH system in a thermally‐responsive evaporation [143].

Taking the above points into consideration, Li et al. developed a prototype AWH system based on the theory of optimal harvesting window (OWH), which is nothing but a principle that helps to find out the best‐balanced parameters based on thermodynamics, on which the hydrogel can show its highest efficiency and lowest regeneration energy. According to this theory, both water collection rate and energy efficiency can be improved simultaneously by decreasing the sorption temperature and increasing the humidity, which mimics the environmental “Dew Point”. They surveyed 11 different climates (13°C–35°C, 18%–72% RH) all around the globe and proved their concept by performing a 5‐h‐long experiment at every place. Findings suggest that the prototype loaded with 3 kg of silica gel could easily produce 2.25 L of fresh water within 5 h and 5.76‐17.64 L per day, depending upon the climate. On average, their prototype could last up to 30 cycles, maintaining an excellent energy efficiency of 0.46‐1.5 L/kWh without any degradation. The conclusion of the study was drawn from some simulation results as a future direction of this research. According to the results, an optimized cooling recovery was also implemented to enhance energy efficiency by 37.8% – 92.4%, without compromising water production [144].

Zhao et al. fabricated a multi‐layered thermoresponsive hydrogel water‐harvesting system. The thermoresponsive nature of the hydrogel facilitates the hydrophilic‐hydrophobic transition with relatively low threshold energy for sorption‐desorption cycles. The system was able to collect 2.05 L kg_sorbent_^−1^ per day with an average energy efficiency of 26.4% [142]. Similar work was also reported by Zhan et al., where they have incorporated polypyrrole to get that same photothermal performance with a synergistic advancement in hygroscopic property as well. The N‐atom present within the polypyrrole facilitates the H‐bonding formation with absorbed water molecules and reduces the leakage of the same. Experimental results demonstrated that the effective hygroscopic performance was noticed at RH 30%‐90%. At RH 70%, 25°C, 1.53 g water was collected per g of adsorbent in 12 h. The sample remains as it is even after 10 simultaneous sorption‐desorption cycles [70]. A hybrid sorbent was developed by Elwadood et al. utilizing regenerated alginate precursor and porous silico‐aluminophosphate‐34 (SAPO‐34). Enhanced hydrophilicity, stability, fast‐channel transport, and presence of graphene‐based sheets as a light absorber are the key modifications introduced in the system on adding SAPO‐34, all these results in a record intake of 6.85 g of fresh water per g of sorbent at high RH (like 90%) and 1.14 g of water at low RH (like 20%). Desorption could be achieved using natural sunlight only; no external power source is required. The system could easily last up to 30 cycles without any drop in efficiency or any deterioration [145]. Wang et al. throve a LiCl‐loaded monolithic porous hydrogel via the foaming‐dry method consisting of interconnected macropores (50 – 400 µm), which boosts vapour transport within the system. This system, too, could last up to 30 cycles without any salt leakage, maintaining an equilibrium water uptake of 5.28 g/g at 90% RH. Under natural sunlight, 3 m/s airflow, and other ambient conditions, a dew‐point‐triggered condensation leads to a sorption‐desorption cycle of just 45 min. The system has registered a water yield of 0.147 L/(m^2^·h) and thermal efficiency of 12.3% [146, 147]. Qui et al. went a step ahead to design a solar‐driven device that can simultaneously do photocatalytic H_2_ production and atmospheric water harvesting. The integrated device possesses a water self‐supply layer (WSL) and a photocatalytic layer (PL) to continue both processes side by side. Under 1 sun illumination, the device could efficiently produce H_2_ at a 25.98 mmol m^−2^ h^−1^ rate and condensed water at an 81.78 g m^−2^ h^−1^ rate. After a day‐long experiment, 118.21 mmol m^−2^ of H_2_ production and 230 g m^−2^ of condensate water were obtained. This device is a classic example of how we can tackle the water crisis and generate green energy altogether [50, 91, 147].

### 3.2 Network Architecture and Porosity Engineering

Parameters such as, crosslinking density, hierarchical or uniform distribution of pores, pore or mesh‐size, tortuosity factor and polymer composition, etc. Directly affects the mass and heat diffusion kinetics. Therefore, a precise design of the network from nano to macro‐scale is crucial to balance the water uptake, diffusion efficiency, structural stability, and durability. This particular section is about how the crosslinking density, size of pores, copolymerized architectures, and incorporation of foreign materials define the performance of hydrogel systems.

#### 3.2.1 Crosslinking Density and Hierarchically‐Porous Hydrogel

Crosslinking density is the number of interaction points connecting the polymeric chains per unit volume in the hydrogel network. It influences the mechanical properties of the hydrogel, including the swelling ratio, diffusion rate, water retention efficiency, mesh size, etc. As the multilevel ionic crosslinking density offers different strengths, which serve different aspects in maintaining the structural integrity of the hydrogel network. Recent studies demonstrate that multiple levels of ionic crosslinking comprise both weak and strong ionic crosslinked domains. Weak ionic interactions act as reversible sacrificial bonds that dissipate mechanical energy during uptake of water, whereas strong ionic bonding maintains the structural integrity. The swelling capacity of a hydrogel is inversely proportional to the crosslinking density, as reduced crosslinking allows the expansion of bonding and mesh size; however very low crosslinking compromises with mechanical robustness and cyclicity, similarly hydrogels with very high crosslinking density suffers from mechanical stiffness and reduced swelling ability. During adsorption after condensation, physically bound moisture starts to diffuse inside the network, while the weak crosslinked sites dissociate, swell and store the water till complete saturation without collapsing the scaffold. An optimized and uniform crosslinking density can produce a uniform porous size, higher toughness, exceptional tensile strength, fatigue resistance, while a nonuniform distribution of the crosslinking generates a dense, brittle and collapsed network structure. Experimental studies confirm that optimize crosslinking can dramatically enhance the tensile strength while preserving the same water content [58].

**FIGURE 14:**
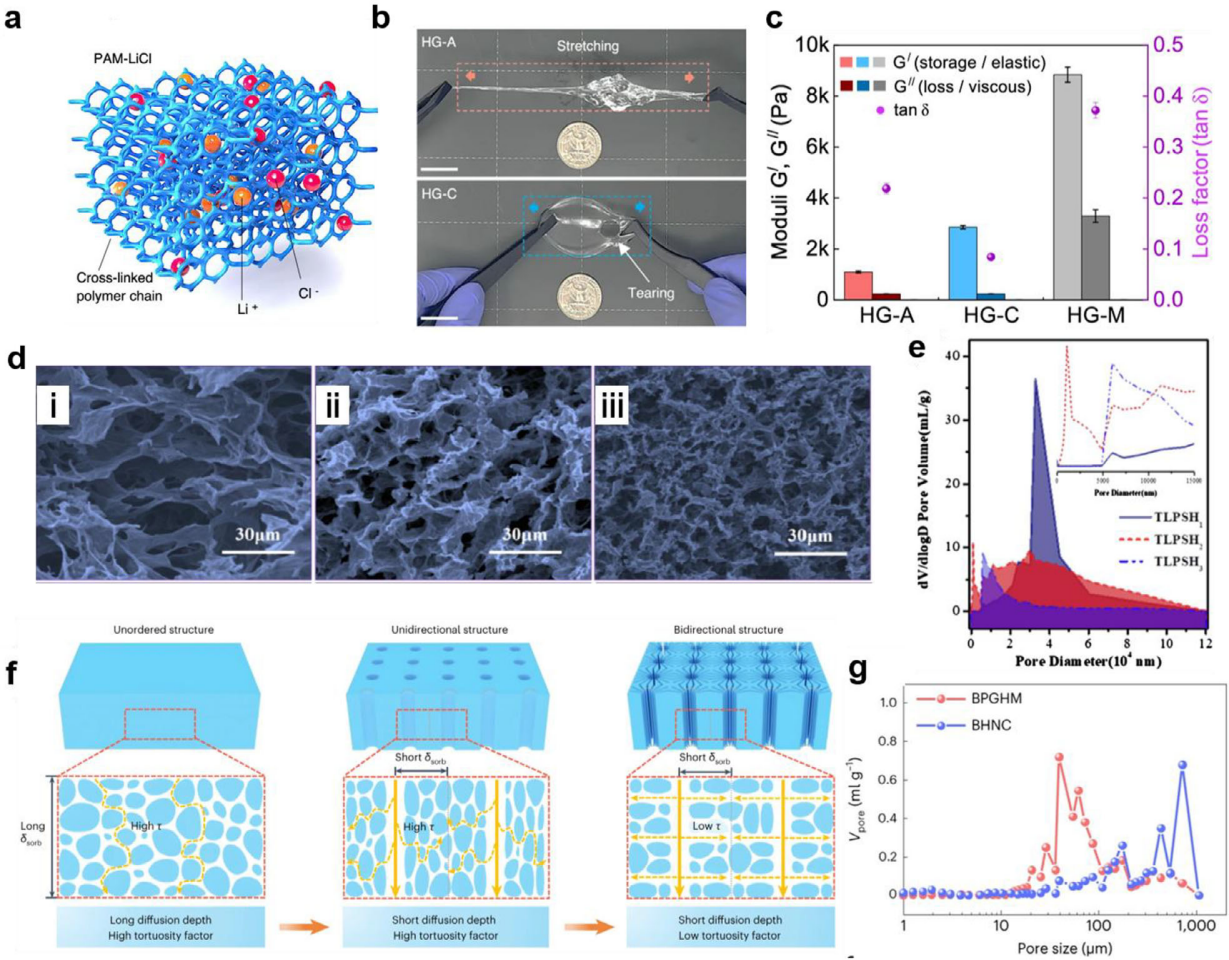
a) Schematic representation of PAM‐LiCl hydrogel structure with diffused lithium and chloride ions into the hydrogel matrix. b) Photograph representing the PAM‐LiCl hydrogels inside and outside the Petri dish, demonstrating the contrast between the samples with the highest and the lowest (HG‐A) and (HG‐C) storage modulus, respectively. c) Mechanical properties of the prepared samples were measured at 25°C, with an oscillating load of 6.3 rad/s. Reproduced with permission from ref [27]. Nature, copyright 2025. d) SEM image of pore distribution and cross‐sectional view of pore structure of TLPSH_R_ hydrogel with variable solid proportion, TLPSH_1,_ TLPSH_2,_ and TLPSH_3,_ respectively. e) Pore size distribution by MIP. Reproduced with permission from ref [151]. Elsevier, copyright 2022. f) Morphological representation of the evolution from amorphous to bidirectionally ordered structure of solid sorbents with improved heat and mass transportation. g) Pore size distribution of bidirectionally aligned and hierarchically structured nanocomposites and salt‐coated bidirectionally aligned porous graphene hydrogel matrix. Reproduced with permission from ref [139]. Nature, copyright 2023.

Shuvo et al. reported that with increasing the crosslinking density (0.1 mg/g to 10 mg/g) in PAM‐LiCl hydrogel, as illustrated in Figure 14a, the water uptake capacity is almost constant, but the energy consumption to desorb the water is almost doubled with a significantly lowered desorption rate. There is a deterioration in non‐Fickian diffusion, chain mobility, and swelling capacity as well [27]. Most particularly, the crosslinking density directly affects the mechanical properties, Figure 14b,c clearly demonstrating the change in stretchability and change in storage and loss modulus respectively [27]. In order to maintain a mechanically robust, interconnected porous structure with low mass and heat transfer resistance, a flexible network and high‐water adsorption and desorption capacity, an optimized crosslinking density is needed. It can only be developed by maintaining a precise composition of monomer, crosslinker and salt concentration [148]. Knapczyk‐Korczak et al. studied that a multiscale fibrous network with micro to nano hierarchical architecture markedly enhances the moisture capture and transportation through the electro spun (PS‐PA(6) meshes formed through a physically crosslinked network, where the fine PA6 nanofiber created dense domain, which increases the specific surface area and capillary‐driven condensation [149].

Beyond the chemical and ionic crosslinking, other types of secondary crosslinking introduce reversible dynamic interactions which influence the swelling ability, chain mobility, mesh size and cyclic stability. Guo et al. reported that in a supramolecular hydrogel network, hydrophobic dimers work as a physical crosslinker within a hydrophobic nanodomain. Hydrophilic chains are working as a bridge between those crosslinkers; the longer the bridges, the lower the crosslinking density, the larger the mesh size and vice versa. An optimized distribution of UPy‐PEG domains resulting in a flexible crosslinked architecture with high swelling capacity, mechanical and structural integrity over repeated hydration and dehydration cycles [64].

For the case of swelling‐based applications like hydrogels used for AWH systems, the crosslinking density can be estimated by using Flory‐Rehner equations (Equation 14) [150].

(14) $$\begin{array}{c} -\left[\ln\left(1-v_{2}\right)+v_{2}+x_{1}\;v_{2}^{2}=\frac{V_{1}}{\bar{v}M_{c}}\right.\;\left(1-\frac{2M_{c}}{M}\right)\left(v_{2}^{\frac{1}{3}}-\frac{v_{2}}{2}\right) \end{array}$$

***v***_2_ is the polymer volume fraction, ***V***_1_ signifies the molar volume of the solvent, ***x***_1_ is the parameter to determine Flory solvent‐polymer interaction, ***M*** is the primary molecular mass, ***M***_***c***_ represents an average molecular mass between crosslinks.

In parallel to the crosslinking density, uniform pore size and distribution as well as hierarchical porosity of a hydrogel play a crucial role in mass and water vapour diffusion, capillary‐driven transport, etc. across different hydrogels, the pore size is broadened up to a large scale, they are typically categorized into 3 different sections—nanopore (1‐50) nm, meso/micropores (50‐10) µm, macropores (10‐1000) µm. The exact pore size varies in different studies, depending on the needed application, synthetic routes and sample composition.

Hydrogels with hierarchical pores contain nanopores to interconnect macropores (50 – 350 µm), which facilitates bulk transportation of water, while nanopores increase specific surface area for moisture adsorption and lower the evaporation enthalpy, capillary condensation. Meanwhile, the macropores improve moisture diffusion, and enhanced salt‐ion transport results in an increased sorption kinetics [151, 152]. Distribution of pore sizes with changes in proportions of materials (rGO‐PVA) and crosslinking density from nano to macrolevel is presented in Figure 14d,e [151]. Hierarchically porous hydrogels that contain 3 distinct pore size ranges make a unique contribution in enhancing water adsorption capacity. Nanopores increase specific surface area, capture moisture through H‐bonding and capillary forces, whereas micro/meso and macropores support the water cluster formation, intermediate diffusion and reduction in diffusion resistance, respectively. Also, compared to a hierarchical porous system, a uniform or narrow porous distribution faces several problems; although small pores increase the water adsorption capacity, they lower the bulk water transportation and desorption kinetics. Additionally, aggregation of nanoparticles and salt can occur. In contrast, if only particles are there, without any interconnecting channels, it reduces the effective surface area, capillary condensation and storage capacity with a very fast rate of diffusion [153]. Hydrogels with ultra‐low crosslinking density (10.82±0.32 mg/cm^3^) contain a uniform pore size of ∼ 8 nm, resulting in an increased surface area and moisture adsorption. The physical crosslinking between Silica nanofiber (SNF) and Carbon nanotube (CNF) works as sacrificial bonds, it forms, disrupts during swelling and reforms during desorption [154]. 2 scientists, Xu et al. and Li et al., both used the same crosslinking agent, Ca^2+^, which strengthens and stabilizes the hierarchical porous structure. These systems exhibit ultralow density, with extremely high porosity (∼86% and 97%, respectively), which enables a decreased diffusion resistance and low tortuosity factor [32, 139]. Also achieved a water yield of almost 3 liters of water per kg of solid sorbents per day, with ultrafast water sorption kinetics due to its bidirectional architecture with low tortuosity factor, high porosity and short diffusion depth, compared to amorphous or unidirectionally oriented hydrogels, schematic architecture is shown in Figure 14f, the systems optimum pore size 100–1000 µm works as water diffusion channels, pore size less than this suffers from salt‐agglomeration (pore size distribution graph Figure 14g) [139]. Collectively, optimized crosslinking density combined with hierarchically porosity network minimizes diffusion bottleneck, maximizes water uptake capacity, enhances sorption kinetics and preserves structural stability.

The water harvesting performance of the hydrogel is governed by a sophisticated balance between reversible physical and permanent chemical crosslinking. While lower crosslinking density and dynamic non‐covalent interactions enhance swelling, water diffusion, retention, excessive swelling compromises structural integrity, in the contrary, hydrogel systems with higher density of crosslinking improves mechanical property in the expense of higher desorption energy and sluggish regeneration. Hence, strategic optimization of crosslinking density of both covalent and non‐covalent with hierarchical porous architecture is necessary in improvement of water harvesting performance in all aspects.

### 3.3 Processing and Morphology Engineering

The role of polymer chemistry and hygroscopic interactions in atmospheric water harvesting (AWH), translating intrinsic material performance into high water productivity, critically depends on processing routes and morphology engineering. In hydrogel‐based AWH, mass transport kinetics, interfacial vapor exchange, heat localization, and scalability are governed as much by *how* the hydrogel is structured and deployed as by its chemical composition [155]. Recent advances demonstrate that rational engineering of thin films, patterned surfaces, and additively manufactured architectures enables hydrogels to overcome diffusion limitations and move toward system‐level viability [53, 57].

Reducing characteristic diffusion lengths through thin‐film and coating geometries is one of the most effective strategies to accelerate vapor sorption‐desorption cycles. Tokarev et al. highlighted early on that responsive hydrogel thin films offer distinct advantages over grafted polymer brushes, combining mechanical robustness with multi‐responsive behaviour when fabricated via chemical crosslinking, layer‐by‐layer assembly, or block copolymer self‐assembly [156]. Such films provide rapid swelling–deswelling kinetics, making them well suited for humidity‐responsive surfaces. Recent AWH‐specific studies have leveraged thin hydrogel layers to achieve efficient heat and mass transfer. Ma et al. reported a cormorant‐inspired macroporous hydrogel thin film with interconnected micrometer‐scale channels that simultaneously localize heat at the evaporation interface and maintain continuous water supply [157]. The drastic reduction in heat transfer cross‐sectional area enabled evaporation rates exceeding 4 kg m^−^^2^ h^−^^1^ under 1 sun with minimal material usage, highlighting thin films as a cost‐effective engineering route.

Layered hydrogel membranes have also been used to decouple capture and storage functions. Gao et al. demonstrated a bioinspired material segregation strategy in which a thin hydrogel membrane transports atmospheric moisture into an underlying liquid desiccant, enabling simultaneous water capture and solar‐driven release [158]. Similarly, smart hydrogel coatings for buildings reported by Jin et al. integrate hygroscopic capture with optical humidity response, illustrating the feasibility of coating‐based AWH systems embedded directly into infrastructure [159]. Coating approaches further enable scalable fabrication. Ni et al. and Zhang et al. developed blade‐coated and hydrogen‐bonded photothermal hydrogel coatings that can be applied to porous substrates, combining high evaporation rates with mechanical durability, salt resistance, and antifouling behavior [160, 161]. These studies underscore coatings as a practical bridge between laboratory sorbents and deployable devices [162, 163]. At the same time, additive manufacturing has emerged as a powerful tool to engineer hierarchical porosity and tailored exposure geometry in hydrogel‐based AWH systems. Wu et al. introduced microgel‐mediated direct ink writing to fabricate hygroscopic 3D matrices with multiscale porosity, significantly enhancing vapor transport pathways while reducing raw material usage [164, 165]. The ability to print previously unprintable hygroscopic composites marks a key advance in processing‐enabled performance. Gyroid and lattice architectures fabricated via 3D printing further optimize surface‐area‐to‐volume ratios. Liu et al. demonstrated LiCl‐incorporated hydrogel gyroids that achieve high water uptake with reduced desorption energy, enabling compact AWH and dehumidification devices [166]. Similarly, Zhu et al. reported 3D‐printed cellulose nanofiber aerogel scaffolds that confine hygroscopic salts while exposing active sites, accelerating both sorption and release kinetics [167].

Electrospinning provides an alternative route to extreme surface area enhancement. Kim et al. and Uddin et al. developed nanofibrous hydrogel membranes incorporating deliquescent salts and photothermal agents, achieving rapid vapor uptake even at low RH and solar‐driven water release with minimal energy input [104, 168]. These fibrous architectures blur the distinction between sorbent and membrane, enabling lightweight, flexible, and scalable AWH platforms. Biomimetic additive manufacturing further enables multifunctionality. Su et al. and Shi et al. combined 3D printing with ice templating to create multilevel hydrogel structures inspired by plant and desert organisms, integrating fog capture at night with solar‐driven evaporation during the day [88, 169]. Such architectures highlight how processing techniques can encode time‐dependent functionality into hydrogel systems.

Beyond bulk morphology, surface patterning plays a decisive role in condensation, droplet transport, and water collection efficiency. Zhang et al. demonstrated hydrogel‐patterned condensing surfaces where surface energy gradients and Laplace pressure differences actively pump and shed droplets, preventing flooding [170]. Bioinspired hybrid wetting patterns reported by Wu et al. and Jiang et al. further show how micro–nano structuring and wettability contrast accelerate water transport [164, 171]. Cone arrays, bumpy morphologies, and aligned nanocomposites inspired by cacti, beetles, and desert plants have been widely explored to manipulate airflow, nucleation, and droplet mobility [172, 173]. These patterned surfaces convert passive condensation into directional transport, directly improving harvesting rates without increasing energy input [109].

For real‐world deployment, processing strategies must support scalability, durability, and system integration. Meter‐scale origami hydrogel panels demonstrated by Liu et al. represent a milestone, translating hydrogel AWH from gram‐scale samples to architectural elements capable of sustained outdoor operation [174]. Purpose‐driven manufacturing frameworks proposed by Zhao et al. emphasize aligning polymer synthesis, processing routes, and device design with application‐specific constraints [142]. Several other studies have been reported in table 7 below, summarizing other engineering mechanisms of AWH systems. Collectively, these studies show that processing and morphology engineering are not auxiliary considerations but central design parameters that connect polymer chemistry to practical AWH performance.

**TABLE 7:** Different Engineering mechanisms of AWH systems.

Engineering & Processing Strategy	Material / Hydrogel System	Key Engineering Outcome for AWH
Hydrogel‐coated fog‐collection wires [175]	Calcium alginate hydrogel‐coated wires	Enhanced fog interception and drainage; up to 136% higher collection efficiency vs bare wires
Hydrogel‐coated antifouling evaporators [161]	TA‐PVA hydrogel on porous foam	Salt resistance, antifouling behaviour, stable evaporation (3.64 kg m^−^^2^ h^−^^1^ under 1 kW m‐^2^ irradiation)
Nanoporous inorganic–hydrogel hybrid [176]	LiCl@PHEA on silica lattice	Salt (> 90%) confinement, swelling ratio is 11.18 g g^−1^ at 90% RH in half an hour, water collection rate of 5 kg m^−2^ h^−1^
Binary salt‐modified hydrogel processing [177]	Alginate hydrogel with Li^+^/Ca^2^^+^ salts	Approximately 5.6 g g^−1^ swelling ratio at 70% RH, Avg. maximum water yield rate ∼ 0.86 g h^−1^
Hierarchical cone‐array surface fabrication [178]	PDMS cone‐array replicas	Directional fog capture via curvature‐driven droplet transport with a velocity of ∼125 cm s^−1^
Zwitterionic hydrogel–salt composites [54]	PDMAPS‐LiCl hydrogel	Salting‐in effect improves swelling ratio (without salt concentration ∼ 1.37 g g^−1^ and at 0.1 g mL^−1^ LiCl concentration ∼ 12.25 g g^−1^)
Biodegradable aerogel processing [179]	PHB/CNT/MgCl_2_ composite aerogels	Solar‐driven AWH with recyclability and soil degradability, swelling ratio ∼ 1.64 g g^−1^ at 90% RH with a rate of 1.8 g g^−1^ h^−1^ under 1 kW m^−2^ solar irradiation
Anion–cation heterostructured hydrogels [73]	Oppositely charged hydrogel junctions, electrical energy driving force	Coupled water harvesting and electricity generation, 95 Wt.% desorption in 30 min under 1 kW m^−2^
Holey fiber processing with hierarchical porosity [24]	Hygroscopic holey graphene aerogel fibers, solar and electrical energy driving force	4.14 g g^−1^ swelling ratio at 90% RH, water desorption starts in 44s under 1 kW m^−2^ solar irradiation
Bidirectionally aligned nanocomposite structuring [139]	Hierarchically aligned hydrogel nanocomposites, solar‐driven water harvesting	Accelerated mass (< 100 s m^−1^) and vapour (< 10*10^−3^ m^2^kW^−1^) transport, rate of water yield (2,000‐2,820 mL_water_ kg_sorbent_^−1^ day^−1^)

## 4 Design Strategies in the application of AWH system

The function of the hygroscopic materials is to uptake moisture from the atmosphere through physical, chemical or a combination of both adsorption processes. Compositional tuning enhances the overall efficiency, network architecture governs the diffusion of mass and temperature, structural and cyclic stability. Recent hydrogels mainly cover two principal material classes, which covers all the mechanical, chemical or physical properties. One is bio‐based polysaccharide polymers emphasizing sustainability and biodegradability, useful for biomedical, agricultural and food‐related applications. The second one is synthetic and zwitterionic systems offering precise molecular, chemical and structural tunability, improved mechanical robustness and stimuli responsiveness. Beyond the chemical composition of the hydrogel, its architecture is really important; without a well‐defined network, the absorbed water cannot be stored till a controlled desorption. In nature, there are various examples of living organisms which can survive in arid conditions by sustainably capturing, storing and releasing water. These species possess certain characteristics such as large specific surface area, directionally designed channels and angles which facilitate a less resistive route for diffusion and collection of condensed moisture. Most importantly, these are natural systems operates without any input of external energy, are only dependent upon structural design and natural stimulators for the desorption of water, which is perfect for practical applications.

### 4.1 Bio‐Based Hydrogels

Bio‐based hydrogels derived from polysaccharides such as starch, cellulose, hemicellulose, apart from chitosan, alginate, are some of the well‐approved biopolymers [180]. All these biopolymers are well accepted in the agricultural, biomedical, or food industry because they are biodegradable, cost‐efficient and extensively available. An extensive intra and intermolecular hydrogen bonding in native system restricts the chain mobility and reduces the accessible sorption sites, which results in limited hygroscopicity and slower rate of diffusion. Recently, scientists have been focusing on different strategies to tailor the hygroscopic polymers for making hydrogels, such as the incorporation of hygroscopic salts, tuning pore architecture, changing the composite formulations etc.

**FIGURE 15:**
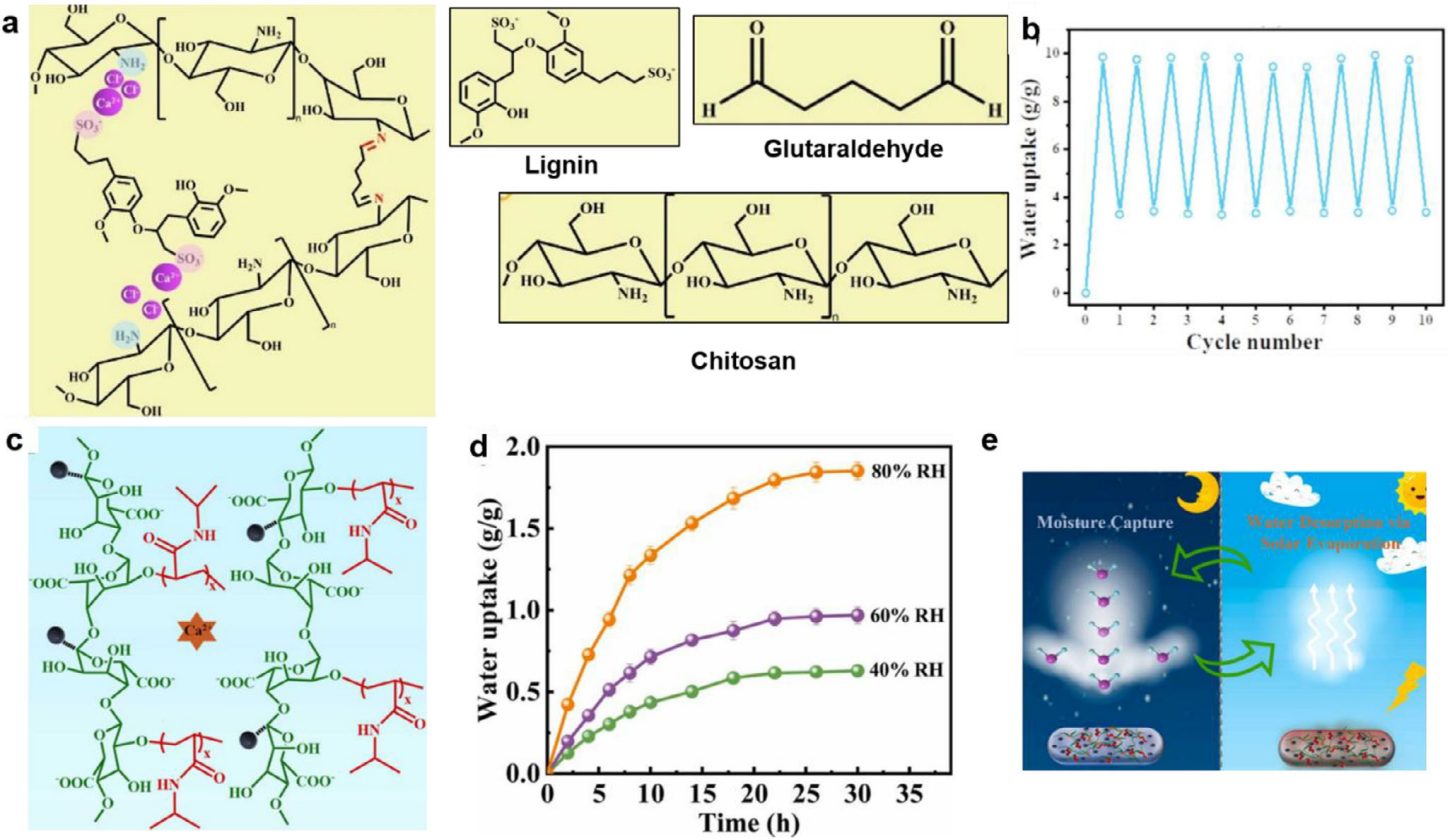
a) Chemical structure of hydrogel composed of biocompatible and biodegradable components. b) Repeated 10‐cycle durability test of biocompatible CSL‐CaCl_2_ hydrogel in soil for water harvesting at 90% RH, under 25°C, for 12 h. Reproduced with permission from ref [96]. Elsevier, copyright 2025. c) Chemical structure of the Ca‐Alg‐g‐PNIPAm/DND. d) Moisture adsorption curve of the hydrogel under 40%, 60%, 80% of RH, respectively at 25°C. e) Schematic representation of water capture and release during night and daytime. Reproduced with permission from ref [101]. Elsevier, copyright 2023.

For example, Guo et al. developed a super‐hygroscopic hydrogel by using a biobased material, cellulose, and to induce thermal responsiveness and hydrophilicity, it was modified with hydroxypropyl. Hydroxypropyl cellulose (HPC) was crosslinked with Konjac glucomannan (KGM) as the matrix, with uniform distribution of salt, LiCl. The hygroscopic salt and HPC facilitate moisture adsorption even at 15% RH. The hierarchically porous structure was provided by KGM, which induced a large surface‐air interface. Per day, water harvesting of this hydrogel (13.3L kg^−1^ per day) is sufficient to fulfil the daily needs of a human being [3]. Another example of a biocompatible hydrogel was developed by using Chitosan and sodium lignosulfonate loaded with hygroscopic salt CaCl_2_, which can be used in the agricultural field (Figure 15a). Water adsorption primarily occurs due to the strong hydration affinity of the CaCl_2_ salt and hydrogen bonding interactions provided by the hydroxyl and amine groups in the lignosulphonate and chitosan. Later, due to solar irradiation during the day (Figure 15b), as the temperature of the system increases, hydration shells collapse, H‐bonding breaks, internal water molecules gain energy, their vapour pressure increases, enabling diffusion of water through the porous scaffold into the soil without any transition to vapour phase, which helps in agriculture [96]. Zheng et al. developed a biobased hydrogel by composing Sodium alginate, derived from a polysaccharide, as a polymer structural matrix grafted with PNIPAM and integrated with hygroscopic salt calcium chloride (CaCl_2_), and photothermal detonation nanodiamond (DND) (Figure 15c) [101]. Owing to the presence of polar functional groups such as hydroxyl, carboxyl, carbonyl, and amide groups in, along with high salt loading and sufficient interconnected porosity, the microporous hydrogel can uptake water sufficiently (Figure 15d). During the daytime, due to the photothermal property of DND, it increases the temperature of the whole system, which triggers the PNIPAM to release the previously absorbed water (Figure 15e). Molecularly functionalized biomass‐based hydrogels, such as hydroxypropyl cellulose, hydroxybutyrate starch, and hydroxybutyrate chitosan, are used to break the intramolecular H bonding, to improve water solubility and induce the LCST behaviour, which enables the desorption of water at just above room temperature. Also, zwitterionation of the polymers was performed to improve hydrophilicity and salt tolerance and suppress salt crystallization. All of these help to uptake water at 15% of Relative humidity and enable rapid release of water just after crossing the LCST (∼ 45–60°C), up to 95% of water is getting desorbed at 60°C. The outdoor test demonstrates the water production rate of 1.44 kg_water_/kg_sorbent_/day [40]. Alam et al. developed a cost‐effective, highly porous, green super‐sorbent hydrogel by using biomaterial‐derived modified cellulose and carboxymethylated chitosan without any expensive catalysts. This material is suitable for applications such as wound dressing, hygiene products, agricultural uses, etc. and exhibits an exceptionally high‐water retention capacity (WRC = 610 g/g) [181]. Similarly, Entezari et al. and scientists synthesized a hydrogel based on sodium Alginate consisting of M‐blocks and G blocks enriched with highly hydrophilic Li^+^ and Ca^2+^ ions and reinforced with functionalized carbon nanotubes, which increases solar absorption as well as mechanical stability. This system achieved a rapid desorption rate and enabled portable passive water generation of 1‐ 6 g of water from just 5 g of material under complete static atmospheric conditions, without any airflow or external cooling [42].

### 4.2 Synthetic Polymer‐Based Hydrogels

**FIGURE 16:**
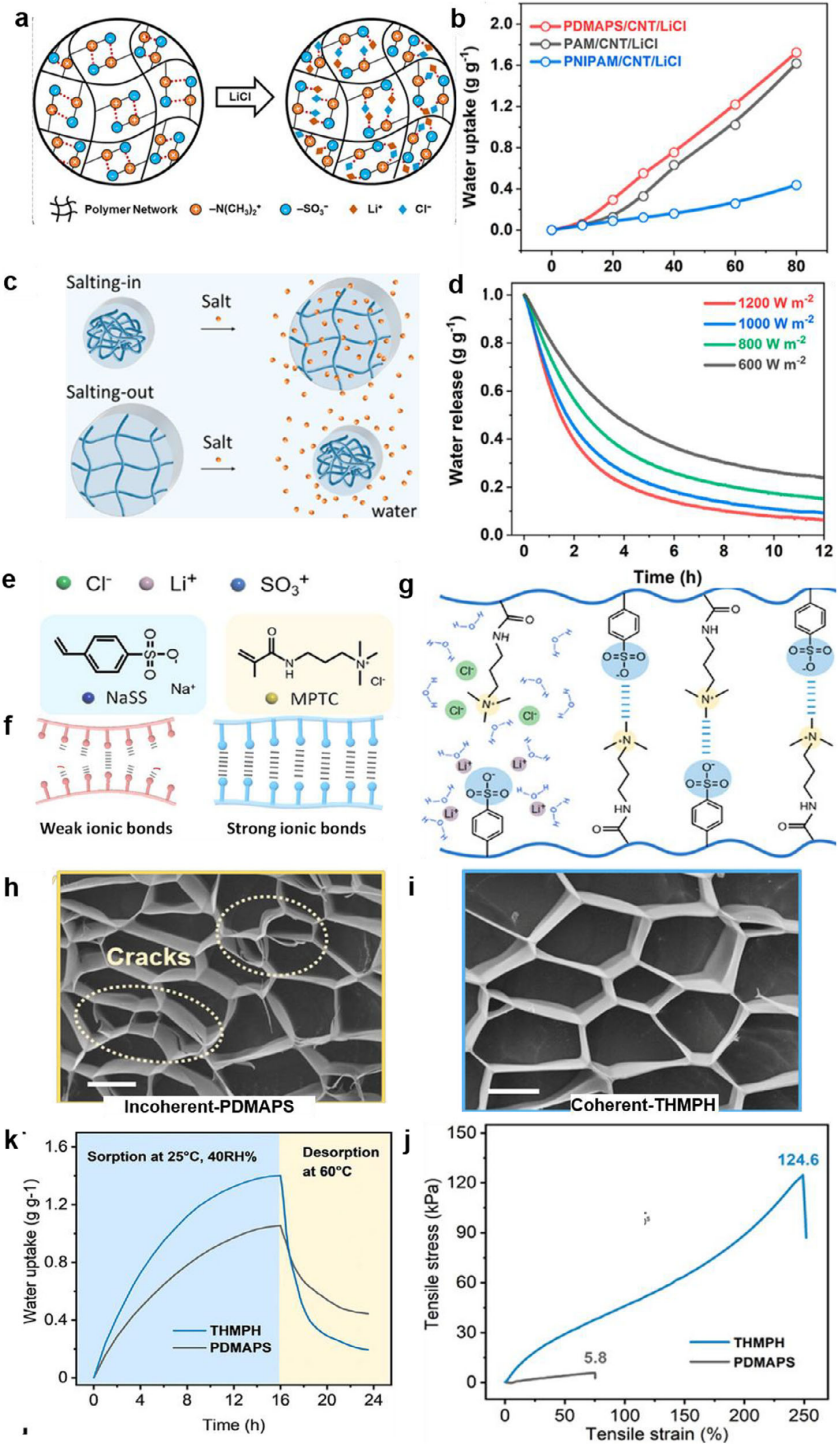
a) Schematic presentation of the hypothesized structure of PDMAPS/CNT and PDMAPS/CNT/LiCl b) Moisture adsorption isotherm of PDMAPS/CNT/LiCl, PAM/CNT/LiCl, PNIPAM/CNT/LiCl at 25°C. c) Schematic presentation of the salting‐in and salting‐out phenomenon. d) Water vapour desorption under various solar irradiations. Reproduced with permission from ref [54]. ACS, copyright 2022. e) Chemical structure of the monomer components used in this work. f) Schematic illustration of a strong and weak cross‐linked network in THMPH. g) Chemical structural representation of the hydrogel; Highly magnified SEM images of h) THMPH and i) PDMAPS with a 10 µm scale bar. j) Moisture adsorption and desorption curve of THMPH and PDMAPS. k) Tensile stress vs strain curve of THMPH and PDMAPS. Reproduced with permission from ref [58]. Wiley, copyright 2025.

Although certain bio‐based hydrogels, such as chitosan, sodium alginate, contain some ionizable electrolyte groups and act as weak polyelectrolytes in pH dependent system. In the other hand zwitterionic hydrogels intrinsically possess charge‐balanced cationic and anionic units, facilitates strong ion‐dipole and electrostatic attraction with captured moisture and salts, with salting‐in effect, ionic immobilization and structural stability. Studies reveal that though conventional bio‐based hydrogels can work efficiently under a broad relative humidity window, they require a high desorption enthalpy, which is much higher than some easy‐going stimuli‐responsive systems [182, 183, 184, 185, 186, 187]. Apart from this, because of the high cost and limited hygroscopicity, their practical application is getting hindered. So, hydrogels based on the most common synthetic polymers such as polyacrylic acid, polyacrylamide can be used due to the widespread presence of hydrophilic groups such as amide, amine, carboxylic, hydroxylic, etc. Adsorbed water molecules form strong chemical bonding with these groups. However, continuous swelling and deswelling damage the internal structure of a bio‐based hydrogel, which eventually reduces the hygroscopicity and lowers the water release rate in comparison to a synthetic polymer‐based system [188]. Incorporation of hygroscopic salts can increase the water adsorption efficiency by five to six times that of a salt‐free system [73]. Now, after adsorption of water, the polymeric network becomes mechanically weak because of dilution and reduced interchain attraction; thus, the mechanical stability of the whole system gets degraded. When scientists tried to increase the mechanical property by modifying the monomers and crosslinking agents, it resulted in a detrimental effect on the swelling efficiency of the hydrogel, which reduces the hygroscopicity [58]. Beyond a critical salt concentration, electrostatic attraction between the ionic segments of the polymer dominates, resulting in collapse of the network, deswelling, and discharge of water [96, 189]. These limitations have motivated the researchers to adopt hygroscopic polyzwitterionic hydrogels as a promising alternative which intrinsically control the ionic interactions and prevents salt induced collapse while maintaining a promising internal osmotic force (Figure 16a). Their superior moisture sorption performance, compared to a non‐electrolytic system such as PAM/CNT/LiCl and PNIPAM/CNT/LiCl has been clearly demonstrated by adsorption isotherm (Figure 16b) [54].

While incorporating LiCl into a matrix of thermoresponsive and zwitterionic polymer (PNIPAM/PDMAPS), the Li^+^ and Cl^−^ ions associate with the zwitterionic groups ‐N^+^(CH3)_2_^−^ and ‐SO_3_^−,^ respectively and stabilize the network without salting‐out (Figure 16c). With increasing temperature above 30°C–35°C, under 1 sun illumination, the molecular motion of PNIPAM increases, which induces a phase transition and a steady decline in absorption of water is noticed [66]. Fabric‐based zwitterionic hydrogels, poly (sulfobetaine methacrylate) (PSBMA), and loaded with LiCl further illustrate this approach. This system exhibits efficient moisture sorption capacity, rapid water retention, outstanding mechanical stability, well‐defined morphology, etc. Based on the above principle of zwitterionic polymer, the hydrogel does not suffer from any classical limitations such as salting‐out or salt aggregation or reduced swelling ratio [190]. Despite all these advantages, this particular zwitterionic system still suffers from several inherent limitations, such as poor mechanical robustness after swelling and salt leakage, which arise due to weak, reversible ionic crosslinking and non‐uniform polymer and salt interactions. This inhomogeneity promotes the formation of micro‐cracks and brittleness, interchain association, and sluggish water adsorption and desorption rates [58]. Therefore, Du et al. synthesized a tunable hygroscopic mix‐charged polyzwitterionic hydrogel (THMPH) by using two separately oppositely charged monomers, Sodium 4‐vinylbenzenesulfonate (NaSS) and 3‐(methacryloylamino)propyl‐trimethylammonium chloride (MPTC) (Figure 16e,f), impregnated with LiCl to challenge all the problems generated by the usage of PDMAPS. In the THMPH network (Figure 16g), the random distribution of multiple weak ionic interaction sites is collectively able to immobilize the hygroscopic salt, LiCl, resulting in minimal salt leakage (<2%). Moreover, the random distribution of oppositely charged groups prevents electrostatic self‐association, which suppresses over‐expansion upon hydration. This structural homogeneity enables uniform distribution of stress throughout the network and yields a smooth (Figure 16h), mechanically robust (Figure 16k) and faster sorption kinetics (Figure 16j) compared with conventional PDMAPS hydrogel [58].

Li et al. claimed that they developed a complementary strategy that relies on developing an interconnected structure by using an interconnected alginate‐PAM scaffold, reinforced with a photothermal agent, MWCNT and hygroscopic salt CaCl_2_. This dual‐polymeric network prevents uneven water distribution after adsorption and structural disruption after adsorption, also exhibits a rapid (3.35kgm^−2^h^−1^) release of water under exposure to light, which demonstrates how a structurally connected network can improve the mechanical integrity and desorption efficiency [188].

### 4.3 Salt‐Loaded Hydrogels

**FIGURE 17:**
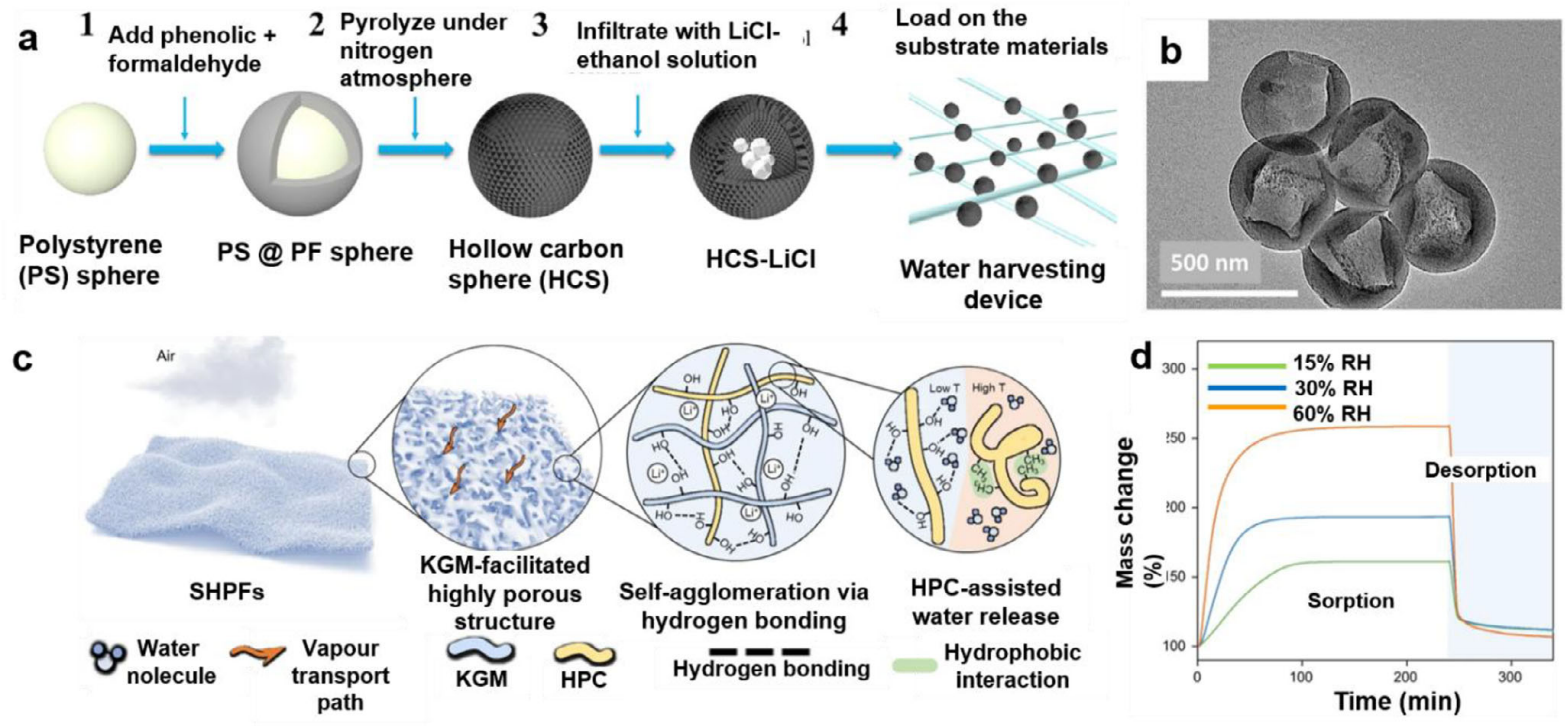
a) Schematic of the synthesis process for HCS‐LiCl nanosorbents. Step 1) PF coating on the PS hard scaffold. Step 2) obtaining HCS by pyrolysis of PS@PF. Step 3) infiltration of LiCl. Step 4) incorporation of nanosorbents into the fibrous silica framework. b) TEM image of LiCl embedded in HCS. Reproduced with permission from ref [182]. Elsevier, copyright 2020. c) Architecture of super‐hygroscopic polymer films at lower FH% (15% and 30%). d) Dynamic moisture adsorption‐desorption process at different RH%. Reproduced with permission from ref [3]. Nature, copyright 2022.

Amongst all solid hygroscopic materials, Hygroscopic salt is one of the most highly efficient, low‐cost sorbents for AWH systems, such as LiCl, CaCl_2_, MgCl_2_, LiBr, etc. These hygroscopic salts show highly remarkable water sorption capacities over a broad range of RH. The vapour uptake by a salt‐modified hydrogel is almost five times that of a salt‐free hydrogel [73, 74]. Although hygroscopic salts facilitate moisture uptake in a low‐RH atmosphere, excessive loading of salt induces deliquescence, pore blockage, mechanical failure of the polymeric scaffold. Hydrogel synthesized by Xu et al. confining one metal organic framework with LiCl salt which can harvest 0.45–0.7 kg_water_/kg_sorbent_, but there are several drawbacks such as after incorporation of salt, 60% of the pores is getting blocked, the salt concentration cannot be increased more than 51 wt.% also the system needs more than 83°C to get completely desorbed, and water uptake initiates only when the relative humidity exceeds 30% [91]. Whereas hydrogel containing LiCl in hydroxypropyl cellulose (HPC) and konjac glucomannan (KGM) bio‐material (Figure 17c) can adsorb water of 0.64 g/g, even at (Figure 17d) 15% of RH, a gyroid structured hydrogel composed of poly(hydroxyethyl acrylate) (PHEA) and polyethylene glycol diacrylate (PEGDA) with LiCl can uptake about 0.15 g/g moisture even in RH of 10% which is a super‐arid condition [3, 166]. Moreover, this 3D printed hydrogel permits loading up to 67% of salt without any leakage or aggregation. To address the salting‐out effect, Sahoo et al., Li et al., Guan et al. introduced polyelectrolyte‐based hydrogels, wherein hygroscopic salts can be immobilized either via strong electrostatic coordination with the ionic segments or through strategically developed network architectures that prevent salt diffusion and leaching [66, 182, 191]. In the super‐hygroscopic hydrogel made up by hybridizing a metal‐organic framework NH_2_‐MIL‐53(Al) with a thermoresponsive valine/2‐hydroxyethyl methacrylate acrylamide (HEMA)–NIPAM and integrating CaCl_2_ as a hygroscopic salt, CaCl_2_ is immobilized via ion‐dipole and electrostatic interactions with NH_3_^+^/amide groups. Where the MOF works as a photothermal agent. The synergistic effect of MOF and polymer gives the hydrogel mechanical integrity during continuous swelling and deswelling cycles by restricting excessive expansion during hydration, which prevents the whole structure from collapsing or phase separation. The rapid sorption kinetics enable the system to reach up to 80%–85% of its equilibrium water uptake within 5 min, and releases nearly 100% of the adsorbed moisture within half an hour and a temperature of 40°C under the sun [191]. Guan et al. and Aleid et al. applied a similar strategy to suppress salt agglomeration and leakage out by introducing LiCl salt in a zwitterion‐based polymeric hydrogel, in which one of the key components is poly‐[2‐(methacryloyloxy)ethyl]dimethyl‐(3‐sulfopropyl)ammonium hydroxide(PDMAPS). In this system, the zwitterionic (‐N^+^(CH_3_)_2_^−^ and ‐SO_3_^−^) clusters immobilize the salt through electrostatic coordination with the Li^+^ and Cl^−^ ions [54, 66]. Whereas Li et al. designed a hollow carbon sphere (HCS) by using polystyrene and phenol‐formaldehyde (PF) resin, and the vacuum was filled with LiCl, shown in Figure 17a,b. The LiCl alone captured moisture from the ambience 100% of its own weight within 3 h, and under RH 60% and desorption happened within 30 min upon application of 1 kW/m^2^ solar irradiation. This structure efficiently uses solar energy because of its photothermal effect and by trapping the salt inside the cavity, which keeps the system stable and leakage‐proof. Confinement of salt within the hollow cavity efficiently immobilizes the salt, which enhances structural stability and prevents leakage of salt [182]. In contrast to all the salt‐loaded hydrogels, Pei et al. developed an alternate strategy of water harvesting by synthesizing an intelligent hydrogel composed of a thermos‐responsive and a pH‐responsive polymer, PNIPAM/PDMAEMA, respectively, without the bottleneck of salt‐solution leakage, corrosion and pore‐blockage [124].

### 4.4 Hydrogel‐MOF and Hydrogel‐Aerogel Hybrids

**FIGURE 18:**
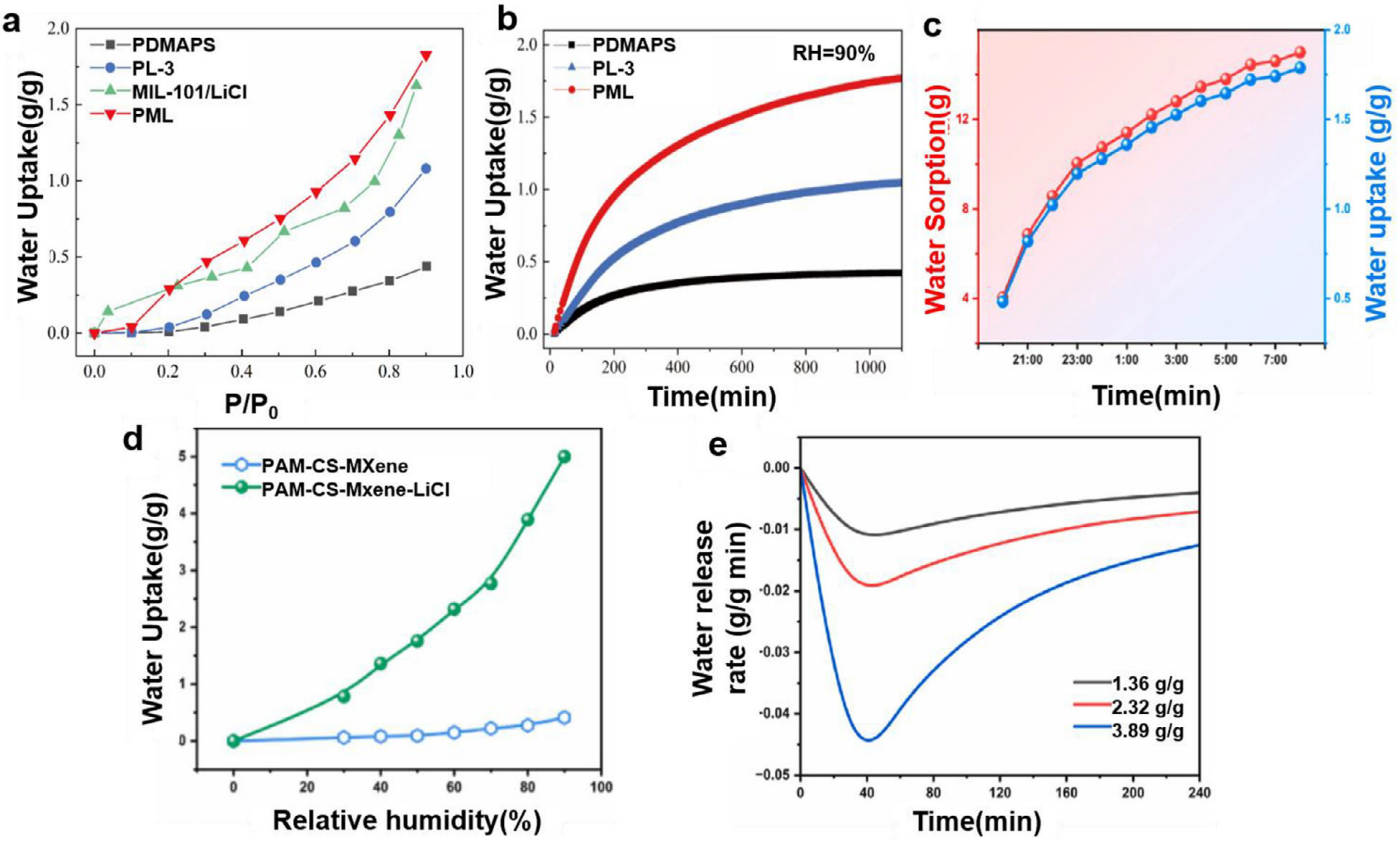
Behavior of Hydrogel‐MOFs (a‐b) and Hydrogel‐Aerogel (c‐e) hybrid sorbents. a) Water vapor adsorption isotherms at 298 K at low RH for water vapor on PDMAPS, PL, PML hydrogels, and MIL‐ 101(Cr)/LiCl. b) Adsorption kinetics at 90% RH demonstrated accelerated vapor uptake in hydrogel‐MOF. Reproduced with permission from ref [94]. MDPI, copyright 2024. c) Water uptake and adsorbed water weight of PAM–CS–MXene–LiCl and PAM‐CS‐MXene. d) Relative humidity dependent water uptake of salt‐modified hydrogel composites. e) Water release kinetics at different water quantities showing diffusion‐controlled, low energy regeneration behavior. Reproduced with permission from ref [192] Elsevier, copyright 2023.

Hybrid sorbents that combine hydrogels with metal–organic frameworks (MOFs) or aerogels have emerged as a one of the most promising strategies to overcome the individual limitations of each component and produce multifunctional platforms for atmospheric water harvesting (AWH) [68, 94]. Hydrogels excel in low‐temperature regeneration, mechanical flexibility and water retention, however their intrinsic polymeric network often exhibits sluggish vapor transportation and limited water capture in lower humid regions. Conversely, MOFs such as MOF‐801, MOF‐303 and CAU‐10 derivatives exhibit exceptional low‐RH adsorption for well‐defined micro‐porous channels and cooperative host‐guest interactions, even they typically require higher regeneration temperatures (≥ 60°C–90°C) and face mechanical challenges as powdered adsorbents [1, 5, 92]. Aerogels like silica, cellulose, graphene where the strengths of one class counterbalance the structural or sorption limitations of the other. Recent studies establish that such hybrids can simultaneously achieve rapid vapor uptake, enhanced moisture transport, improved mechanical durability, and low‐temperature water release features within a single material system. (Figure 18) [68].

Hydrogel‐MOF hybrids represent a successful approach for achieving two‐stage water uptakes. Here, MOF particles are dispersed, embedded or covalently hosted within polymer networks. The MOF component provides a sharp, low‐RH adsorption of strong hydrogen‐bonding interactions and micro‐pore filling, while the hydrogel matrix provides secondary uptake through hydrophilic functional groups and swelling. This combination yields sorption behavior that begins with fast, low‐RH adsorption by MOF and transitions into slower diffusion‐driven hydrogel swelling. (Figure 18a,b) Experimental reports show that such hybrids maintain the high intake of MOFs by reducing their regeneration temperature because the hydrogel stores a significant fraction of water in a weakly bound state [191, 193]. Moreover, embedding MOF particles inside polymer matrices improves its mechanical integrity, reduces particle aggregation, prevents powder loss and limits structural fatigue across cycling, persistent challenges noted for stand‐alone MOF sorbents. In many cases, by mitigating the stresses associated with volumetric moisture loading, the hydrogel network stabilizes the pore environment, which is important information for sustaining cycling durability.

Hydrogel‐aerogel hybrids introduce a different form of synergy to overcome the slow vapor diffusion characteristic of dense hydrogels by investing the macro‐porous architecture of aerogels. Hydrogels are incorporated within silica, cellulose, graphene or carbon aerogels to create composites with interconnected pore networks that accelerate moisture transport by providing structural rigidity. Such hybrids show faster sorption kinetics and more uniform water distribution across the material thickness. (Figure 18c) Studies on cellulose aerogel‐hydrogel composites and carbon aerogel‐hydrogel hybrids demonstrate that aerogels prevent excessive hydrogel swelling. Thereby, it can preserve porosity and improve long‐term cycling stability [94, 192]. Furthermore, when photothermal nanoparticles or carbonaceous aerogels are incorporated, the hybrids show enhanced solar‐driven regeneration due to localized heat concentration within the porous scaffold. (Figure 18e) This design philosophy is reflected in energy‐efficient AWH systems where a single material is capable of moisture capture, water storage, and solar‐triggered release without external energy input [192].

A recent development involves responsive polymers, hygroscopic salts or nano photothermal agents in MOF‐hydrogel or aerogel‐hydrogel matrices to achieve multi‐stimuli water release. For instance, hybrids that incorporate photothermal nanoparticles have demonstrated rapid desorption under natural sunlight, which enables continuous water cycling with minimal energy input [112]. Similarly, zwitterionic and thermoresponsive hydrogel matrices embedded with MOFs or supported on aerogels show humidity‐sensitive transitions (Figure 18a,d), reduced hysteresis and minimized mechanical deformation across cycles [194]. These multifunctional systems represent a shift from simply combining materials to architect synergistic micro‐environments where vapour sorption, transport, confinement, and energy absorption are integrated at multiple length scales.

Briefly, hydrogel‐MOF and hydrogel‐aerogel hybrids illustrate how hierarchical material design can surpass the limitations of single‐component sorbents. Hydrogels contribute tunability, low regeneration temperature and environmental compatibility, whereas MOFs provide precise, low‐RH adsorption, and aerogels offer rapid vapour transport and structural reinforcement. When combined, these materials produce composites with balanced water uptake, enhanced cycling durability and solar compatibility, key requirements for scalable AWH technologies. According to the cited literature, these hybrids are the most advanced sorbent architectures that are currently under development, offering a pathway toward robust, multifunctional, and climate‐adaptive water harvesting systems.

Inclusion of metal‐organic frameworks into hydrogel generates highly functional materials, but there are some critical trade‐offs such as high material cost, fabrication complexity, and industry‐level scalability. Synthesizing a higher‐performance MOF often requires expansive organic linkers and transition metal salts, which makes the raw materials significantly more costly than the host polymer‐based network, which are generally inexpensive [195, 196]. In situ synthesis of crystalline MOF powders, followed by the embedding process in hydrogel through physical or chemical functionalization and uniform dispersion, while maintaining uniform particle size without aggregation, substantially increases fabrication complexity [68]. In contrary in situ production of MOFs demands harsh conditions such as high temperature and pressure, which may degrade the host polymer. Improperly optimized MOF‐based materials are prone to leaching of metal under any aqueous applications, resulting in degradation and compromised cyclic stability [197]. Though researchers are actively exploring green synthesis way and using waste‐driven materials to lower the cost and simplify scaling, but largescale adaptation is still constrained due to the difficulty in mass level production without losing the structural integrity. Furthermore, such a complex fabrication process involves chemical crosslinkers and organic or inorganic solvents which may introduce some toxic impurities which necessitate additional purification before any practical applications [198].

Similar to the hydrogel‐MOF complexes, hydrogel‐aerogel hybrid systems also exhibit several trade‐offs. Their lightweight, highly porous architecture enables excellent water transport and storage capacity. However, the fabrication of high‐performance aerogels often relies on expensive materials such as graphene, MXenes, or aramid nanofibers, etc. and often requires energy‐intensive multi‐step processing (for example, supercritical drying, solvent exchange, gelation, etc) along with reinforcement strategies by using chemical or physical crosslinkers to achieve a uniform pore size with a higher rate of mass transfer and low‐density scaffolds. All these factors collectively increase the fabrication complexity, manufacturing cost and limit industry‐level applications [199, 200, 201].

### 4.5 Emerging Concepts: Biomimetic and Multifunctional Hydrogel Harvesters

**FIGURE 19:**
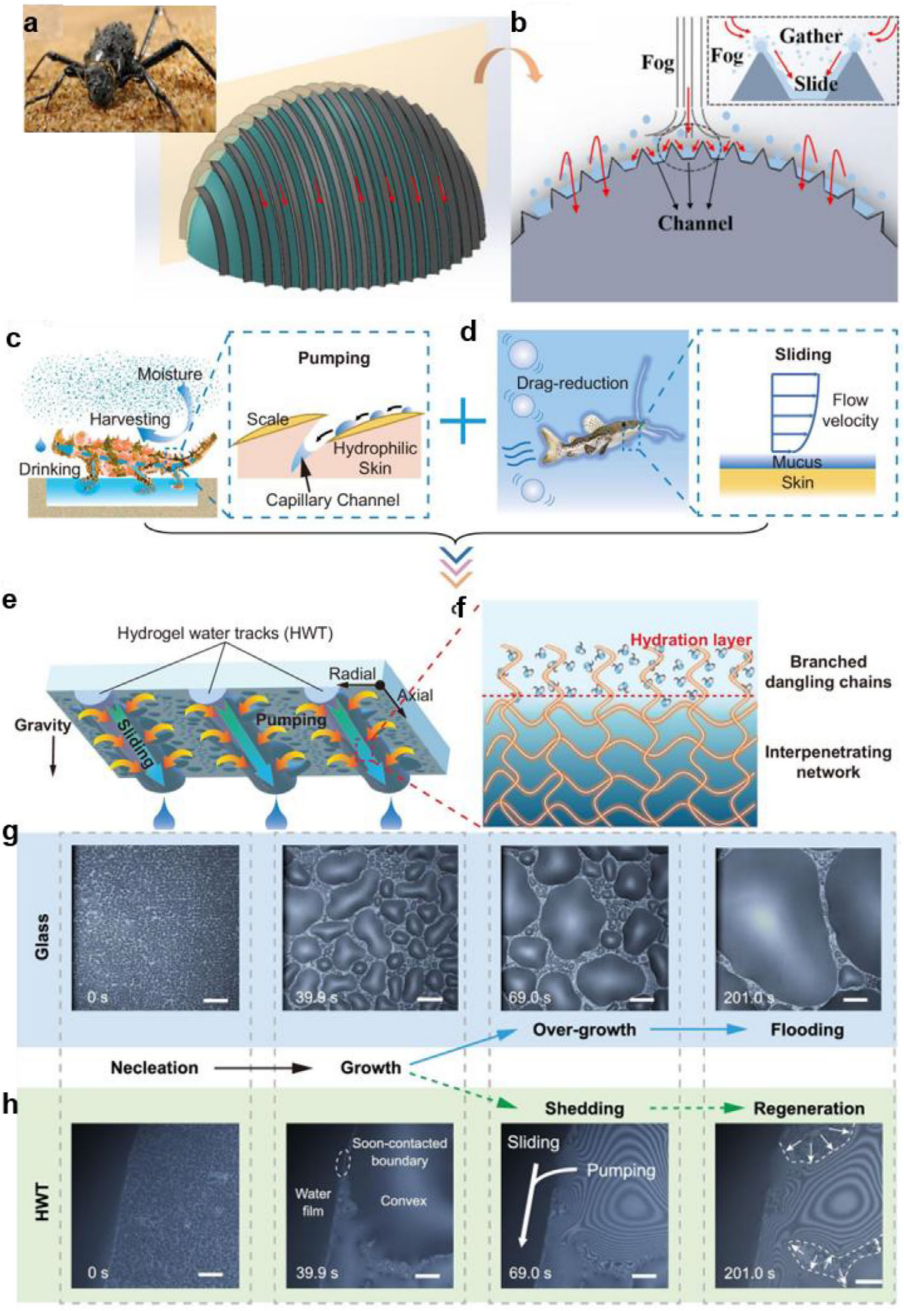
a) Water capture by the Namib beetle via its directionally channelled concave surface. b) Schematic of water transference on the back of a beetle, the red arrows indicate the direction of passage. c) Schematic illustration of the cross‐sectional view of the beetle's dorsal surface during water collection, with the inset depicting the water collection process: adsorption followed by agglomeration and directional transport. Reproduced with permission from ref [171]. Elsevier, copyright 2022. d) Schematic depiction of continuous water capture and directional water transport toward the mouth in moisture‐harvesting lizards, through the capillary channels between the adjacent scales. e) Schematic illustration of the drag‐reduction mechanism in catfish, driven by the presence of a mucus layer on the skin. f) water harvesting track on the silicate glass to collect condensed droplets and regenerate active nucleation sites, the blue semi‐cylinders represent the water harvesting track. g) Model representation of the molecular architecture of hydrogel water tracks (HWTs). h) Optical image droplet nucleation, growth, coalescence and formation of film on bare glass as a control. Reproduced with permission from ref [173]. Oxford academic, copyright 2023.

Though the commercial adsorbent materials such as hygroscopic salts, polymeric hygroscopic composite, silica gel, zeolite MOF, etc, capture atmospheric moisture efficiently, they often suffer from structural degradation due to repetitive adsorption or desorption cycles and sluggish kinetics [202, 203, 204]. The primary step of atmospheric water harvesting is condensation by growth of adsorbed vapor, followed by shedding [173]. But sometimes the nucleation causes flooding on the condensing surface. In addition to the above‐mentioned criteria, the adsorbents must facilitate a uniform size and distribution of droplets with regulated growth and coalescence, while maintaining a lower resistance to vapour and mass diffusion, which could lead to better kinetics [173, 202]. Notably, nature provides various examples of highly efficient mass and heat transport systems, solar evaporators, with a highly organized and ordered structure which facilitates free movement of water and thermal energy. Numerous biological systems exhibit sophisticated strategies and unique topographies in harnessing water resources for survival in diverse geographical regions and challenging environments. Inspired by these natural strategies, scientists have sought to replicate those living systems, such as cactus, skin sweating, hierarchical wing structure of cormorants, moisture‐harvesting lizard, skin of catfish, patterned back of Namib beetles (Figure 19a,b), etc. [109, 157, 171, 173, 205].

Xia et al. reported a water sorption system inspired by a vascular transpiration network of a plant, containing gradually reducing porosity with a multifunctional component. Consisting of a macroporous base layer, intermediate microporous layer, and a submicron top layer with hydrophilic modification, followed by a nanoporous and photothermal layer of coating [206, 207, 208]. This gradual reduction of pore size from macro to micron level facilitates ultrafast mass transportation according to Murray's law of hierarchical transport theory, where comparatively smaller pores generate higher capillary pressure and induce directional water transport. The Laplace pressure generated due to the porous structure of the system which sufficiently exceeds the gravitational force. The uniform distribution of PPy‐COF as a coating elevates the local temperature and accelerates desorption kinetics. As a result, this system can achieve rapid sorption (2.26 g/g at 80% RH within 50 min), and sufficient solar‐driven desorption (95% release of the adsorbed water within 10 min under 1 sun irradiation). This system is able to perform 10 continuous sorption cycles per day and, in outdoor operation, produces 3.91 kg m^−2^ of clear water, which is enough for an adult's regular need, which proves a successful integration of a plant's vascular transpiration network [202]. A geometry‐driven LaPlace pressure gradients can induce a continuous directional transportation of droplet, as demonstrated in conical microstructure inspired by desert plants. Driven by this principle, Shi et al. designed a 3D micro‐tree‐architected hydrogel composed of PVA/PPy. Droplets adhered to the conical structures experience a pressure gradient, which enables them to move toward the wider base from the sharp tip. Additionally, the hierarchically porous hydrogel is beneficial for efficient water transportation throughout the system. Compared with other geometries such as flat surfaces, cylinders, and cones, the fog collection rate of the conical micro‐tree is 5, 3.3 and more than 2 times higher, respectively. This enhanced performance is owing to its larger effective surface area and its ability to promote efficient heat localization at the air‐water interface, which facilitates a rapid evaporation kinetics and higher evaporation efficiency than those of the other geometries [88]. Su et al. biomimetically replicated the structural features of a weed named *S. viridis,* known for its high mechanical strength, tolerance to salt and drought, with excellent fog collection capacities. The authors fabricated the hydrogel by using Polyvinyl alcohol (PVA), chitosan, polyethene glycol (PEG), and Graphene oxide as a photothermal agent. Each of the individual spines features barb‐like tips with inclined grooves along its mid‐section. These micron‐level grooves and vertically aligned passages together provide a rapid, smooth and low‐friction surface for flow of the captured condensed water with minimal resistance, while the Laplace pressure gradient drives the water droplets toward the wider base, enhancing condensation efficiency and enabling directional water transport [169]. Analogously, a human body's average temperature is 37°C, and exposure to higher temperatures induces sweating, which is an evaporative cooling system. This natural thermoregulation principle inspired Ji et al. to develop a human skin‐inspired temperature‐responsive interpenetrating hydrogel comprising Poly(N‐isopropylacrylamide) (PNIPAM) and calcium alginate. The PNIPAM and Calcium alginate generate an eggshell‐like double‐network structure by interweaving through each other, which prevents network collapsing during the continuous sorption cycle and retains its exact hygroscopicity even after 10 sorption cycles. Upon heating beyond the LCST of PNIPAM, it releases the adsorbed water, but Alginate‐Ca^2+^ retains bound water and maintains structural integrity. Even after crossing the LCST, Alginate‐Ca^2+^ retains its hydrophilicity, which prevents any abrupt deswelling or excessive release of water [109]. To address the limitations, of conventional condensation surfaces and the disordered microstructure, which lead to certain drawbacks, such as over‐ or under‐growth of droplets and insufficient driving force, resulting in an uncertain trajectory, surface flooding, deactivation and choking of active condensation sites; Zhang et al. strategically bio‐mimicked the capillary channelled skin texture of the Australian and Texas moisture harvesting lizard to harvest directionally driven droplets with a wide range of diameters (Figure 19c,d), resulting in an increased and efficient storage. Additionally, the authors also replicated the mucus‐coated epidermal layer of catfish for drag‐reduced sliding of droplets through an ultra‐low friction coefficient in an aqueous environment. Sodium acetate and poly (Vinyl Alcohol) were used to design the arch‐shaped geometry of the lizard (Figure 19e), which creates a Laplace pressure difference and surface energy gradient, inducing a directional droplet motion. The surface of the hydrogel was partially polymerized by using branched dangling polymer chains to mimic the super‐hydrophilic mucus of catfish (Figure 19f). This hydrophilic fibrous surface makes a strong bond with the inner layer of water while creating a shear layer for the outer layer of water, enabling rapid sliding off the condensed droplets under gravity with minimal drag resistance (Figures 19g, h). The resulting water harvesting rate was 2.05‐7.12 Lm^−2^ day^−1^ with an increased time gap between the shedding of water droplets. Generally, for solar‐based hydrogel systems, bulk water evaporates by accumulating solar energy; in this process, heat is transformed from the solar‐assisted heated surface to the bulk water of the evaporator via convection or conduction in the evaporation unit. Heat loss to the non‐purified bulk water can be categorized into two different factors: one is heating loss per unit surface area, and the other is heating flow across the cross‐section [173]. To date, most of the research has been focused on reducing the heat loss per unit area by enhancing the heat localization; however, the strategies to minimize the heat loss by reducing heat transfer through the cross‐sectional area have not been well explored yet. As the heat transfer through a material is proportional to the cross‐sectional area, water transfer through the same cross‐sectional area water transport get compromised when the pore size is reduced, and a low rate of water transfer is inefficient for any application. But nature provides us an effective design strategy that minimizes heat transfer across the cross‐section while maintaining sufficient water flux and enabling sustainable water evaporation through localized heat management. Cormorants are characterized by large and slender wings with a hierarchical porous structure channel, which efficiently captures the solar energy and promotes rapid vaporization of water with minimal heat transfer through their wings. Following this rationale, Lei Ma et al. synthesized a very thin (0.3 mm) film of hydrogel with interconnected micro‐pores (50‐100 µm) by crosslinking sodium dodecyl sulfate and lauryl methacrylate. This thin hydrogel provides less material for heat transfer through it, resulting in a reduction of heat loss by 88%. Since heat loss is directly proportional to the thickness of the hydrogel, reducing the thickness enhances the rate of evaporation, i.e. 3.84 kgm^−2^h^−1^ [157].

In analogy, cactus exhibits two distinctive features: highly efficient water absorption coupled with a strong anti‐evaporation capability, and the ability to release stored water in response to environmental and physiological demand. The 3D cactus stem contains interconnected pores that allow an efficient and uniform fog absorption throughout the system and support a sustainable, prolonged storage. The outer superhydrophobic cuticle protects the underlying cactus stem and mucilage. This cuticle layer inhibits the re‐evaporation and redirects the released water inward. Building upon this principle, replication of a unique morphological phenomenon of the cactus was achieved by fabricating a mucilage‐inspired interpenetrating network hydrogel composed of PNIPAAm, calcium alginate and Agarose – combined with a steric acid‐coated copper mesh as a hydrophobic outer layer, can capture hydrophobic moisture and harvests water droplets with a critical diameter due to a proper balance of capillary, adhesion and gravitational forces and retains a large amount of water up to its LCST. Meanwhile, the superhydrophobic cuticle, possessing a large surface area, protects the underlying stem and mucilage by inhibiting the re‐evaporation and redirecting the released water inward, thus the internal water is preserved [209]. A comparative overview of these architectural strategies and their key outcome metrics is provided in Table 8. Collectively, these case studies demonstrate significant progress in hydrogel design and performance, but the fundamental engineering and scalability limitations remain to be addressed.

**TABLE 8:** Comparative case study of biomimetic and multifunctional hydrogel systems for atmospheric water harvesting.

Inspiration	Materials used	Architectural strategy	Driving mechanism	Sorption kinetics
Plant's vascular transportation system [202]	polypyrrole‐ (1,3,5 – triformylphloroglucinol/ p‐phenylenediamine) COF/LiCl	Hierarchically (macro to submicron) aligned pores from macro to nanoporous level Photothermal coating of PPy	Murray's law capillary pressure gradient Laplace pressure difference Photothermal induced desorption	0.7‐2.56 gg^−1^ water uptake at 30–80% of RH within 50 min ∼ 95% desorption within 10 min under 1 sun 3.91 kg m^−2^ day^−1^ water yield
Conical‐shaped micro‐tree [88]	Poly vinyl alcohol‐polypyrrole (PVA/PPy)	3D micro‐tree	Geometry‐driven directional transportation, (5‐, 3.3‐ and 2‐times higher fog collection rate than flat, cylindrical and cone surfaces, respectively – due to increased surface area LaPlace pressure gradient	Fog capture rate ∼5 g.cm^−2^.h^−1^ Moisture generation rate 3.64 kg.m^−2^.h^−1^ under 1 kW/m^2^, outdoor daily water collection rate ∼ 34 Lm^−2^
Weed spine [169]	Polyvinyl alcohol (PVA), chitosan, polyethene glycol (PEG), and GO	spines containing barb‐like tips and inclined grooves	Capillary flow Laplace pressure gradient Photothermal induced desorption	fog collection rate ∼5 g cm^−2^ h^−1^, 3.5 kg.m^−2^.h^−1^ evaporation rate under 1 sun irradiation
Human thermoregulation system (sweating) [109]	PNIPAM‐sodium alginate, CaCl_2_ aqueous solution	eggshell‐like double‐network interweaved hydrogel	Thermally triggered desorption over LCST	Maintain structural stability over 10 continuous cycles moisture adsorption capacity 1.71 g g^−1^ at 95%RH, 25°C
Moisture harvesting lizard with interconnected capillary channels for directional water transportation, combined with lubricating mucus layer of catfish for drag reduced droplet sliding [173]	Sodium acetate /poly (Vinyl Alcohol) hydrogel on silicate glass	Arch shaped geometry with capillary channel	Laplace pressure gradient Surface energy gradient Directional‐driven condensing Lubrication of hydration layer to minimize friction	Sorption kinetics not applicable, ∼9.3s (droplets reach HWT) Water collection rate 85.9%, effective pumping distance 200–500 µm.
Cormorants wing [157]	Sodium dodecyl sulfate and lauryl methacrylate	Very thin film with hierarchical porous channel	Solar driven interfacial evaporation due to localization of heat by minimizing the width of film	Water evaporation rate 4.14 kg.m^−2^.h^−1^ under 1 sun irradiation Reduction of heat loss by ∼88% Increased rate of evaporation 3.84 kgm^−2^h^−1^
Cactus stem [209]	PNIPAAm, Calcium alginate, Agarose and stearic acid coated with copper mesh	Interpenetrating network mucilage core and steric acid‐coated copper mesh act as a hydrophobic (Inhibition of re‐evaporation, high water retention) outer layer	Temperature‐stimulated desorption Anti‐evaporation barrier, capillary force, gravity driven drainage	10 sorption cycles, Water harvesting rate 209 mg.cm^−2^h^−1^
Namib beetle's dorsal surface [171]	3D printed triangular convex structure PVA hydrogel	directionally channelled concave surface with constant wettability	Laplace pressure Capillary transportation and condensation Directional drainage driven by gravity	Stable water collection for ≥ 10 successive cycle, Collection of 16.65 g of water in 2 h

## 5 Practical Challenges and Limitations

Along with all the advantages, polymeric water harvesting systems often face challenges due to the lower efficiency of hydrogel systems in lower humidity environments, lower adsorption capacity at higher temperatures, and very high or very low crosslinking density, which affects the mechanical properties, elastic mobility, swelling ratio, and adsorption capability, etc. [91].

- A mechanically weak hydrogel often undergoes fatigue due to repetitive cycles, which disrupts its structural integrity and lowers its adsorption and swelling capacity. These challenges can be addressed by synthesizing a hydrogel with one or more polymeric networks, or an interpenetrating network or using a strategic design, such as a core‐shell structure with reversible or sacrificial bonding, which helps to increase the elongation at break, toughness, and fractural stress [108, 210].
- Though hygroscopic salts are a cost‐friendly option for water sorption and show a very good sorption capability, the leakage of salt solution, salting‐out or leaching, blockage of the diffusion pathway because of agglomeration, crystallization of salt‐layer on the outer hydrogel surface, resulting in a declined performance. This requires management in salt concentration, immobiliztization of salt, or just incorporation of salt in a crosslinked network in a certain way, which can prevent the above‐mentioned challenges [41, 66, 166].
- Mismatching the balance between water adsorption and desorption is another important issue. Currently, researchers are only focused on an increased uptake rate and swelling capacity in arid conditions, whereas increased thickness, lack of an interconnected and hierarchical network, inclusion of large sorbent particles block the adsorption efficiency. A hydrogel surface based on salts can effectively adsorb water, but once the surface reaches its saturation point, it blocks further penetration of water inside the hydrogel network, resulting in a slow diffusion process.
- Another important drawback is the limited long‐term stability of polymer‐based water harvesting hydrogels. Most researchers have investigated to increase the cyclic durability of hydrogels, but the number of cycles is not higher than 10–20 [53]. To address this mechanical instability, scientists proposed several chemical or architectural strategies, such as double‐network or interpenetrating polymer networks. Maity et al. synthesized a core‐shell structured hydrogel, composed of sulfonic acid modified PNIPAM‐Polyaniline‐Alg‐LiCl enables it to retain more than 84% of its initial water capacity even after completing 8 complete cycles for 30 days [211]. Another super‐hygroscopic amorphous inorganic hydrogel composed of Zn and O facilitates water adsorption for more than 420% of its own weight [212]. Also, this zinc‐based hydrogel shows exceptional durability of more than 1000 cycles. This long‐term durability is attributed to the stable inorganic coordination network, which undergoes minor structural rearrangements during the sorption‐desorption cycle; consequently, this material experiences less mechanical fatigue and facilitates water harvesting performance for a longer time duration. More recently, recycled PANSAP‐based hydrogel demonstrated long‐term stability, and scientists claimed a projected service life of exceeding 2500 complete cycles on the basis of one year of operational analysis [98]. Nevertheless, such prolonged cyclic durability data are still scarce for polymeric hydrogel systems, highlighting the need for rigorous assessment of mechanical stability, salt retention, long term robustness and high‐yield water harvesting performance under practical operating conditions.
- Polymer‐based hydrogels have established their efficiency in laboratory‐based experiments, but reports presenting quantitative evidence regarding their long‐term outdoor durability is still limited. Recent studies are moving their focus to improving outdoor durability by using biodegradable polymer matrices for hydrogels and exceeding ∼20‐24 continuous cycles under solar irradiation without any leakage of salt solution while maintaining the same water yield for each cycle [96, 213]. Despite all of this encouraging progress, long‐term outdoor durability remains as one of the least studied fields. In outdoor conditions, the effects of repeated swelling and deswelling, UV irradiation, rainfall, temperature fluctuation, wind speed, migration of salt and durability for at least one long agricultural season are rarely reported.
- Articles on hydrophilic thermoresponsive polymer PNIPAm‐MOF hydrogel, or hydrogel composed of photothermal (CNT, graphene oxide) materials with ionic salts (LiCl, CaCl_2_) have established that even at lower relative humidity (30% RH) the water uptakes approximately 1.36–1.81 g g^−1^, but this hydrogel only enables > 6.6 g_water_ g_sorbent_^−1^ at 90% RH, with a daily water production ranging from 2820 mL_water_ kg_sorbent_^−1^ day^−1^ to 4.16 g g^−1^ day^−1^ (4.16 mL_water_ kg_sorbent_^−1^ day^−1^) and a continuous cyclic sorption‐desorption process for up to 480 cycles [139, 214, 215]. Despite considerable progress, the current performance of polymeric hydrogels is still not sufficient to fulfil requirements beyond a person's daily drinking needs. It is inadequate to meet larger daily water demands while maintaining proper sorption kinetics just above the LCST.
- Beyond the adsorption capacity, water retention, regeneration efficiency, sorption kinetics and cyclic stability, the chemical or microbiological quality of the harvested water remains an important yet relatively unexplored aspect of water harvesting processes. Microbial contamination may occur during moisture adsorption from air, condensation collection, long‐term operation, during water desorption or storage [216, 217]. Recent studies show that during evaporation at around 40°C–50°C gram‐positive bacteria, such as *Staphylococcus aureus,* can be transferred from a swollen aerogel to a water container by evaporation and condensation [216]. According to the WHO guidelines, drinking water should be free from any kind of pathogens or microorganisms [23]. Despite exhibiting a promising antimicrobial performance against individual bacterial strains under controlled laboratory conditions, the efficacy of the system under realistic environments or against multispecies microbial communities remains to be validated.

Researchers have stated that hydrogels in the laboratory scale are already suffering from sorption kinetics, salt leakage, network aggregation, high tortuosity factor, heterogeneous crosslinking density, unoptimized thickness, non‐uniform or extreme mechanical properties, fatigue, generation of microcracks, and poor outdoor durability. These issues are collectively the main bottlenecks for large‐scale production, commercialization, and application [58].

## 6 Conclusion and Future Perspectives

This review comprehensively examines current progress in polymer‐based hydrogels for SAWH, exhibiting the interplay between sorbent chemistry, hydrogel architecture and water harvesting performance. It emphasizes polymer design strategy, hierarchical porosity and externally triggered materials that facilitate efficient water yield and low desorption enthalpy while overcoming conventional limitations. The inclusion of functional nanomaterials in a biobased polymeric host further enhances sustainability, scalability, as well as applicability while promoting environmental compatibility. Future studies should emphasize integration of advanced modelling approaches, molecular simulations, and artificial intelligence‐guided architectural design to develop scalable, durable and energy‐conserving hydrogel sorbents for atmospheric moisture collection. Polymer–based atmospheric water harvesting (AWH) systems have advanced rapidly in recent years, evolving from laboratory‐scale hygroscopic materials to increasingly integrated material‐device platforms capable of operating across diverse climatic conditions.

For further development of this AWH technology, the following suggestions can be used.

First, hydrogels should be tuned in specific ways to facilitate water uptake under ultra‐low relative humidity (10%–20% RH) through rational design of bidirectionally aligned and hierarchical porous architecture, the incorporation of zwitterionic polymers or the insertion of a multifunctional material into the polymeric matrix. Computational approaches such as multiscale molecular dynamics simulations can further unravel the inter or intra‐molecular interactions and facilitate the rational selection of functional groups to promote water sorption performance.

Second, long‐term mechanical stability should be systematically investigated under realistic outdoor conditions involving swelling‐deswelling (> 1000) cycles, combined with all kinds of environmental stressors, such as UV radiation, thermal cycling, high‐speed wind exposure, and widely fluctuating temperatures. Future polymeric atmospheric water harvesting hydrogels should aim to cross a specific quantitative performance benchmark, such as a swelling ratio of ∼10 g g^−1^ at 30% RH, with a controlled water regeneration rate at a temperature lower than 50°C, exceeding a long‐term cycle stability > 500 cycles without any mechanical degradation or salt leakage.

Thirdly, the incorporation of a single stimulus‐responsive material is expected to extend its application beyond water harvesting, focusing on harvesting water from the ocean surface, water treatment, ecological remediation, and the development of smart windows, envelopes, and rooftop systems that combine water harvesting with passive cooling and humidity regulation.

Another promising avenue involves developing temperature‐controlled, stepwise desorption of adsorbed water. This approach may facilitate progressive water recovery with optimized use of energy.

Fourthly, to address the microbial contamination issue, next‐generation AWH systems should integrate some bio‐based broad‐spectrum antimicrobial agents capable of capturing and inactivating pathogens. Therefore, future research should focus on the inclusion of antimicrobial materials or advanced machinery for constant monitoring of the safety parameters of harvested water before use. Development of smart pathogen‐responsive indicators, which are capable of detecting contamination by changing their colour or other readily detectable signals, could provide a rapid on‐site assessment of the water quality. Materials with antimicrobial durability should be developed to ensure adherence to WHO drinking guidelines for human consumption, or any other biomedical and agricultural applications.

Finally, a scalable and cost‐effective fabrication process should be developed by utilizing renewable bio‐based polymers or recyclable synthetic polymers to minimize production cost without compromising the structural integrity and sorption performance (Supporting information).

## Author Contributions

**Nilanjan Dey**: conceptualization, Writing – original draft, methodology, software, Writing – review and editing, visualization. **Sushmit Sen**: writing – original draft, conceptualization, methodology, software, visualization, writing – review and editing. **Ananya Sadhu**: writing – original draft, visualization, writing – review and editing. **Pradip K. Maji**: conceptualization, funding acquisition, writing – review and editing, supervision, resources, formal analysis, project administration. **Swagata Datta**: conceptualization, investigation, writing – original draft, methodology, visualization, writing – review and editing, software, formal analysis. **Amrita Chatterjee**: conceptualization, writing – original draft, methodology, writing – review and editing, visualization, software. **Sudipta Sarkar**: conceptualization, writing – review and editing, project administration, supervision.

## Conflicts of Interest

The authors declare that they have no known competing financial interests or personal relationships that could have appeared to influence the work reported in this paper.

## Supporting information




**Supporting File**: advs77343‐sup‐0001‐SuppMat.docx.

## Data Availability

Data sharing not applicable to this article as no datasets were generated or analysed during the current study.
