# Supplementary material for: Atmospheric Water Harvesting Through Polymer Hydrogel Sorbents: Materials Chemistry, Design Strategies, and Applications
Source: Adv Sci (Weinh). 2026 Aug 27:e77343. Online ahead of print. doi: 10.1002/advs.77343 (PMC13521032; doi:10.1002/advs.77343)
Supplement: Supplementary file 1 [file ADVS-9999-e77343-s001.docx]

**Swagata Datta** is a doctoral researcher in the Department of Polymer and Process Engineering at the Indian Institute of Technology (IIT) Roorkee, under the supervision of Prof. Pradip K. Maji. Her research focuses on the synthesis and functional modification of polymer-based hydrogels for atmospheric water harvesting. She holds a B.Tech. in Polymer Science and Technology from the University of Calcutta and an M.Tech. in Materials Science and Engineering from IIT Kanpur. Her M.Tech. research involved the fabrication of PVDF - based composites incorporated with piezoelectric materials to enhance the piezoelectric performance of the system for actuator applications. Her current research focuses on the design of hygroscopic polymeric hydrogel systems integrated with stimuli-responsive materials for efficient atmospheric water harvesting.

**Sushmit Sen** is a doctoral researcher in the Department of Polymer and Process Engineering at the Indian Institute of Technology (IIT) Roorkee, under the supervision of Prof. Pradip K. Maji. His research focuses on the synthesis and functional modification of polymeric materials for advanced interface engineering in applications such as corrosion protection, energy storage, and water management. He holds a B.Tech. in Polymer Science and Technology from the University of Calcutta and an M.Tech. in Polymer Engineering and Science from the Institute of Chemical Technology (ICT), Mumbai. His work emphasizes the design of multifunctional polymer systems through controlled copolymerization and surface functionalization, integrating reactive monomers, nanofillers, and bio-based crosslinkers to tailor interfacial properties like adhesion, wettability, and charge transfer for practical engineering solutions.

**Nilanjan Dey** is currently enrolled as a PhD student at the Department of Polymer and Process Engineering, Indian Institute of Technology Roorkee, India, under the supervision of Prof. Pradip K. Maji. He received his Bachelor of Science degree from the University of Calcutta and a Master of Science degree from the Indian Institute of Technology Kharagpur. He has a 2-month research internship experience from IISER Kolkata and 1 year of research experience from the Indian Institute of Technology Bombay. His research interest focuses on extracting plant-based materials and valorizing them to fabricate high-end products for the construction industry.

**Amrita Chatterjee** is a research scholar in the Department of Polymer and Process Engineering, Indian Institute of Technology (IIT) Roorkee, Uttarakhand, India. She earned her Bachelor's and Master's degrees in Polymer Science and Technology from the University of Calcutta and the Institute of Chemical Technology, Mumbai. Her research focuses on the design and development of advanced polysilazane- and polymer-based multifunctional coatings with applications in anti-icing, anti-graffiti, and flexible energy devices. Her work integrates polymer chemistry, surface science, and materials engineering to achieve durable, sustainable, and high-performance coatings. Ms. Chatterjee's research is distinguished by active collaboration between academia and industry. She has presented her research at several reputed national and international conferences and has received recognition for her technical contributions and presentations. Her work bridges interdisciplinary research with industrial relevance, aiming to advance next-generation protective and functional coating systems.

**Ananya Sadhu** has completed her Bachelor of Science (B.Sc.) in Chemistry from Banwarilal Bhalotia College under Kazi Nazrul University, Asansol, West Bengal. She further pursued her Master of Science (M.Sc.) in Chemistry with a specialization in Inorganic Chemistry from Kazi Nazrul University, where she secured the top position in her department.

Her academic training includes hands-on experience in the synthesis of metal–organic frameworks (MOFs), with a focus on developing functional materials for advanced applications. She has demonstrated consistent academic excellence and a keen interest in advanced research areas. Her work reflects a commitment to scientific rigour and innovation, with a focus on advancing emerging developments in the chemical and materials sciences.

**Sudipta Sarkar** is a Professor of Environmental Engineering within the Department of Civil Engineering at Indian Institute of Technology Roorkee. He earned his PhD from Indian Institute of Engineering Science and Technology, Shibpur, India. His research interests are valorization of industrial, agricultural and municipal waste, application of nanotechnology in environmental systems, trace and bulk contamination removal from impaired water, and nutrient recovery from wastewater.

**Pradip Kumar Maji** is an Institute Chair Professor at the Indian Institute of Technology, Department of Polymer and Process Engineering in Roorkee, India. He earned his PhD in Polymer Science and Technology from the Indian Institute of Technology in Kharagpur. His research areas of interest include waste valorization, cellulose-based biopolymers, functional nanomaterials, anti-counterfeiting, superthermal insulation, supercapacitors, oil–water separation, and rubber technology. In 2022, he was awarded the JSPS Invitational Fellowship by the Japan Society for the Promotion of Science. He worked as a Scientist-C at the Defence Research and Development Organisation (3 Base Repair Depot, Indian Air Force, Chandigarh, India). He was awarded the Outstanding Teacher Award from IIT Roorkee and the Young Scientist Award by the Asian Polymer Association in 2023. He was on the list of Elsevier/Stanford's top 2% scientists for the last four years. He has published over 200 papers in refereed journals and is an associate editor in the Sustainable Chemistry and Pharmacy journal of Elsevier.
